# Systems-Based Approach for Optimization of Assembly-Free Bacterial MLST Mapping

**DOI:** 10.3390/life12050670

**Published:** 2022-04-30

**Authors:** Natasha Pavlovikj, Joao Carlos Gomes-Neto, Jitender S. Deogun, Andrew K. Benson

**Affiliations:** 1 School of Computing, University of Nebraska-Lincoln, Lincoln, NE 68588, USA; npavlovikj@unl.edu (N.P.); deogun@cse.unl.edu (J.S.D.); 2 Department of Food Science and Technology, University of Nebraska-Lincoln, Lincoln, NE 68588, USA; jgomesneto2@unl.edu; 3 Nebraska Food for Health Center, University of Nebraska-Lincoln, Lincoln, NE 68508, USA

**Keywords:** multi-locus sequence typing, MLST, stringMLST, ProkEvo, k-mer lengths, zoonotic pathogens, genomic epidemiology, surveillance, parameter-tunning, whole-genome sequencing, public health

## Abstract

Epidemiological surveillance of bacterial pathogens requires real-time data analysis with a fast turnaround, while aiming at generating two main outcomes: (1) species-level identification and (2) variant mapping at different levels of genotypic resolution for population-based tracking and surveillance, in addition to predicting traits such as antimicrobial resistance (AMR). Multi-locus sequence typing (MLST) aids this process by identifying sequence types (ST) based on seven ubiquitous genome-scattered loci. In this paper, we selected one assembly-dependent and one assembly-free method for ST mapping and applied them with the default settings and ST schemes they are distributed with, and systematically assessed their accuracy and scalability across a wide array of phylogenetically divergent Public Health-relevant bacterial pathogens with available MLST databases. Our data show that the optimal k-mer length for stringMLST is species-specific and that genome-intrinsic and -extrinsic features can affect the performance and accuracy of the program. Although suitable parameters could be identified for most organisms, there were instances where this program may not be directly deployable in its current format. Next, we integrated stringMLST into our freely available and scalable hierarchical-based population genomics platform, ProkEvo, and further demonstrated how the implementation facilitates automated, reproducible bacterial population analysis.

## 1. Introduction

Modern epidemiological investigation of bacterial pathogens relies on rapid genomic characterization of new isolates routinely received by Public Health laboratories, along with bioinformatics programs for classification/comparison of genomic data from new isolates to existing data from many thousands of isolates [1,2]. Although surveillance, source-tracking, and attribution are primary goals for the use of whole-genome sequencing (WGS) data by Public Health agencies, there is growing interest in using the WGS data from thousands to hundreds of thousands of isolates of a given pathogen to study hierarchical genotypic classification at different levels of resolution and prediction of population-specific traits contributing to virulence, ecological fitness, or antimicrobial resistance (AMR) [3].

Evolutionarily related bacterial populations (e.g., species and subtypes of a species) share common genotypic backbones, and the degree of clonal relatedness of a population provides a context for studying co-inheritance of core and accessory genes [4,5,6,7]. Core loci are shared by at least 99% of the genomes, whereas accessory loci represent a sparse ensemble that is not shared by all variants of a population, but jointly, the core and accessory genomic content from a given species forms its pan-genome [8]. Population genetic analysis of bacterial species using multi-locus methods have shown that multiple types of genotyping schemes can provide sufficient resolution for classifying populations based on genotypes beneath the species level [9,10,11,12], and the genetic relationships between different populations reveal the pattern of population diversification and structuring. This optimal level of genotypic resolution can be considered an informative genotypic unit that facilitates both ecological and epidemiological inquiries [4,5,6,7,8,9,10,11,12,13,14,15].

Multi-locus sequence typing (MLST) is a well-established and widely used genotyping technique that classifies bacterial genomes into sequence types (ST) [9,12]. ST classification based on traditional MLST is usually inferred from seven loci that are found ubiquitously in the species and are scattered around the genome. Highly curated, species-specific databases of allelic variation at MLST loci and distributions of STs are publicly available [8,16,17]. Historically, sequences for MLST loci were generated from locus-specific polymerase chain reaction (PCR) assays, but now are typically inferred from assembled WGS data [9,18,19]. ST-based classification provides useful and relevant genotypic units for epidemiological surveillance, population genetic analysis, and evolutionary inference. Relative to surveillance, ST-based genotyping standardizes the nomenclature for intra- and inter-laboratorial diagnostics and epidemiological inquiries worldwide [18,20,21]. With respect to evolutionary inference, isolates sharing alleles at five or more loci are more likely to be ancestrally related and are commonly classified as members of a clonal complex (i.e., a group of STs that have shared a common ancestor very recently) [9,18,19,22]. The ST and clonal complexes provide a point of reference for genetic analysis (e.g., linkage disequilibrium—LD) to track inheritance/co-variance of accessory loci among different STs or clonal complexes. For example, LD analyses of pan-genomic content can inform phenotypic predictions for the inheritance of unique traits in an ST related to virulence, environmental stability, or AMR [9,18,19,20,21,22,23,24,25].

Recent studies have shown that LD between specific sets of accessory genes such as AMR genes is an intrinsic property of some STs of specific bacterial pathogens, and rapid identification of a given ST from WGS data can quickly provide accurate prediction of AMR profiles [25,26]. There are important applications of this innovative strategy for the treatment of patients, and there is growing interest in defining LD relationships of AMR genes and virulence-associated genes with STs. A limiting factor in ST-based classification from WGS data, however, is the dependence on genome assembly, most often from Illumina-based short-read data [8,16,17]. This bottleneck can also hinder ST-based surveillance efforts when many thousands of genomes are involved in real-time analysis [27,28]. One approach to overcoming the computational bottleneck is to use k-mer-based ST classification directly from Illumina paired-end raw reads (assembly free). However, these approaches have not been broadly evaluated across data from multiple Public Health-related bacterial pathogenic species. Given the remarkable variation in the intrinsic features of genomes from different species (e.g., compositional features, abundance of repetitive sequences, mutation rates, and rates of recombination), it is likely that parameters for assembly-free algorithms will need to be empirically optimized for genetically diverse species and even subtypes (e.g., serotypes) of pathogens.

There are multiple tools available for MLST classification, such as mlst [10], ARIBA [29], stringMLST [30], MentaLiST [31], and STing [32]. In general, the available tools can be categorized based on the input data they use—some tools use raw Illumina paired-end sequence data, whereas others use de novo assemblies [27]. Using raw sequence data for ST-based classification has a tremendous advantage, especially in pathogen surveillance, since all the computationally demanding steps prior to the de novo assembly are bypassed and the ST calls are made as the sequence reads are generated. Specifically, mlst uses de novo genome assemblies as an input and performs mapping in order to align sequences to pre-downloaded allelic files across all target loci. ARIBA identifies AMR-associated genes, single nucleotide polymorphisms, and ST calls using Illumina paired-end raw sequencing reads. ARIBA clusters the raw reads by mapping them to genes, and then performs local assembly within clusters to identify AMR genes and ST calls. On the other hand, stringMLST and MentaLiST rely on k-mer matching between raw sequence reads and available ST schemes that allows for fast mapping and ST-based typing. In a narrow setting with a few bacterial species, both tools are shown to be accurate and fast for standard MLST classification while providing comparable accuracy with MentaLiST, albeit using fewer computational resources [31]. STing is the successor of stringMLST—it uses the same algorithmic approach with additional computational applications for large MLST schemes such as ribosomal MLST (rMLST) and core-genome MLST (cgMLST) [32]. All these tools have integrated ST schemes and/or provide utilities for downloading the available PubMLST schemes. There are a few available comparisons of assembly-free tools for ST classification, and the published studies are primarily focused on the computational resources used and the percentage of correctly classified STs in relatively small data sets [27,31,32]. When tools were tested with real outbreak datasets (*Listeria monocytogenes*, *Escherichia coli*, *Campilobacter jejuni*, *Salmonella enterica*) comprising 85 samples, stringMLST showed the fastest running time of 80.8 min and high accuracy in ST calls (100%) [27]. Although MentaLiST does not scale well when reads with high coverage are used, it performs well on MLST schemes with up to a few thousand genes and alleles, such as cgMLST (~3000 genes) [31]. Although most ST tools perform satisfactorily, there are some relevant bottlenecks to be considered. For example, some tools use out-of-date MLST schemes that require manual curation, and can directly affect the accuracy of ST calls, especially when mixed and low-coverage samples are used [27]. ST tools that are assembly and alignment free, such as stringMLST, STing and MentaLiST, show quite a few advantages in terms of accuracy and efficiency that make them applicable for real-time molecular epidemiology and surveillance. Thus, we chose stringMLST as a representative of the k-mer-based ST tools to perform a systems-based comparative analysis that assessed the computational and statistical efficacy of ST calls across divergent pathogens in contrast to the legacy MLST approach. Here, mlst and stringMLST were applied with their default distributions and PubMLST schemes since we attempted to mimic how users (e.g., microbiologists and molecular epidemiologists) would approach their utilization in a diagnostic or surveillance setting.

In this paper, we systematically examined the performance of stringMLST across a phylogenetically diverse set of bacterial pathogens that are of primary interest to Public Health. Our systematic approach compared the accuracy of classifications using the standard MLST program (used worldwide) vs. stringMLST at varying k-mer lengths across many thousands of genomes from 15 different pathogenic species from three highly divergent phyla. The two programs were used as they are distributed and deployed, with their default settings. Performance was first evaluated across a broad spectrum of phylogenetic diversity using genomes from isolates of *Acinetobacter baumannii *(phylum: Proteobacteria), *Clostridium difficile *(phylum: Firmicutes), *Enterococcus faecium* (phylum: Firmicutes), *Escherichia coli* (phylum: Proteobacteria), *Haemophilus influenzae* (phylum: Proteobacteria), *Helicobacter pylori* (phylum: Proteobacteria), *Klebsiella pneumoniae* (phylum: Proteobacteria), *Mycobacterium tuberculosis* (phylum: Actinobacteria), *Neisseria gonorrhoeae* (phylum: Proteobacteria), *Pseudomonas aeruginosa* (phylum: Proteobacteria), *Streptococcus pneumoniae* (phylum: Firmicutes), *Campylobacter jejuni* (phylum: Proteobacteria), *Listeria monocytogenes* (phylum: Firmicutes), *Salmonella enterica* (phylum: Proteobacteria), and *Staphylococcus aureus* (phylum: Firmicutes). To evaluate performance within diverse populations of single species, we also measured performance of different k-mer lengths across 23 of the most relevant serovars of *Salmonella enterica* subsp. *enterica* lineage I (*S. enterica*). Our results show that optimal performance of stringMLST can, for some species, be achieved with a single k-mer length. However, several different bacterial species, and even different serovars of *S. enterica*, require species or serovar-specific k-mer lengths for enhancing the accuracy in ST classifications. Based on these findings, we further implemented stringMLST into the scalable ProkEvo platform [28], which produces hierarchical genotypic classifications from raw WGS data, and demonstrated how use of species-specific k-mer settings for stringMLST enhances computational performance of the platform.

## 2. Materials and Methods

### 2.1. Datasets Used for Narrow-Scope Analysis

WGS data from four major bacterial pathogens, including *Campylobacter jejuni*, *Listeria monocytogenes*, *Salmonella enterica* subps. *enterica* lineage I (*S. enterica*), and *Staphylococcus aureus*, were selected to be used in the first part of this study. All publicly available raw paired-end Illumina reads for these organisms were downloaded from NCBI using parallel-fastq-dump [33]. Genomes used for all analyses were randomly selected from a previously downloaded sample of isolates containing *C. jejuni* (*n* = 21,919 genomes), *L. monocytogenes* (*n* = 19,633 genomes), *S. enterica* (*n* = 25,284 genomes), and *S. aureus* (*n* = 11,990 genomes) that were processed through the computational platform ProkEvo [28]. Specifically, our study design was comprised of random sampling of 600 genomes from each species, except for *S. enterica*, for which 600 genomes were randomly drawn per serovar (the list of all 20 serovars is shown in Table S1). For each species and all *S. enterica* serovars, all ~600 genomes were randomly split into three independent batches, with ~200 genomes each. The batches were created to measure the degree of variation in classification accuracy when comparing the two ST-based genotyping programs. Whereas for the majority of *S. enterica* serovars there was a total of 600 genomes available, the total number of raw reads publicly available on NCBI and ultimately used for the analyses for *S*. Agona, *S*. Derby, *S*. Johannesburg, *S*. Mbandaka, and *S*. Senftenberg was 565, 590, 534, 535, and 563, respectively. The final total number of genomes used per species was *C. jejuni* (*n* = 600), *L. monocytogenes* (*n* = 600), *S. enterica* (*n* = 11,787), and *S. aureus* (*n* = 600). The text file containing all genome NCBI-SRA identifications is available here: https://figshare.com/articles/dataset/_/16735411 (accessed on 20 February 2022).

### 2.2. Software Tools

#### 2.2.1. mlst

mlst is a standard approach for scanning genome assemblies against traditional PubMLST typing schemes in order to get ST classifications [10]. mlst version 2.16.2 was installed using Anaconda [16] and genome assemblies were used as an input. In order to generate assemblies from the raw Illumina paired-end reads, multiple pre-processing steps were performed. Quality trimming and adapter clipping were performed using Trimmomatic [34], and FastQC was used to check and verify the quality of the trimmed reads [35]. The paired-end reads were assembled de novo into contigs using SPAdes with the default parameters [36]. The quality of the assemblies was evaluated using QUAST [37]. The information obtained from QUAST was used to discard assemblies with 0 or more than 300 contigs, or assemblies with N50 value of less than 25,000 [28]. Finally, the assemblies that passed the quality control were used with mlst, where they are categorized into specific variants based on the allele combinations from seven ubiquitous housekeeping genes [10]. A list of the exact versions of the bioinformatics tools used for generating assemblies for mlst are shown in Table S2. We used mlst with the default options (e.g., mlst --legacy --scheme <scheme> --csv <assembly.fasta> > <output>) and the following schemes: “senterica” (for *S. enterica*), “campylobacter” (for *C. jejuni)*, “lmonocytogenes” (for *L. monocytogenes*), and “saureus” (for *S. aureus*). The distribution of mlst comes with set of pre-downloaded ST schemes. More details about these MLST schemes, such as the number of alleles in the seven genes and the number of ST classifications available, are shown in Table S3. To obtain the ST classifications of all datasets, mlst was run as part of the computational platform ProkEvo [28]. Additionally, a separate run of the mlst program was used to conduct a pairwise comparison between the computational performance (runtime and memory usage) of mlst and stringMLST. The used MLST script can be found here: https://github.com/npavlovikj/MLST_stringMLST_analyses/blob/main/scripts/mlst.submit (accessed on 20 February 2022).

#### 2.2.2. stringMLST

stringMLST is an assembly- and alignment-free rapid tool for ST-based classification of Illumina paired-end raw reads based on k-mers [30]. For the analyses performed in this paper, we used stringMLST version 0.6.3. stringMLST was installed using Anaconda [36]. The first step of using stringMLST was to download the respective MLST scheme from PubMLST. In order to do this, a species name and a k-mer length were needed. The default k-mer length used and suggested by the developers of stringMLST for reads with lengths between 55 and 150 base pairs or nucleotides is 35 (a common read length for Illumina paired-end reads) [30]. We used stringMLST with the default options (e.g., stringMLST.py --getMLST -species <species_name> -P <output_prefix> -k <k-mer>) and the following species names: “Salmonella enterica”, “Campylobacter jejuni”, “Listeria monocytogenes”, and “Staphylococcus aureus”, and k-mer lengths of 10, 20, 30, 35, 45, 55, 65, 70, 80, and 90, independently (https://github.com/npavlovikj/MLST_stringMLST_analyses/blob/main/scripts/stringMLST_dbs.submit, accessed on 20 February 2022). More details about the downloaded MLST schemes, such as the number of alleles in the seven genes and the number of ST classifications available, are shown in Table S3. After the MLST scheme was downloaded and prepared, the final step was to run “stringMLT.py –predict” for the ST classification. To do so, we ran stringMLST with the databases created a priori and the respective paired-end raw reads and k-mer lengths of 10, 20, 30, 35, 45, 55, 65, 70, 80, 90, independently (e.g., stringMLST.py --predict -d <directory_raw_reads> -p -r -t -x -P <database_prefix> -k <k-mer> -o <output>) (https://github.com/npavlovikj/MLST_stringMLST_analyses/blob/main/scripts/stringMLST.submit (accessed on 20 February 2022)). Our choice of using an increasing gradient of k-mer lengths was to evaluate whether the k-mer length parameter could be optimized to enhance ST-based classification accuracy across bacterial species. Lastly, stringMLST was also integrated as part of the computational platform ProkEvo for a rapid ST-based genotyping as part of a hierarchical genotypic scheme [13,28]. This implementation can be found here: https://github.com/npavlovikj/MLST_stringMLST_analyses/tree/main/Prokevo_stringMLST (accessed on 20 February 2022).

### 2.3. ProkEvo-Based MLST Classifications

In order to compare the ST-based classification accuracy and conduct other statistical analysis (e.g., identifying major contributing factors influencing ST-based classifications) between mlst version 2.16.2 (assembly dependent) and stringMLST version 0.6.3 (assembly independent), all initial ST calls for all selected genomes across all four species (*C. jejuni*, *L. monocytogenes*, *S. aureus*, and *S*. Typhimurium) were done using mlst [10] through the computational platform ProkEvo [28].

### 2.4. Genome-Intrinsic and -Extrinsic Factors That Can Influence Algorithmic Performance

The genome-intrinsic variables considered in these analyses were the number of contigs per genome, total number of nucleotides per genome (genome length), GC% content per genome, and dinucleotide composition of genomes. The number of contigs per genome, as well as the genome length, were calculated using the assembled contigs from SPAdes [36]. The number of contigs was calculated for each genome using the Linux “grep” utility (e.g., grep “>” assembly.fasta | wc -l). The total number of nucleotides per genome was calculated using the “getlengths” function from the AMOS package [38]. For this analysis, we used AMOS v3.1. The “getlengths” function provides the length for each contig, and a custom Bash script was used to summarize these values per genome. The GC% content was calculated using the program FastQC [35]. With each pair of raw reads from all datasets, FastQC v0.11 was used. One of the statistics checked for read quality was GC%, and this value was extracted with a custom Bash script from the file “fastqc_data.txt” once the FastQC output was generated. Since FastQC outputs the GC% per read, the average of both reads was calculated as the final read GC%. The dinucleotide composition of the genomes was calculated with the “compseq” function from the EMBOSS package [39]. For these analyses we used EMBOSS v6.6 with the command “compseq -word 2 -outfile <output> assembly.fasta” for all datasets and genomes assembled a priori with SPAdes [36]. Next, a customized Bash script was used to count the total number of occurrences of each dinucleotide for each genome across all bacterial species. Finally, all these outputs were merged per genome using custom Python script to facilitate statistical analysis and data visualization. The scripts used can be found here: https://github.com/npavlovikj/MLST_stringMLST_analyses/tree/main/scripts (accessed on 20 February 2022).

The genome-extrinsic variables used in the analysis presented in this paper were the total count of unique STs per database and the total count of unique alleles across all seven loci used for ST classification across all bacterial species. These genome-extrinsic variables were extracted from the PubMLST databases for both stringMLST and mlst using custom Bash scripts. For each MLST scheme, the mlst distribution had a separate directory with eight files—seven were “.tfa” files with the fasta sequences of the alleles for each locus, and one file (e.g., senterica.txt) contained the ST information (i.e., the total number of STs mapped, including their specific allelic composition across all seven loci for that given species). To calculate the total number of unique STs, we used the Linux utility “wc” with the text file with ST information (e.g., wc -l senterica.txt). To calculate the total count of unique alleles across the seven loci, the “grep” Linux utility was used with the seven “.tfa” files (e.g., grep “>” *.tfa | wc -l). All calculations were done per bacterial species. The downloaded MLST scheme with stringMLST was in a separate directory for each organism and k-mer length used. This directory had 12 files—seven were “.tfa” files with fasta sequences for all alleles across all seven loci, one file had the ST profiles (e.g., Salmonella_enterica_profile.txt), and the remaining files contained information about the extracted k-mers and additional configure and log information. Similarly, the total number of unique STs for stringMLST was counted using the Linux utility “wc” with the text file with ST profile information (e.g., wc -l Salmonella_enterica_profile.txt), and the total count of unique alleles per loci was extracted using the “grep” Linux utility with the seven “.tfa” files (e.g., grep “>” *.tfa | wc -l). Similarly, all ST and allelic counts were carried out per bacterial species. With stringMLST, the MLST schemes were downloaded and prepared separately for each different k-mer length used. However, the k-mer length did not affect the number of STs and unique alleles per organism.

### 2.5. K-mer-Based Distribution across ST Programs

In order to assess the potential impact of random mapping or the occurrence of k-mers of different lengths across different bacterial species, we randomly chose 100 raw Illumina paired-end reads from the initial *C. jejuni*, *L. monocytogenes*, *S. aureus*, and *S*. Typhimurium (major representative zoonotic serovar of *S. enterica*) isolates. For each read, we extracted all unique k-mers of lengths of 10, 20, 30, 35, 45, 55, 65, 70, 80, and 90, and counted their occurrence in the corresponding raw reads. This was done using DSK v2.2.0 [13] (https://github.com/npavlovikj/MLST_stringMLST_analyses/blob/main/scripts/dsk.submit (accessed on 20 February 2022)). Next, the total number of k-mer frequencies was summarized per organism and k-mer length, and the mean value was calculated to examine the distribution of different k-mers across the raw reads. For each database created with stringMLST, a file with the k-mer frequency for the used ST scheme was generated. Using the k-mers generated from the raw reads and the stringMLST database, a relative frequency of the common k-mers was calculated (calculated as a ratio between the common k-mers and the unique k-mers from all the k-mers generated between the raw reads and the stringMLST database, e.g., (common_k-mers/unique_total_observations) × 100). The code used for this can be found in our GitHub repository (https://github.com/npavlovikj/MLST_stringMLST_analyses/tree/main/figures_code (accessed on 20 February 2022)).

### 2.6. Agreement in ST Classification between Programs

To calculate the percentage of agreement in ST classification, the initial dataset composed of 600 genomes from *C. jejuni*, *L. monocytogenes*, or *S. aureus* was selected, in addition to a total of 11,787 genomes across 20 zoonotic serovars of *S. enterica* (~600 genomes per serovar, Table S1). The program stringMLST was run with increasing k-mer lengths ranging from 10 to 90 nucleotides. If both stringMLST and mlst produced identical ST calls, either “good” or “bad” ones, the call was a match. A “good” and “bad” call represent ST with a number or a missing/blank value, respectively. The remaining combinations were classified as a mismatch. Next, the percentage of agreement (concordance) was calculated with a custom R base script (https://github.com/npavlovikj/MLST_stringMLST_analyses/tree/main/Figures_code (accessed on 20 February 2022)).

### 2.7. Computational Platforms

All computational analyses performed for this paper were done on Crane, one of the high-performance computing clusters at the University of Nebraska–Lincoln Holland Computing Center [40]. The scalability of ProkEvo with stringMLST was tested on the Open Science Grid (OSG) [41,42].

### 2.8. Computational Performance

To access the runtime and memory usage of both programs, we chose four different datasets—*C. jejuni*, *L. monocytogenes*, *S. aureus*, and *S*. Typhimurium (major representative zoonotic serovar of *S. enterica*) with three different batches of 200 genomes each with a total of 600 genomes each. We ran mlst with all required steps, such as quality trimming and adapter clipping, de novo assembly, and assembly discarding on each dataset (see the Software Tools: mlst section for a more detailed description). Separately, we ran stringMLST with a range of 10 different k-mer lengths (10, 20, 30, 35, 45, 55, 65, 70, 80, 90) on each dataset. For each organism, the runtime was calculated as an average of all 200 genomes per batch. The runtime was calculated using the “date” command integrated in the Unix operating systems (e.g., t = ‘date +%s’; mlst --legacy --scheme senterica --csv assembly.fasta > <output>; tt = ‘date +%s’; total_time = $((tt-t))). For each organism, the memory was calculated as the maximum memory recorded from all 200 genomes per batch, since all genomes were analyzed separately and concurrently. In the case of mlst, the recorded memory was the maximum memory of all the steps run prior to mlst, such as trimming, de novo assembly, quality checking, filtering, and ST typing. The memory used was calculated using the “cgget” command that tracks various parameters from the Linux control groups (cgroups) per running job (e.g., mlst --legacy --scheme senterica --csv assembly.fasta > <output>; r = ‘cgget -r memory.usage_in_bytes/slurm/uid_${UID}/job_${SLURM_JOBID}/’; mem = ‘echo $r | awk -F: ‘{print $3}’’).

### 2.9. Incorporating stringMLST in ProkEvo

By following the instructions here (https://github.com/npavlovikj/ProkEvo/wiki/4.1.-Add-new-bioinformatics-tool-to-ProkEvo (accessed on 20 February 2022)), we were able to successfully add stringMLST to the current ProkEvo platform. The ultimate description of how stringMLST was integrated into ProkEvo can be found here: https://github.com/npavlovikj/MLST_stringMLST_analyses/tree/main/Prokevo_stringMLST (accessed on 20 February 2022).

### 2.10. Comparison between mlst and stringMLST Performance Using ProkEvo

In order to compare the performance/accuracy of mlst and stringMLST as part of the ProkEvo platform, two subsets of the *C. jejuni*, *L. monocytogenes*, *S.* Typhimurium, and *S. aureus* datasets used in this paper were selected. One subset was composed of 100 randomly selected genomes, whereas the second one contained 1000. The subsets were randomly selected from the original datasets used in this paper. As part of ProkEvo, stringMLST was run with the default k-mer length of 35. The ProkEvo workflows with mlst and stringMLST and the two datasets were individually run on Crane. Once the four workflows finished, the performance of ProkEvo with mlst and stringMLST and the datasets with 100 and 1000 genomes were compared using (i) the total running time, (ii) the percentage of non-classified STs, and (iii) the percentage of agreement between programs. Since ProkEvo is an automated platform, a list of NCBI-SRA identifications was provided with the ProkEvo implementations with both mlst and stringMLST.

### 2.11. stringMLST-Based k-mer Length Optimization across Phylogenetic Divergent Bacterial Pathogens

To identify the optimal species-specific k-mer length that minimizes the frequency of ST miscalls, we ran stringMLST with a range of different k-mer lengths across phylogenetic divergent pathogenic species. First, we chose 23 *S. enterica* serovars (*S*. Agona, *S*. Anatum, *S*. Braenderup, *S*. Derby, *S*. Dublin, *S*. Enteritidis, *S*. Hadar, *S*. Heidelberg, *S*. Infantis, *S*. Javiana, *S*. Johannesburg, *S*. Kentucky, *S*. Mbandaka, *S*. Montevideo, *S*. Muenchen, *S*. Newport, *S*. Oranienburg, *S*. Poona, *S*. Saintpaul, *S*. Schwarzengrund, *S*. Senftenberg, *S*. Thompson, *S*. Typhimurium), and for each dataset we randomly selected 100 paired-end Illumina reads from NCBI-SRA. Second, for each dataset we ran mlst and stringMLST with k-mer lengths of 20, 30, 35, 40, 45, 18, 55, 60, 65, 70, 80, and 90. The k-mer length of 10 was excluded due to its poor performance in previous analyses. Additionally, we used data from 14 pathogens with Public Health relevance and with available MLST schemes to widen the scope of the analysis and assess the necessity of fine-tuning the k-mer length on a more broadly selected collection of species. In particular, we chose the following pathogens: *Acinetobacter baumannii*, *Clostridioides difficile*, *Enterococcus faecium*, *Escherichia coli*, *Haemophilus influenzae*, *Helicobacter pylori*, *Klebsiella pneumoniae*, *Mycobacterium tuberculosis*, *Neisseria gonorrhoeae*, *Pseudomonas aeruginosa*, *Streptococcus pneumoniae*, *Campylobacter jejuni*, *Listeria monocytogenes*, and *Staphylococcus aureus*. For each pathogen, we randomly selected and downloaded 1000 paired-end reads from NCBI-SRA and processed these reads separately with mlst and stringMLST. stringMLST was run with k-mer lengths of 20, 30, 35, 45, 55, 65, 70, 80, and 90, and different schemes for the different pathogens. Similar to the *S. enterica* datasets, the k-mer length of 10 was excluded from the analysis.

Across all datasets, the percentage of ST miscalls was calculated for stringMLST for each k-mer length, whereby miscalls were defined as “bad” ST calls—calls with missing or blank values. Next, for each dataset, the k-mer length that equated with the lowest percentage of ST miscalls was recorded. For some datasets, multiple k-mer lengths generated an identical lowest percentage for ST miscalls. In this case, we applied a two-folded approach to select the most optimal k-mer length: (1) if a k-mer of length 35 was part of the array of k-mer lengths that showed the most optimal results, we recorded k-mer 35 as the optimal k-mer length since that is the default and recommended value for stringMLST (parsimonious approach), or (2) if a k-mer of length 35 was not part of the k-mer lengths that showed the most optimal results, we recorded the k-mer with the highest value as the most optimal one, since in general our analysis showed that longer k-mers consumed fewer computational resources and sped up the entire analysis. Ultimately, the optimal k-mer length and the percentage of ST miscalls were visualized on a core-genome phylogeny generated for all 23 *S. enterica* serovars, as well as for all 14 pathogens, including all 23 *S. enterica* serovars, which jointly totaled 15 pathogens (a total of 37 genomes—one per species, including one per serovar of *S. enterica*—were used to construct the 16S rRNA-based phylogeny for visualization purposes). The core-genome alignment for the 23 *S. enterica* serovars was generated using Roary with the following set of parameters: “roary -s -e --mafft -p 8 -cd 99 -i 95./prokka_output/*.gff -f roary_output”, and the phylogenetic tree was produced using FastTree [43]. The phylogenetic tree for the 15 pathogens was generated using CLUSTALW (https://www.genome.jp/tools-bin/clustalw, accessed on 20 February 2022) with the 16S rRNA sequences of the selected 37 genomes generated by Prokka [44]. All phylogeny-based visualizations were done using iTOL [45], and the recorded and overlaid statistics were extracted with custom R scripts (https://github.com/npavlovikj/MLST_stringMLST_analyses/blob/main/Figures_code/Figures_code.Rmd, accessed on 20 February 2022).

In addition to calculating the percentage of ST miscalls for different k-mer lengths with stringMLST, for each dataset we calculated the percentage of agreement (concordance) between mlst and stringMLST on ST calls (“good” or “bad”), as previously described here. Of note, when the stringMLST and mlst results were combined, the number of returned ST calls was not always 1000 (the original size of the used datasets). If 1000 reads are used with stringMLST, stringMLST generates ST calls for all 1000 reads. On the other hand, when using mlst, a set of steps are used before mlst, including filtering, and a fraction of assemblies were disregarded due to poor quality. Thus, only genome sequences that passed through the mlst program and yielded a “good” or “bad” call were ultimately used to compare with stringMLST. The number of raw reads for each dataset, as well as the number of final reads from mlst used for these analyses, are shown in Table S4.

### 2.12. Statistical Analyses

To compare the overall performance and accuracy of mlst vs. stringMLST on ST-based classifications, the following statistics were used across all bacterial species datasets: (1) ST richness; (2) Simpson’s D index (1-*D*) of diversity, using ST counts as input data; (3) proportion of non-classified STs (missing values or blank calls); and (4) standard deviation of the proportion of non-classified STs. These statistics were calculated to evaluate the algorithmic performance of ST-based classification accuracy within and between bacterial species selected to be used in the narrow scope analysis (*C. jejuni*, *S. aureus*, *L. monocytogenes*, and *S. enterica*). ST richness was calculated by identifying the number of distinct STs present in each species. The Simpson’s D index of diversity (1-*D*) was used to calculate the degree of genotypic diversity across species, using the “diversity()” function available in the vegan (version 2.5-6) R library [17]. The proportion of non-classified STs was calculated using the counts of isolates or genomes that were not assigned an ST number after each run of either mlst or stringMLST. The standard deviation of the proportion of non-classified STs was calculated using the “sd()” function, which is derived from an unbiased estimate of the sample variance corrected by *n* − 1 (*n* for number of observations). The frequency of genomes used for all analyses was calculated per batch and program across all species, including across serovars for *S. enterica*. The relative frequency of the most dominant ST lineages was also assessed across bacterial species.

PERMANOVA univariate or multivariate models were used to assess the significance (*p* < 0.05) and the degree of association between the genome-intrinsic and -extrinsic factors with the following dependent variables: ST richness, Simpson’s D index of diversity, and proportion of non-classified STs. Statistical models were built for each of the dependent variables separately. Multivariate models included either the combination of bacterial species and program, or serovars, in the case of *S. enterica* and the program. These multivariate models were stated to calculate the main and synergistic effects of the explanatory variables (e.g., species*program or serovar*program). Univariate models were also assessed for each of the dependent variables, using one of the following independent/explanatory variables: (1) genome-intrinsic variables: median number of contigs, mean of the total count of nucleotides per genome, mean of the average GC% content per genome, standard deviation of the number of contigs, standard deviation of the total count of nucleotides per genome, and standard deviation of the average GC% content per genome, or (2) genome-extrinsic variables: species, serovar of *S. enterica*, program (mlst vs. stringMLST with k-mer lengths of 10, 20, 30, 35, 45, 55, 65, 70, 80, and 90), mean of the total count of unique STs per program, mean of the total count of unique alleles across all genes per program, and the Simpson’s D index of diversity per species. Statistical significance and strength of association between the dependent and independent variables were measured with *p*-values (*p* < 0.05) and *R*-squared, respectively. In the case of contig size (median), total number of nucleotides per genome (mean), and GC% content per genome, summary statistic values (median or mean) were calculated and grouped by species and batch (there was a total of three batches per bacterial species or serovar). For the total count of STs and total number of alleles in the database, summary statistic values (mean) were calculated grouped by species, batch, and program. Lastly, the standard deviation of number of contigs, total count of nucleotides per genome, or GC% content per genome were calculated grouped by species. PERMANOVA models were run using the “adonis()” function with 1000 permutations using the vegan (version 2.5-6) R library [17]. Principal component analysis (PCA) was used to analyze the dinucleotide distribution across species and across serovars for *S. enterica* with two dimensions using the “prcomp()” function. The PCA calculations and the selection of the number of PCs were done using the factoextra (version 1.0.7) library. Bar plots, box-and-whiskers plots, and bivariate/trivariate scatter plots were used to assess and visualize the distribution and associations within and between dependent and independent/explanatory variables. R software (version 4.0.3) and R libraries such as Tidyverse (version 1.3.0) were used to conduct all statistical analyses, and all R scripts are available here: https://github.com/npavlovikj/MLST_stringMLST_analyses/tree/main/Figures_code (accessed on 20 February 2022). Data quality control was achieved with R base functions, in addition to the following packages: skimr (version 2.1.3) and visdat (version 0.5.3). Graphical visualizations were achieved using ggplot2 (version 3.3.2), GGally (version 2.1.2), and plotly (version 4.9.4.1). R code integrity was checked using the assertive (version 0.3-6) package.

## 3. Results

**Figure 1:**
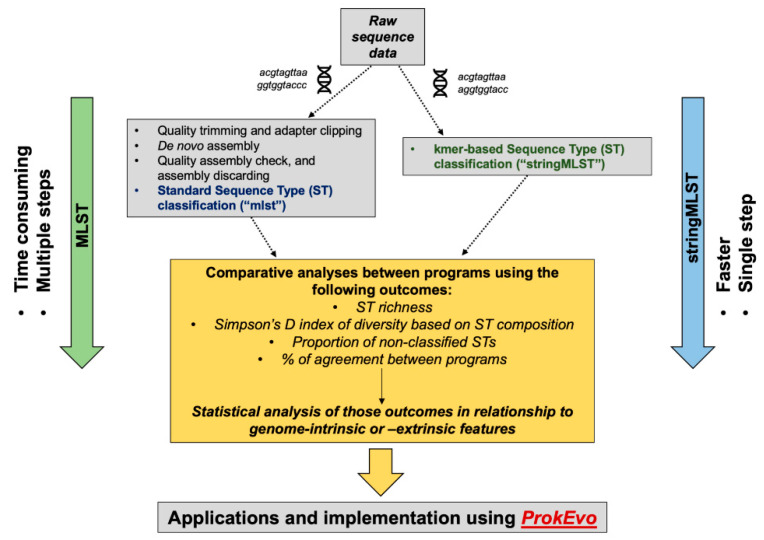
Computational workflow describing the analytical steps for a comparative analysis of two algorithms used for ST-based classification. From top down, the first step (narrow scope) of the analytical approach entailed the acquisition and processing of Illumina paired-end raw reads from four distinct pathogens (*C. jejuni*, *L. monocytogenes*, *S. enterica*, and *S. aureus*) through an assembly-dependent (mlst) or assembly-free (stringMLST) approach for ST-based classification. Next, a set of comparative analyses encompassing measuring the computational performance, statistical metrics, and modeling were used to assess the accuracy and efficiency of mlst vs. stringMLST. Additionally, the contribution of genome-intrinsic and -extrinsic variables were used to identify explanatory factors that could impact the algorithmic efficiency across phylogenetic divergent species. Upon identification of inter-species differences in the performance of stringMLST, a wide-scope analysis was done to assess its accuracy across an array of 15 phylogenetic divergent pathogenic species of bacteria with Public Health relevance. Ultimately, stringMLST was added to the computational platform ProkEvo to facilitate ST-based classification at scale as part of a hierarchical-based approach for population genomic analysis.

The computational and analytical approaches used in this paper are shown in Figure 1. Our analytical approach was subdivided into a narrow- and wide-scope analysis aiming at accomplishing three goals: (1) comparing the computational performance and accuracy of mlst vs. stringMLST, (2) optimizing the use of stringMLST for a wide range of bacterial species, and (3) implementing stringMLST as part of the ProkEvo computational genomics platform.

### 3.1. Computational Performance

**Figure 2:**
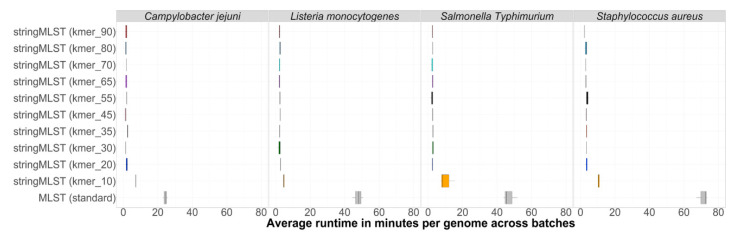
Box-and-whisker plot showing the comparison of the average runtime per genome per batch (in minutes) needed by mlst and stringMLST for ST classification of genomes across four distinct bacterial species. In order to compare the average runtime used by mlst and stringMLST with different k-mer lengths, we chose four different datasets, including four phylogenetic divergent bacterial pathogenic species: *C. jejuni*, *L. monocytogenes*, one major serovar of *S. enterica* (*S*. Typhimurium), and *S. aureus*, using 600 randomly selected genomes for each species. These 600 genomes were randomly split into three batches with 200 genomes each. We then ran mlst with all required steps, such as quality trimming and adapter clipping, de novo assembly, and assembly discarding, on each batch and dataset. Separately, we ran stringMLST with a range of 10 different k-mer lengths (10, 20, 30, 35, 45, 55, 65, 70, 80, 90) on each dataset, including the default length of 35 (*y*-axis). For each organism, the runtime was calculated as an average of 200 genomes per batch—since there were three batches, three datapoints were used to depict the distribution of runtime in minutes (*x*-axis).

The computational performance between stringMLST and mlst was measured using two metrics: (1) the average computational runtime per genome and (2) the maximum memory used per dataset. The average runtime in minutes per genome per batch between mlst and stringMLST with different k-mer lengths for *C. jejuni*, *L. monocytogenes*, *S. aureus*, and *S.* Typhimurium (major representative of *S. enterica*) is shown in Figure 2. Although the runtime of mlst varied between 20 and 80 min per genome depending on the dataset used, all stringMLST runs with different k-mers finished within a few minutes (ranging from ~1 to 16 min when k-mer 10 was included and ~1 to 5 min when k-mer 10 was excluded).

Additionally, a comparison of maximum memory used when both stringMLST and mlst were run for *C. jejuni*, *L. monocytogenes*, *S. aureus*, and *S*. Typhimurium (major representative of *S. enterica*) is shown in Figure S1.

### 3.2. Contribution of Genome-Intrinsic and -Extrinsic Variables

The statistical association with and contribution of each genomic-intrinsic and -extrinsic variable to the accuracy of mlst vs. stringMLST ST calls was assessed on four bacterial species, *C. jejuni*, *S. aureus*, *L. monocytogenes*, and *S. enterica*, using multiple PERMANOVA models. Detailed description of the obtained results is given in Text S1.

### 3.3. Concordance between Programs

**Figure 3:**
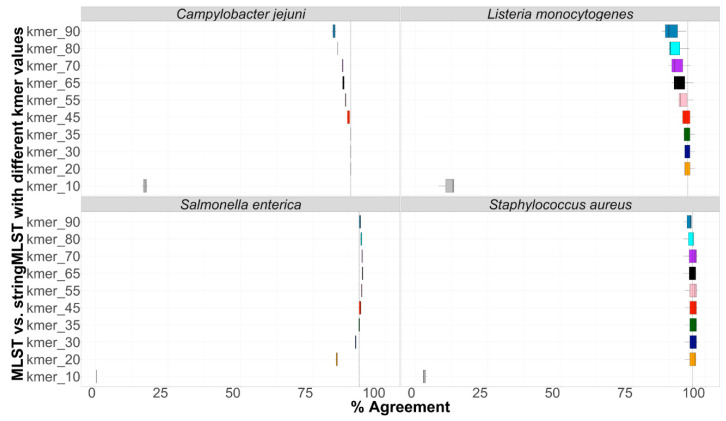
Box-and-whisker plot depicting the concordance between mlst and stringMLST in ST calls. Four different datasets belonging to four phylogenetic distinct bacterial pathogens, including *C. jejuni* (600 genomes), *L. monocytogenes* (600 genomes), *S. enterica* (11,787 genomes from 20 different serovars), and *S. aureus* (600 genomes) were run with mlst and stringMLST for ST-based classification. In the case of stringMLST, k-mer lengths varied from 10 to 90 to identify the optimal value (highest percentage of agreement with the standard MLST approach) across all four species (*y*-axis). If both programs outputted identical ST calls (either number of missing/blank values), the call was defined as a match; otherwise, it was identified as a mismatch, and the percentage of agreement (*x*-axis, concordance) was calculated accordingly. The dashed line on the *x*-axis represents the percentage agreement for the k-mer length of 35, which is used as a default parameter by stringMLST.

Concordance between programs was calculated as the percentage of cases in which outputs from both mlst vs. stringmlst agreed in the call (“good” or “bad”). Results demonstrating the percentage agreement in ST calls between mlst and stringMLST with different k-mer lengths are shown in Figure 3. Apart from k-mer 10, across all species, the percentage of agreement between mlst and stringMLST varied between ~81% and 97%. In the case of *L. monocytogenes*, *C. jejuni*, and *S. aureus*, the k-mer length of 35 appeared to be the optimal value to reach the same accuracy as mlst, which matches the original default and recommended parameter value for stringMLST [30]. However, for *S. enterica*, a higher percentage of agreement with mlst was achieved for k-mer lengths of 55 and 65 (Figure 3).

### 3.4. Optimization of stringMLST k-mer Length across Phylogenetic Divergent Species

**Figure 4:**
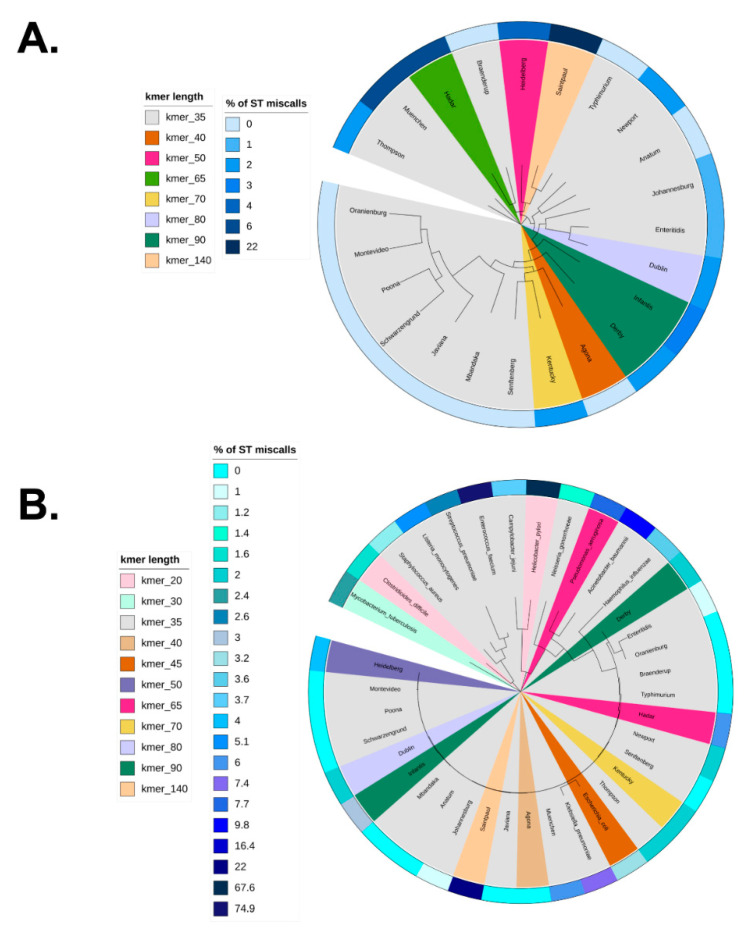
Phylogeny-guided display of optimal k-mer length and algorithmic performance when using stringMLST for ST mapping across bacterial species. (**A**) Core genome-based phylogenetic display of stringMLST results across the 23 zoonotic serovars of *Salmonella enterica* subsp. *enterica* lineage I (*S. enterica*). The branches are colored based on the optimal k-mer length, which gives the lowest percentage of ST miscalls (ST calls that returned missing/blank values for stringMLST). The outer ring present in the phylogeny is colored based on the corresponding ST miscall percentages associated with each optimal k-mer length. The dataset used to identify the optimal k-mer length and percentage of ST miscalls was composed of 2300 genomes (100 genomes per serovar) and the phylogenetic tree was generated using 23 core-genome sequences (one of each serovar to facilitate data visualization). (**B**) Single locus-based (i.e., 16S rRNA gene) phylogenetic display of stringMLST results across 14 divergent bacterial pathogens, including 23 representative genomes across each zoonotic serovar of the *S. enterica* species. The tree branches are colored based on the optimal k-mer length, which minimizes the percentage of ST miscalls (ST calls that returned missing/blank values for stringMLST). The outer ring present in the phylogeny corresponds to ST miscall percentage associated with each optimal k-mer length. The dataset used to identify the optimal k-mer length and percentage of ST miscalls was composed of 14,000 genomes (1000 genomes for each bacterial pathogen) and 2300 *Salmonella* genomes (100 genomes per serovar). The phylogeny (**B**) was generated using the 16S rRNA representative sequences across 37 bacterial species (one for each species was used to facilitate visualization). All phylogeny-based visualizations were generated using iTOL version 6.4.

Figure 4A shows the core-genome phylogeny mapping of the optimized k-mer length across 23 *S. enterica* zoonotic serovars along with their corresponding percentage of ST miscalls when stringMLST was run with wide range of k-mer lengths (20, 30, 35, 40, 45, 50, 55, 60, 65, 70, 80, 90). More detailed information on the distribution of the percentage of ST miscalls for all used k-mer lengths (20, 30, 35, 40, 45, 50, 55, 60, 65, 70, 80, 90) is shown in Figure S16A. As can be seen in Figure 4A, many serovars (*S*. Anatum, *S*. Braenderup, *S*. Javiana, *S*. Mbandaka, *S*. Montevideo, *S*. Oranienburg, *S*. Poona, *S*. Schwarzengrund, *S*. Senftenberg, *S*. Typhimurium) had 0% of miscalls when the default k-mer length 35 was used. *S*. Infantis and *S*. Derby showed the lowest percentage of ST miscalls (3% and 2%, respectively) with a higher value of k-mer, e.g., 90. Interestingly, *S*. Saintpaul showed the highest percentage of ST miscalls when only considering the range of k-mer lengths used for the initial analyses (10–90). To investigate this further, we ran stringMLST for *S*. Saintpaul with k-mer lengths up to 240 (240 was chosen because the maximum read length for the *S*. Saintpaul dataset is 250 base pairs or nucleotides) (Figure S16C,D). As can be seen in Figure S16C, the fewest ST miscalls for *S*. Saintpaul were produced when k-mer of length 140 was used (22%). In addition to the percentage of ST miscalls, we calculated the percentage of ST agreement between mlst and stringMLST with the range of k-mer lengths (Figure S16B). Although for some serovars this percentage was the highest when k-mer when the length 35 was used (e.g., *S*. Anatum, *S*. Braenderup, *S*. Javiana, *S*. Mbandaka, *S*. Montevideo, *S*. Oranienburg, *S*. Poona, *S*. Schwarzengrund, *S*. Senftenberg, *S*. Typhimurium), for other serovars (e.g., *S*. Derby, *S*. Dublin, *S*. Enteritidis, *S*. Hadar, *S*. Heidelberg, *S*. Infantis, *S*. Kentucky, *S*. Saintpaul) the percentage of ST agreement between the two programs was higher with higher k-mer lengths (the percentage of ST agreement varied from ~14 to 100% depending on the serovar and k-mer length used, with 97% being the median across all combinations of serovar and k-mer length).

Figure 4B depicts the 16S rRNA-based phylogeny overlaid with the optimal k-mer length that minimized the percentage of ST miscalls when stringMLST was run with a wide range of k-mer lengths (20, 30, 35, 45, 55, 65, 70, 80, 90) with 14 distinct bacterial pathogens with Public Health relevance. The phylogeny contained 14 distinct pathogens and 23 genomes across each serovar of *S. enterica.* The distribution of the percentage of ST miscalls for all k-mer lengths used (20, 30, 35, 40, 45, 50, 55, 60, 65, 70, 80, 90) is shown in Figure S16E Although the percentage of ST miscalls varied between 0% and 22% across the *S. enterica* serovars, as shown in Figure 4A, the percentage of miscalls was expectedly more variable for the 14 bacterial pathogens, ranging from 1.2% to 74.9%. The datasets for *A. baumannii*, *C. jejuni*, *H. influenzae*, *K. pneumoniae*, *L. monocytogenes*, *N. gonorrhoeae*, *S. aureus*, and *S. pneumoniae* showed the lowest percentage of ST calls with the default k-mer length of 35. *C. difficile* and *M. tuberculosis* had minimized ST miscalls with k-mer lengths of 20 and 30, respectively, whereas *P. aeruginosa* had minimized ST miscalls with a k-mer length of 65. Interestingly, for *E. faecium* and *H. pylori*, the optimal k-mer lengths were 35 and 20, respectively, even though the percentage of miscalls was high (74.9% and 67.6%, respectively). To further investigate this, we ran stringMLST for *E. faecium* and *H. pylori* with k-mer lengths up to 140 (140 was chosen because the maximum read length for the two datasets is 150 base pairs or nucleotides) (Figure S16G,H,K–L). As can be seen in the Figures, the percentage of miscalls was higher with higher k-mer lengths, and the lower k-mer lengths yielded fewer miscalls, albeit this number was still considerably high. Additionally, we ran stringMLST on another set of randomly selected 100 paired-end reads for *E. faecium* (Figure S16M,N), *H. pylori* (Figure S16I,J), and *Enterococcus faecalis* (Figure S16O,P). These 100 reads were not part of the initial datasets and were chosen to validate that the initial random data selection was not completely biased. We also added *E. faecalis* here due to its close phylogenetic association with *E. faecium*. For *E. faecium* and *H. pylori*, we observed the same pattern with 100 reads as with 1000 reads. On the other hand, the pattern for *E. faecalis* was quite opposite, with lowest percentage of ST miscalls of 5.43% for k-mer 35. When comparing the percentage of ST miscalls between mlst and stringMLST, for some datasets, such as *C. jejuni*, *H. pylori*, *L. monocytogenes*, *M. tuberculosis*, *N. gonorrhoeae*, *S. aureus*, mlst performed worse than stringMLST (with an increase in the percentage of miscalls by ~48%, 13%, 11%, 50%, 9%, and 45%, respectively). In addition to the percentage of ST miscalls, we calculated the percentage of ST agreement between mlst and stringMLST with the range of k-mer lengths (Figure S16F). Of note, in the case of stringMLST, when the optimal k-mer length was above the default parameter of 35, the ultimately selected k-mer length was picked based on our empirical evidence for longer k-mers being capable of speeding up the computational analysis.

### 3.5. Incorporating stringMLST in ProkEvo

**Figure 5:**
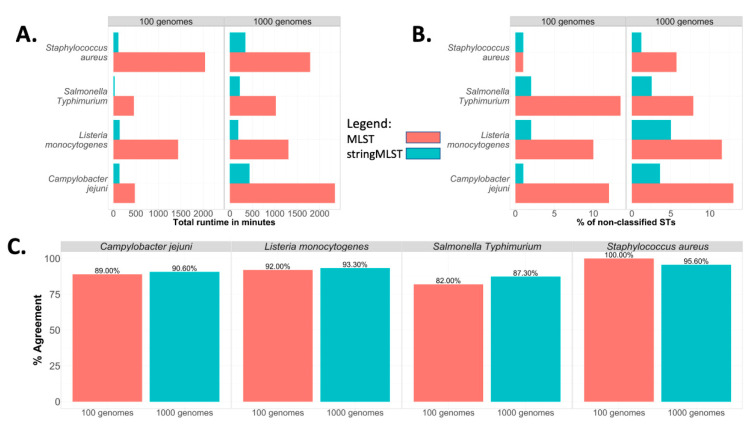
Comparison between the computational and statistical performance of mlst and stringMLST when using ProkEvo to run both programs. Two subsets, one with 100 and the second one with 1000 randomly chosen genomes, were selected from *C. jejuni*, *L. monocytogenes*, one major serovar of *S. enterica* (*S*. Typhimurium), and *S. aureus* to compare the performance of running mlst or stringMLST through ProkEvo. The performance and statistical metrics used for comparison were (**A**) total runtime of individual workflow in minutes, (**B**) percentage of non-classified STs (ST calls that returned missing/blank values), and (**C**) percentage of agreement (concordance) between programs (“good” or “bad” ST calls that matched between mlst and stringMLST).

The workflow design of ProkEvo with both mlst and stringMLST is shown in Figure S17. In order to compare the performance of ProkEvo with mlst and stringMLST, randomly shuffled subsets derived from the original datasets used for *C. jejuni*, *L. monocytogenes*, *S.* Typhimurium, and *S. aureus* were used. One random subset contained 100 genomes, whereas the second one had 1000 genomes. The outcomes measured for this analysis were (i) total running time (Figure 5A), (ii) the percentage of non-classified STs (Figure 5B), and (iii) the percentage of agreement between programs (Figure 5C).

Whereas the runtime of using ProkEvo with mlst varied from ~8 to 34 h for the subset containing 100 genomes, the runtime of ProkEvo with stringMLST varied from ~25 min to 3 h (Figure 5A). Similarly, for the larger datasets containing 1000 genomes, the runtime of ProkEvo with mlst varied from ~17 to 39 h, whereas the runtime of ProkEvo with stringMLST varied from ~4 to 8 h. Regardless of the pathogen species tested, stringMLST sped up the analyses ~4 times when utilizing 1000 genomes across species.

stringMLST resulted in a higher frequency of genomes classified as novel STs (ST numbers that were not classified by mlst) (Figure S18). Additionally, the overall concordance between mlst and stringMLST varied from 82% to 100% across all datasets. The percentage of agreement was the lowest for *S.* Typhimurium, whereas it was the highest for *S. aureus* (Figure 5C).

To further demonstrate the gain in computational runtime obtained with the use of stringMLST within ProkEvo, the complete *S*. Typhimurium dataset containing 23,045 genomes was run on OSG. Whereas ProkEvo with mlst finished all ST calls in 26 days and 6 h when OSG was used as a computational platform [28], ProkEvo with stringMLST completed the task in 3 days and 6 h. Altogether, stringMLST provides a fine-tunable and rapid alternative to MLST for scalable ST genotyping that is portable to be implemented in any high-performance and high-throughput platform, with its use being further facilitated by its implementation in ProkEvo.

## 4. Discussion

This systems-based comparison between mlst and stringMLST was centered on capturing their differences in computational and statistical performances as the methods are distributed by default, and was accomplished through the following steps: (1) narrow-scope comparative analysis across four phylogenetic distinct pathogen species, (2) further examination of algorithmic performance within a single ecologically diverse bacterial species, and (3) wide-scope comparison between phylogenetic divergent pathogenic species with Public Health relevance and with databases available on PubMLST (https://pubmlst.org/, accessed on 20 February 2022) for direct contrast between stringMLST and mlst.

We initially used publicly available raw Illumina paired-end sequence data from bacterial species from two main phylogenetic divergent phyla—Firmicutes (*L. monocytogenes* and *S. aureus*) and Proteobacteria (*S. enterica* and *C. jejuni*)—to run stringMLST and mlst independently in order to compare the accuracy in ST-based classifications and assess the computational needs and performance in the overall analysis (narrow-scope step). For this narrow-scope analysis, we performed a detailed comparative analysis between these two programs, including (i) analyses of computational performance and resources needed (e.g., average runtime per genome and maximum memory needed to analyze all genomes), and (ii) statistical analyses to determine the accuracy of the classifications (e.g., ST richness, Simpson’s D index of ST-based diversity, proportion of miscalls, and percentage of agreement or concordance between programs). For the wide-scope step of the analysis, we systematically evaluated the accuracy and concordance between mlst and stringMLST across a broader array of phylogenetic divergent pathogens with direct implications for Public Health (*A. baumannii*, *C. difficile*, *E. faecium*, *E. coli*, *H. influenzae*, *H. pylori*, *K. pneumoniae*, *M. tuberculosis*, *N. gonorrhoeae*, *P. aeruginosa*, *S. pneumoniae*, *C. jejuni*, *L. monocytogenes*, *S. enterica*, and *S. aureus*). Combined with the intra-species analysis done across 23 serovars of *S. enterica*, our assessment aimed at revealing the optimized k-mer length to be used with stringMLST in order to (i) minimize the percentage of ST miscalls and (ii) minimize the use of computational resources by speeding up the analysis. Lastly, we provided an implementation of stringMLST within ProkEvo.

### 4.1. Computational Platforms

All computational analyses performed for this paper were done on Crane—one of the high-performance computing clusters at the University of Nebraska-Lincoln Holland Computing Center [40]. Crane is a Linux cluster with 548 Intel Xeon nodes with RAM ranging from 64 GB to 1.5 TB. The scalability of ProkEvo with stringMLST was tested on the Open Science Grid (OSG), a distributed, high-throughput computational platform for large-scale scientific research [41,42]. OSG is a national consortium of more than 100 academic institutions and laboratories that provide storage and tens of thousands of resources to OSG users. These sites share their idle resources via OSG for opportunistic usage. The OSG resources are Linux-based, and due to the different sites involved, the hardware specifications of the resources are different and vary.

### 4.2. Computational Performance

Apart from stringMLST with k-mer 10, all other k-mer lengths showed a uniform runtime. The longer runtime observed with k-mer 10 can be partially explained by the higher number of k-mers that were generated and used for mapping (Figure S20). The obtained results show that ST-based classifications are accomplished considerably more rapidly when carried out using stringMLST compared to the standard MLST program.

Across all species, the range of maximum memory usage for mlst and stringMLST (across all k-mers) was ~2–16 GBs and ~3–30 GBs, respectively. Although the memory used across datasets is variable, none of the analyses we ran exceeded 30 GB of RAM. Since most high-performance computers can consistently provide resources from 32 GBs to a few TBs of RAM, the memory available should not be considered a bottleneck for running either program.

### 4.3. Concordance between Programs

As shown in our studies, the accuracy of stringMLST is affected by the species being tested without any specific phylogenetic patterns. In particular, the choice of k-mer length used directly impacts the proportion of ST miscalls across species, and in certain cases it may not be applied as designed even after parameter tuning. It is likely that the varying accuracy reflects the different levels of population structuring, patterns of genome diversification, degree of repetitive sequences, degree of horizontal gene transfer (HGT), and relative abundances of mobile elements such as prophages and insertion sequences. [23,46,47,48,49,50,51,52]. A clear example in our study was *S. enterica*, for which the accuracy of stringMLST at different k-mer lengths varied across ecologically distinct serovars that are known to have unique pan-genomic composition, such as prophage content [23,50,53,54]. Some *S. enterica* serovars showed a decreased proportion of ST miscalls with higher k-mer lengths (Figure S6C). The most likely explanation for the lower accuracy generated by k-mer 10 is that shorter k-mers are more likely to map ambiguously onto a genome compared to other longer lengths. That high frequency of k-mer length 10 on a given dataset reflects their higher likelihood of mapping to multiple regions of a genome (Figures S19A,B and S20).

To the best of our knowledge, the currently available comparisons between ST tools have not considered any systematic approach for parameter tuning across phylogenetic divergent species known to vary in population structure [8,16,28].

### 4.4. Contribution of Genome-Intrinsic and -Extrinsic Variables

In evaluating genome-intrinsic and -extrinsic variables that could contribute to differences in accuracy between mlst and stringMLST, we found that species-level variation in accuracy was mostly explained by the uniqueness of their genomic composition and number of contigs per genome. As genomic composition is an inheritable property of the bacterial species, subtype, and evolutionary history of specific populations, the association with algorithmic performance was somewhat expected [55,56]. However, the contribution of the number of contigs to performance implies that data from long-read sequencing platforms such as PacBio and Oxford Nanopore Technologies (ONT) may have a considerable effect on the accuracy of programs such as stringMLST because these technologies produce reads with lower accuracy (~80–90%) that may inflate the number of false allelic calls at MLST loci, with the effect of artificially splitting major STs into multiple sub-populations [57,58,59]. Therefore, although more work is needed in this field, current studies using hybrid assembly approaches of both Illumina short reads and ONT long reads [60], as well as only polished ONT reads [61] for performing ST-based classification, may lead to promising, cost-effective results from these types of platforms. Hence, we expect that a combination of hybrid sequencing strategies with species and even subtype-specific tuning of programs such as stringMLST and STing will facilitate real-time surveillance, the prediction of STs, and the prediction of traits such as AMR that are found to be associated with specific STs [25,62]. Although ST databases can differ while comparing programs, we anticipate that stringMLST accuracy will only get better in that regard by the increasing size of databases (i.e., allelic composition across loci) and curation.

### 4.5. Optimization of stringMLST k-mer Length across Phylogenetic Divergent Species

When comparing the percentage of ST miscalls between mlst and stringMLST, in general, mlst performed better than stringMLST for the used datasets and range of k-mer lengths (the percentage of miscalls for mlst ranged from ~0 to 1% across all serovars, whereas the percentage of miscalls for stringMLST ranged from ~0 to 85% depending on the serovar and k-mer length used, with 2% being the median across all combinations of serovar and k-mer length for stringMLST).

Although the default k-mer length of 35 used by stringMLST performs accurately across many organisms, our systems-based approach encompassing the analysis of a broad array of phylogenetic divergent organisms revealed (i) that intra- and inter-species variation in the percentage of ST miscalls requires fine-tuning of the k-mer length parameter, (ii) a lack of association between taxonomy or the phylogenetic placement of organisms and the optimal k-mer length, and (iii) that unique species behave as outliers for which stringMLST cannot be directly applied with the default settings and may hinder epidemiological investigations. Across phylogenetic divergent pathogenic bacterial species, the optimal k-mer length ranged from 20 to 140, regardless of their ancestral relationship or speciation pattern. The varying population structure, the pattern of genome diversification and architecture (e.g., impact of HGT), and sequence coverage may be some of the reasons underlying the observed statistics [23,46,47,48,49,50,51,52]. Although we hypothesize that longer sequence reads will help overcome this limitation, there is still a context-dependent consideration for parameter tuning and overall algorithmic implementation.

Therefore, in the case of stringMLST, we suggest the following actionable strategies to maximize its utilization, including (i) developers considering implementing a pre-step that heuristically searches for the optimal k-mer length (minimizes ST miscalls) in a dataset-dependent fashion (strategically sampling from the testing data while considering its population structure–ST distribution), perhaps even by comparing with the standard MLST program as positive control in an iterative fashion from time to time to optimize its parameters, and/or (ii) researchers running a wide range of k-mer lengths on a subset of the dataset in order to select the optimal k-mer length that minimizes the percentage of ST miscalls. Given the speed and scalability of stringMLST, using multiple k-mer lengths is not likely to add much overhead to the analyses, and this provides an empirical statistical approach for k-mer selection and the optimization of ST classifications. With this data-driven fine-tuning of the k-mer length, stringMLST is a powerful program that can be efficiently and effectively used in microbiological and epidemiological laboratories.

### 4.6. Incorporating stringMLST in ProkEvo

As part of this work, we modified ProkEvo to not only offer the standard assembly-dependent MLST mapping approach, but also to contain stringMLST, and our tests showed a significant speed-up in runtime for datasets ranging from a few hundreds to tens of thousands of genomes.

Our experiments showed that in terms of accuracy in ST classifications, the use of stringMLST considerably decreased the number of non-classified STs, regardless of the dataset size (100 or 1000 genomes) and bacterial species (Figure 5B). Moreover, the lower proportion of ST miscalls and high percentage of agreement between programs for *S. aureus*, compared to other species, is associated with its higher degree of genetic homogeneity (fewer dominant STs) (Figure S4). This difference in miscalls and concordance between programs may be further explained by the variation in ST database sizes, since the PubMLST schemes used for mlst have fewer alleles across all seven loci, which results in fewer STs compared to stringMLST, as shown in Table S3. For the analyses in this paper, both mlst and stringMLST were applied with their default distributions and PubMLST schemes, which differ.

We additionally added STing to ProkEvo as an efficient successor of stringMLST (https://github.com/npavlovikj/MLST_stringMLST_analyses/tree/main/Prokevo_STing (accessed on 20 February 2022)). The Pegasus Workflow Managements System that is used by ProkEvo automatically handles the dependencies, as well as all the intermediate and final files. Thus, using platforms such as ProkEvo with fast tools for hierarchical genotyping such as stringMLST allows for robust and efficient population-based genomics analyses that facilitates (i) mapping and tracking variants or lineages for epidemiological inquiries, (ii) population structure analysis, and (iii) ecological trait prediction using pan-genomic mapping to specific genotypes.

## 5. Conclusions

In conclusion, stringMLST largely proved to be an accurate, rapid, and scalable tool for ST-based classifications that could be deployed in microbiological laboratories and epidemiological agencies. However, our work clearly illustrates the need to optimize stringMLST across phylogenetic divergent species and populations of bacterial pathogens. Based on our results, we propose that the k-mer length should be optimized in two ways on a case-by-case basis: (1) intrinsically, by implementing a pre-step inside the algorithm to sample from the target data and select the optimal k-mer length, or (2) by the user, through a heuristic data-mining approach to select the optimal k-mer length prior to finalizing the ST calls. In addition, by assessing genome-intrinsic and -extrinsic factors that could affect the stringMLST performance, our work suggests that longer sequence reads have the potential to improve its accuracy for specific bacterial species. Furthermore, the integration of stringMLST into ProkEvo allows users to take advantage of other hierarchical genotyping strategies, including pan-genomic mapping, which reproducibly facilitates ecological and epidemiological inquiries at scale. Ultimately, this work emphasizes the importance of developing robust algorithmic tools for mining WGS data that can have direct implications for mapping and tracking bacterial populations.

## Data Availability

Not applicable.

## Supplementary Materials

The following supporting information can be downloaded at: https://www.mdpi.com/article/10.3390/life12050670/s1.
