# Supplementary material for: Systems-Based Approach for Optimization of Assembly-Free Bacterial MLST Mapping
Source: Life (Basel). 2022 Apr 30;12(5):670. doi: 10.3390/life12050670 (PMC9147691; doi:10.3390/life12050670)
Supplement: Supplementary file 1 [file life-12-00670-s001.zip › Figure S1.pdf]

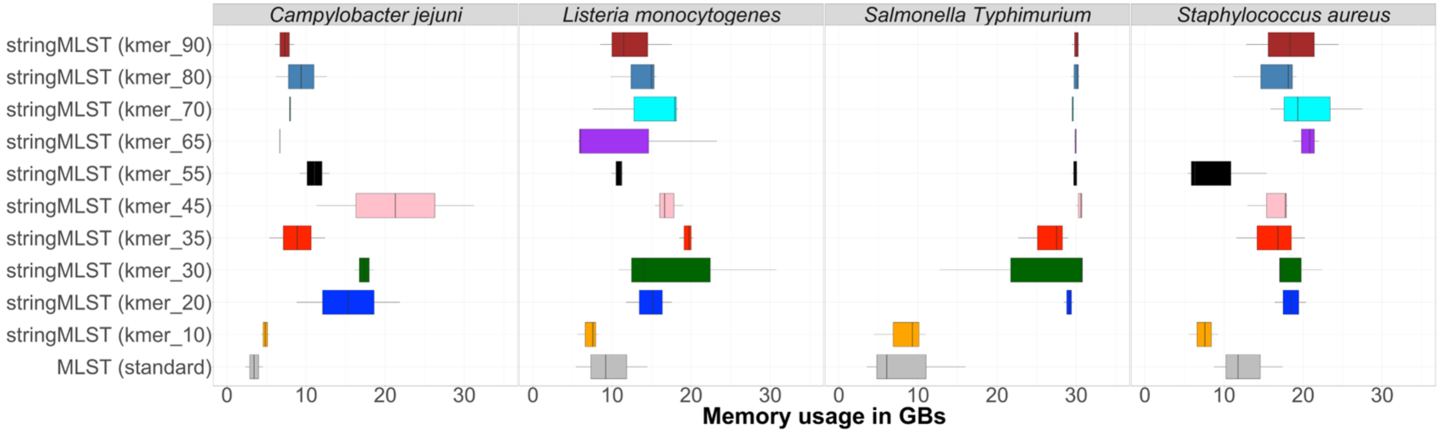

Figure S1. Comparison of the maximum computational memory used in GBs by mlst and stringMLST for ST-based classification of genomes across four bacterial species.

In order to compare the memory used between mlst and stringMLST with different k-mer lengths, we chose four different datasets, including four phylogenetic divergent bacterial species (pathogens): *C. jejuni*, *L. monocytogenes*, one major serovar of *S. enterica* (*S*. Typhimurium) and *S. aureus*, with 600 genomes each. These randomly selected 600 genomes were randomly split into three batches with 200 genomes each. We then ran mlst with all required steps, such as quality trimming and adapter clipping, *de novo* assembly and assembly discarding, on each batch and dataset (three datapoints per bacterial species). Separately, we ran stringMLST with a range of 10 different k-mer values (10, 20, 30, 35, 45, 55, 65, 70, 80, 90) on each dataset. For each organism, the memory was calculated as the maximum of all 200 genomes per batch. In the case of mlst, the recorded memory was the maximum memory of all the steps ran prior to mlst, such as trimming, *de novo* assembly, quality checking, filtering, and ST typing. The memory was calculated using the "cgget" command (*cgget -r memory.usage_in_bytes/slurm/uid_${UID}/job_${SLURM_JOBID}/*) part of the Linux Control Groups (cgroup).
