# Supplementary material for: Systems-Based Approach for Optimization of Assembly-Free Bacterial MLST Mapping
Source: Life (Basel). 2022 Apr 30;12(5):670. doi: 10.3390/life12050670 (PMC9147691; doi:10.3390/life12050670)
Supplement: Supplementary file 1 [file life-12-00670-s001.zip › Figure S10.pdf]

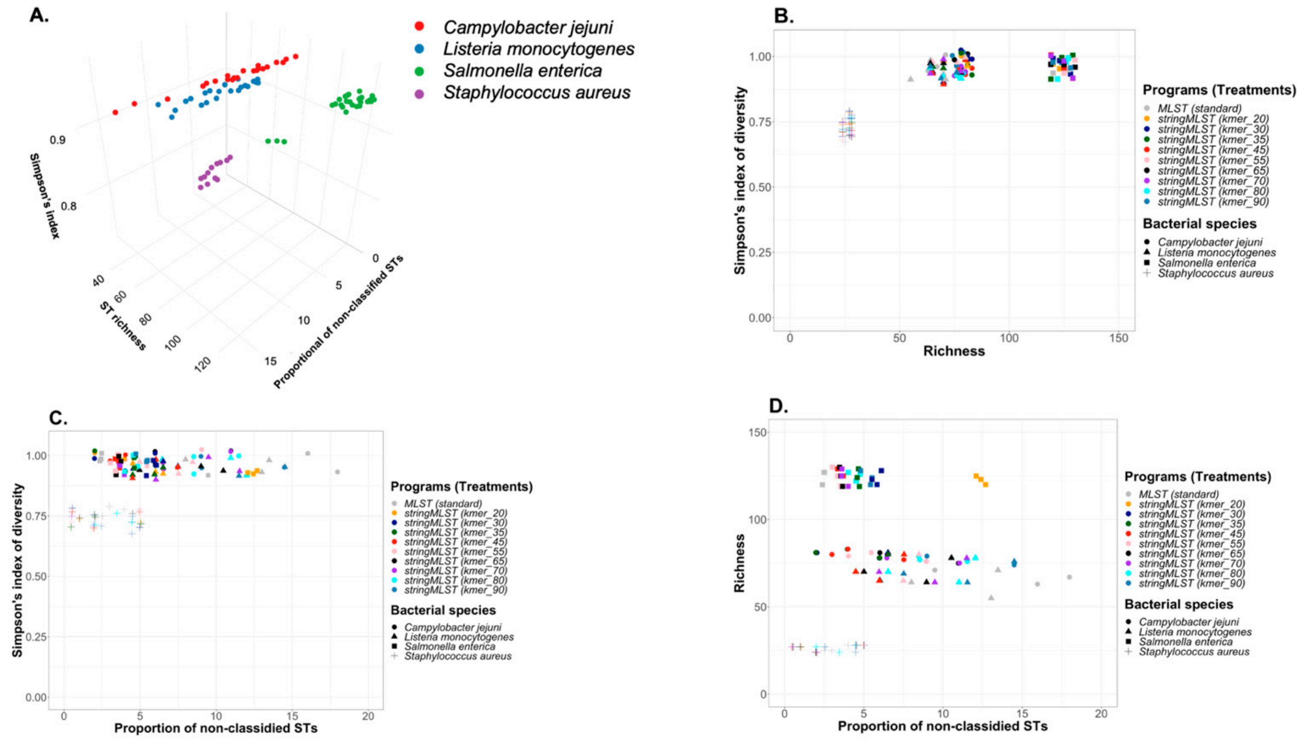

Figure S10. Multi-dimensional analysis of ST-based classification outcomes across different species using mlst vs. stringMLST.

(A) Tri-dimensional scatter plot demonstrating species grouping based on the outcomes calculated using the ST classification across programs, including: 1) Simpson's index of diversity (1 – *D,* Simpson's index); 2) ST richness; and 3) proportion of non-classified STs. (B-D) Biplots demonstrating groupings formed across species and programs based on the same outcomes. Scatter plot B depicts groupings produced based on the relationship between Simpson's index of diversity vs. ST richness (Richness); whereas scatter plot C shows the relationship between the Simpson's index of diversity and the proportion of non-classified STs; and lastly, scatter plot D depicts the relationship between ST richness (Richness) and the proportion of non-classified STs.
