# Supplementary material for: Systems-Based Approach for Optimization of Assembly-Free Bacterial MLST Mapping
Source: Life (Basel). 2022 Apr 30;12(5):670. doi: 10.3390/life12050670 (PMC9147691; doi:10.3390/life12050670)
Supplement: Supplementary file 1 [file life-12-00670-s001.zip › Figure S11.pdf]

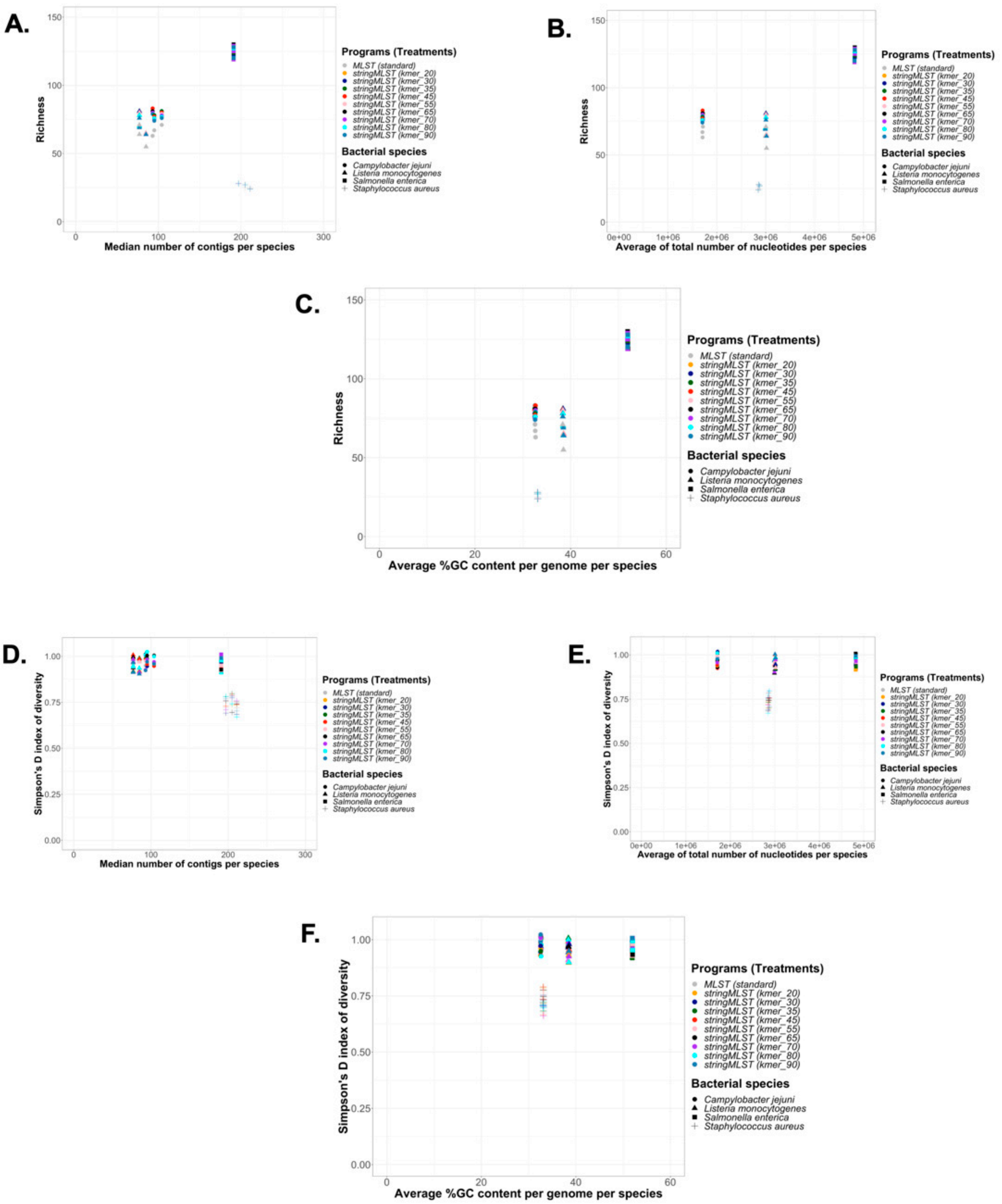
A.
Richness
Median number of contigs per species
B.
Average of total number of nucleotides per species
C.
Average %GC content per genome per species
D.
Simpson's D index of diversity
E.
F.
Programs (Treatments)
MLST (standard)
stringMLST (kmer_20)
stringMLST (kmer_30)
stringMLST (kmer_35)
stringMLST (kmer_45)
stringMLST (kmer_55)
stringMLST (kmer_65)
stringMLST (kmer_70)
stringMLST (kmer_80)
stringMLST (kmer_90)
Bacterial species
Campylobacter jejuni
Listeria monocytogenes
Salmonella enterica
Staphylococcus aureus

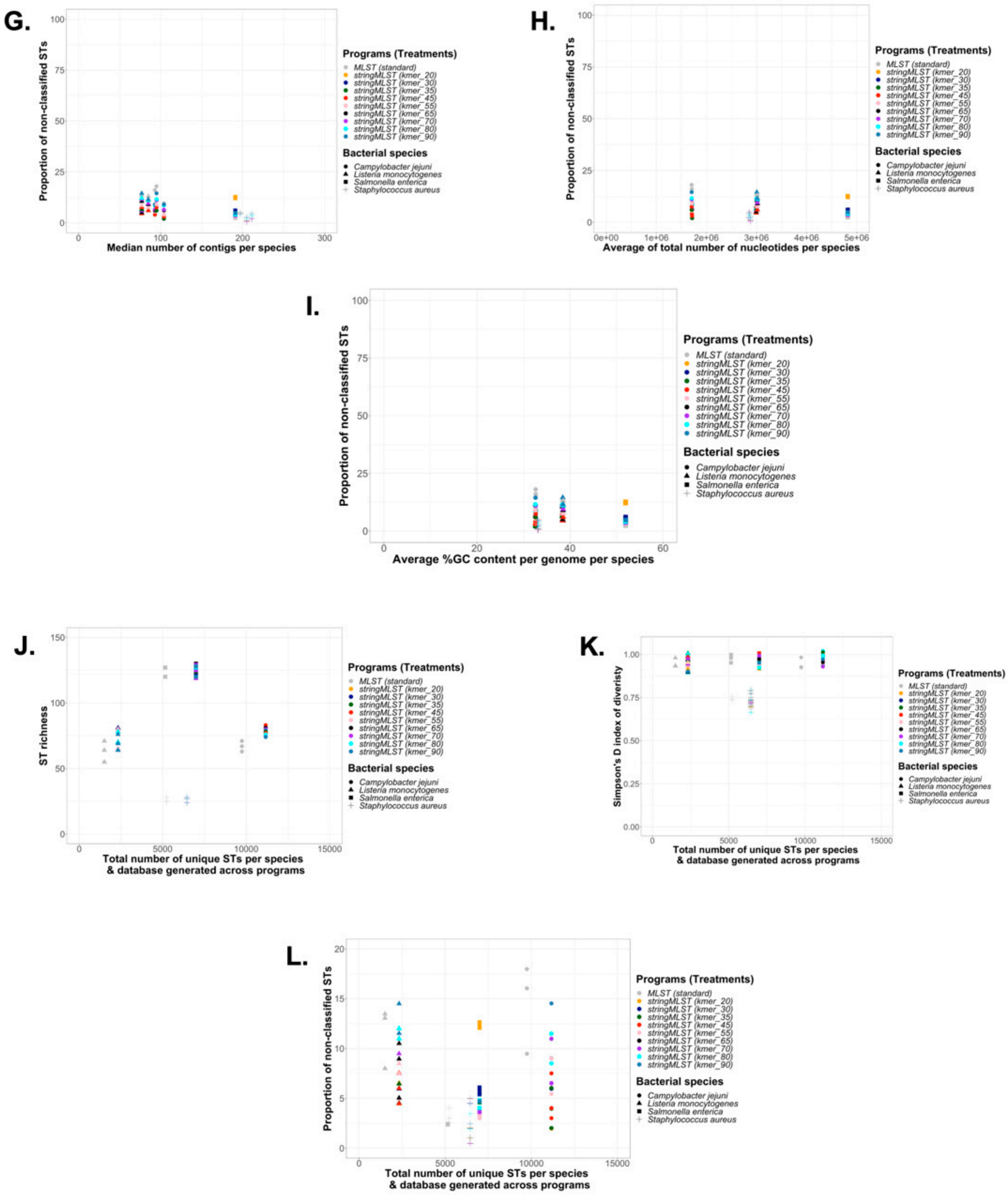

G.
Proportion of non-classified STs
Median number of contigs per species
Programs (Treatments)
MLST (standard)
stringMLST (kmer_20)
stringMLST (kmer_30)
stringMLST (kmer_35)
stringMLST (kmer_45)
stringMLST (kmer_55)
stringMLST (kmer_65)
stringMLST (kmer_70)
stringMLST (kmer_80)
stringMLST (kmer_90)
Bacterial species
Campylobacter jejuni
Listeria monocytogenes
Salmonella enterica
Staphylococcus aureus
H.
Proportion of non-classified STs
Average of total number of nucleotides per species
I.
Proportion of non-classified STs
Average %GC content per genome per species
J.
ST richness
Total number of unique STs per species & database generated across programs
K.
Simpson's D index of diveristy
Total number of unique STs per species & database generated across programs
L.
Proportion of non-classified STs
Total number of unique STs per species & database generated across programs

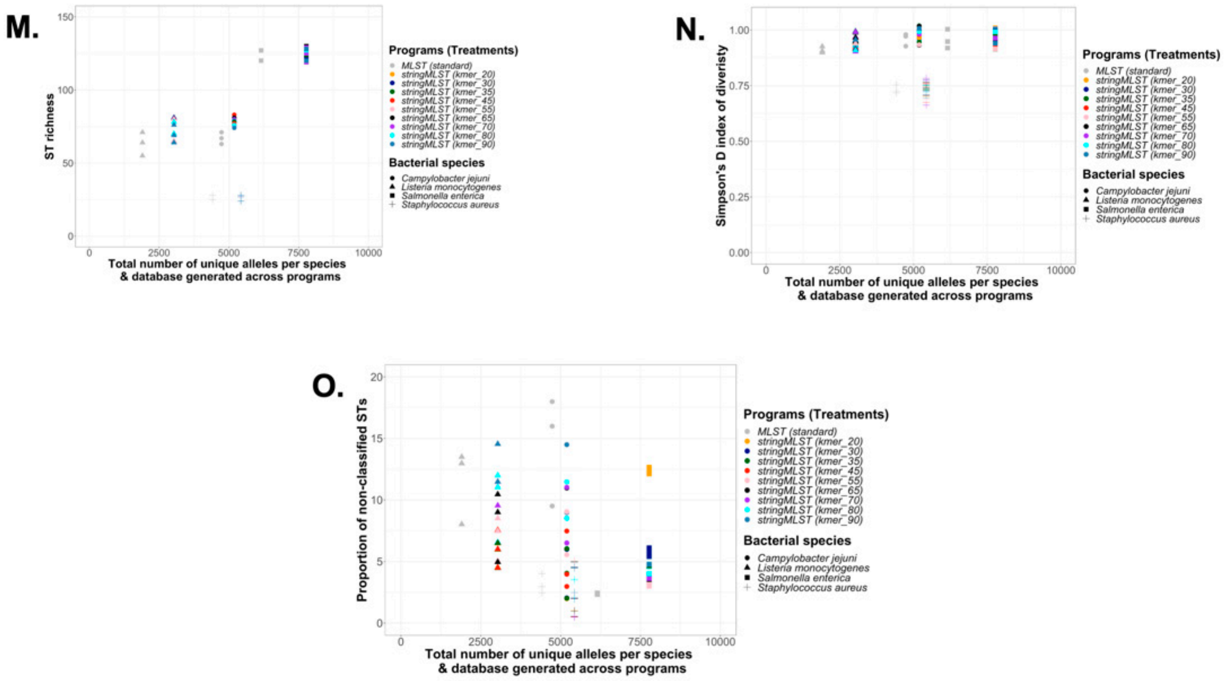

Figure S11. Bivariate association between accuracy-based classification metrics for ST calls and genome-intrinsic and –extrinsic variables.
Plot A-C depict groupings generated based on the relationship between ST richness (Richness) vs. the median number of contigs per species, or the average of total number of nucleotides per species, or the average GC% content per genome per species, respectively. Plot D-F depict groupings generated based on the relationship between the Simpson's D index of diversity vs. the median number of contigs per species, or the average of total number of nucleotides per species, or the average GC% content per genome per species, respectively. Plot G-I depict groupings generated based on the relationship between the proportion of non-classified STs vs. the median number of contigs per species, or the average of total number of nucleotides per species, or the average GC% content per genome per species, respectively. Plot J-L depict groupings generated based on the relationship between the total number of unique STs per species (generated across databases per program) vs. ST richness, or the Simpson's D index of diversity, or the proportion of non-classified STs, respectively. Plot M-O depict groupings generated based on the relationship between the total number of unique alleles per species (per database generated across programs) vs. ST richness, or the Simpson's D index of diversity, or the proportion of non-classified STs, respectively.
