# Supplementary material for: Systems-Based Approach for Optimization of Assembly-Free Bacterial MLST Mapping
Source: Life (Basel). 2022 Apr 30;12(5):670. doi: 10.3390/life12050670 (PMC9147691; doi:10.3390/life12050670)
Supplement: Supplementary file 1 [file life-12-00670-s001.zip › Figure S12.pdf]

A.

PERMANOVA model using ST richness as *dependent variable* and the following independent variables:
***S. enterica* serovars (serovar) and program as MLST or stringMLST (program)**

Model = adonis(formula = n ~ serovar * program, data = d14, permutations = 1000)

	Df	SumsOfSqs	MeanSqs	F.Model	R^2	Pr(>F)
**serovar**	19	39.5743056229991	2.08285819068416	65.7453972769742	0.753954496075907	0.000999000999000999
**program**	9	0.0302818094505213	0.00336464549450236	0.106205000283566	0.000576917422178744	1
**serovar:program**	171	0.212129026087382	0.00124052062039405	0.0391570205705148	0.00404140086475292	1
**Residuals**	400	12.6722677294621	0.0316806693236552		0.241427185637162	
**Total**	599	52.4889841879991			1	

B.

PERMANOVA model using ST richness as *dependent variable* and the following independent variable:
***S. enterica* serovars (serovar)**

Model = adonis(formula = n ~ serovar, data = d14, permutations = 1000)

	Df	SumsOfSqs	MeanSqs	F.Model	R^2	Pr(>F)
**serovar**	19	39.5743056229991	2.08285819068416	93.5414493296616	0.753954496075907	0.000999000999000999
**Residuals**	580	12.914678565	0.0222666871810345		0.246045503924093	
**Total**	599	52.4889841879991			1	

C.

PERMANOVA model using ST richness as *dependent variable* and the following independent variable: program as MLST or stringMLST (program)

Model = adonis(formula = n ~ program, data = d14, permutations = 1000)

	Df	SumsOfSqs	MeanSqs	F.Model	R^2	Pr(>F)
**program**	9	0.0302818094505365	0.00336464549450406	0.0378419738146088	0.000576917422179034	1
**Residuals**	590	52.4587023785486	0.0889130548788959		0.999423082577821	
**Total**	599	52.4889841879991			1	

D.

PERMANOVA model using ST richness as *dependent variable* and the following independent variable: median of the number of contigs (num_contigs_median)

Model = adonis(formula = n ~ num_contigs_median, data = d14, permutations = 1000)

	Df	SumsOfSqs	MeanSqs	F.Model	R^2	Pr(>F)
**num_contigs_median**	1	0.44768112521022	0.44768112521022	5.1442469177359	0.00852904913546748	0.011988011988012
**Residuals**	598	52.0413030627889	0.0870255904060015		0.991470950864533	
**Total**	599	52.4889841879991			1	

E.

PERMANOVA model using ST richness as *dependent variable* and the following independent variable: mean of the total counts for nucleotides per genomes (total_nucl_mean)

Model = adonis(formula = n ~ total_nucl_mean, data = d14, permutations = 1000)

	Df	SumsOfSqs	MeanSqs	F.Model	R^2	Pr(>F)
**total_nucl_mean**	1	0.238098824297164	0.238098824297164	2.72498917364979	0.00453616750220138	0.0729270729270729
**Residuals**	598	52.2508853637019	0.0873760624811069		0.995463832497799	
**Total**	599	52.4889841879991			1	

F.

PERMANOVA model using ST richness as *dependent variable* and the following independent variable: mean of the average GC% per genome (gc_avg_mean)

Model = adonis(formula = n ~ gc_avg_mean, data = d14, permutations = 1000)

	Df	SumsOfSqs	MeanSqs	F.Model	R^2	Pr(>F)
**gc_avg_mean**	1	2.54799906279066	2.54799906279066	30.5100797617166	0.0485435011213881	0.000999000999000999
**Residuals**	598	49.9409851252084	0.083513353052188		0.951456498878612	
**Total**	599	52.4889841879991			1	

G.

PERMANOVA model using ST richness as *dependent variable* and the following independent variable: mean of the total counts of unique STs per program (st_count_mean)

Model = adonis(formula = n ~ st_count_mean, data = d14, permutations = 1000)

	Df	SumsOfSqs	MeanSqs	F.Model	R^2	Pr(>F)
**st_count_mean**	1	0.00089060831664228	0.00089060831664228	0.0101467539975245	1.69675281474757E-05	0.996003996003996
**Residuals**	598	52.4880935796825	0.0877727317385995		0.999983032471852	
**Total**	599	52.4889841879991			1	

H.

PERMANOVA model using ST richness as *dependent variable* and the following independent variable: mean of the total counts of unique alleles across all genes per program (total_alleles_genes_mean)

Model = adonis(formula = n ~ total_alleles_genes_mean, data = d14, permutations = 1000)

	Df	SumsOfSqs	MeanSqs	F.Model	R^2	Pr(>F)
**total_alleles_genes_mean**	1	0.000890608316637105	0.000890608316637105	0.0101467539974656	1.69675281473771E-05	0.996003996003996
**Residuals**	598	52.4880935796825	0.0877727317385994		0.999983032471853	
**Total**	599	52.4889841879991			1	

I.

PERMANOVA model using ST richness as *dependent variable* and the following independent variable: Simpson's D index of diversity per *S. enterica* serovar (simpson)

Model = adonis(formula = n ~ simpson, data = d14, permutations = 1000)

	Df	SumsOfSqs	MeanSqs	F.Model	R^2	Pr(>F)
**simpson**	1	24.4898921577139	24.4898921577139	523.051086602101	0.466572034810176	0.000999000999000999
**Residuals**	598	27.9990920302852	0.0468212241309117		0.533427965189824	
**Total**	599	52.4889841879991			1	

J.

PERMANOVA model using ST richness as *dependent variable* and the following independent variable: Standard deviation of the number of contigs (num_contigs_sd)

Model = adonis(formula = n ~ num_contigs_sd, data = d16, permutations = 1000)

	Df	SumsOfSqs	MeanSqs	F.Model	R^2	Pr(>F)
**num_contigs_sd**	1	2.36289028502892	2.36289028502892	28.1890783906377	0.0450168796668989	0.000999000999000999
**Residuals**	598	50.1260939029702	0.0838228995032946		0.954983120333101	
**Total**	599	52.4889841879991			1	

K.

PERMANOVA model using ST richness as *dependent variable* and the following independent variable:
Standard deviation of the total counts for nucleotides per genomes (total_nucl_sd)

Model = adonis(formula = n ~ total_nucl_sd, data = d16, permutations = 1000)

	Df	SumsOfSqs	MeanSqs	F.Model	R^2	Pr(>F)
**total_nucl_sd**	1	1.704263803706	1.704263803706	20.0680391051517	0.0324689804931615	0.000999000999000999
**Residuals**	598	50.7847203842931	0.0849242815790854		0.967531019506839	
**Total**	599	52.4889841879991			1	

L.

PERMANOVA model using ST richness as *dependent variable* and the following independent variable:
Standard deviation of the average GC% per genome (gc_avg_sd)

Model = adonis(formula = n ~ gc_avg_sd, data = d16, permutations = 1000)

	Df	SumsOfSqs	MeanSqs	F.Model	R^2	Pr(>F)
**gc_avg_sd**	1	0.884904844087155	0.884904844087155	10.2544818838348	0.0168588677753355	0.002997002997003
**Residuals**	598	51.6040793439119	0.0862944470633979		0.983141132224664	
**Total**	599	52.4889841879991			1	

Figure S12. PERMANOVA results measuring the association between *S. enterica* serovars (serovar), program, or genome-intrinsic and –extrinsic variables and ST richness.
PERMANOVA results demonstrating the association (*R*-squared and *p*-values) between ST richness (n) and: (A) serovar and program (mlst vs. stringMLST with all k-mer lengths); (B) serovar; (C) program (mlst vs. stringMLST with all k-mer

lengths); (D) the median number of contigs (num_contigs_median); (E) the mean total number of nucleotides (total_nucl_mean); (F) the mean GC% content originally calculated per genome (gc_avg_mean); (G) the mean total count of STs present in each generated database (st_count_mean); (H) the mean total count of unique alleles (across all 7 loci) present in each generated database (total_alleles_genes_mean); (I) the Simpson's D index of diversity (simpson); (J) the standard deviation (SD) of the number of contigs (num_contigs_sd); (K) the SD of the total number of nucleotides (total_nucl_sd); (L) the SD of the GC% content per genome (gc_avg_sd). The median number of contigs, mean total number of nucleotides, and mean GC% content were grouped by serovar and batch (experimental replicate). The SD of the number of contigs, SD of the total number of nucleotides, and SD of GC% content were calculated by serovar only. The mean total count of STs and mean total count of unique alleles (across all 7 loci) present in each generated database were calculated after grouping by serovar, batch (three experimental replicates), and program. The Simpson's D index of diversity was calculated after grouping by program, serovar, and batch (three experimental replicates). All PERMANOVA models were run with 1,000 permutations.
