# Supplementary material for: Systems-Based Approach for Optimization of Assembly-Free Bacterial MLST Mapping
Source: Life (Basel). 2022 Apr 30;12(5):670. doi: 10.3390/life12050670 (PMC9147691; doi:10.3390/life12050670)
Supplement: Supplementary file 1 [file life-12-00670-s001.zip › Figure S13.pdf]

A.

PERMANOVA model using the Simpson's D index of diversity as *dependent variable* and the following independent variables:
***S. enterica* serovars (serovar) and program as MLST or stringMLST (program)**

Model = adonis(formula = simpson ~ serovar * program, data = d14, permutations = 1000)

	Df	SumsOfSqs	MeanSqs	F.Model	R^2	Pr(>F)
**serovar**	17	85.7297109948326	5.04292417616662	194.682446036456	0.880851447565824	0.000999000999000999
**program**	9	0.192616168185751	0.0214017964650834	0.826217872774765	0.00197908319767026	0.659340659340659
**serovar:program**	153	2.07843106001404	0.0135845167321179	0.524431233396458	0.0213553619466825	1
**Residuals**	360	9.32519978241913	0.025903332728942		0.0958141072898229	
**Total**	539	97.3259580054515			1	

B.

PERMANOVA model using the Simpson's D index of diversity as *dependent variable* and the following independent variable:
***S. enterica* serovars (serovar)**

Model = adonis(formula = simpson ~ serovar, data = d14, permutations = 1000)

	Df	SumsOfSqs	MeanSqs	F.Model	R^2	Pr(>F)
**serovar**	17	85.7297109948326	5.04292417616662	227.005031675199	0.880851447565824	0.000999000999000999
**Residuals**	522	11.5962470106189	0.0222150325873926		0.119148552434176	
**Total**	539	97.3259580054515			1	

C.

PERMANOVA model using the Simpson's D index of diversity as *dependent variable* and the following independent variable: program as MLST or stringMLST (program)

Model = adonis(formula = simpson ~ program, data = d14, permutations = 1000)

	Df	SumsOfSqs	MeanSqs	F.Model	R^2	Pr(>F)
**program**	9	0.192616168185761	0.0214017964650846	0.116777122169836	0.00197908319767037	1
**Residuals**	530	97.1333418372657	0.183270456296728		0.99802091680233	
**Total**	539	97.3259580054515			1	

D.

PERMANOVA model using the Simpson's D index of diversity as *dependent variable* and the following independent variable: median of the number of contigs (num_contigs_median)

Model = adonis(formula = simpson ~ num_contigs_median, data = d14, permutations = 1000)

	Df	SumsOfSqs	MeanSqs	F.Model	R^2	Pr(>F)
**num_contigs_median**	1	1.06238403124153	1.06238403124153	5.93747546669179	0.0109157315582963	0.003996003996004
**Residuals**	538	96.26357397421	0.178928576160242		0.989084268441704	
**Total**	539	97.3259580054515			1	

E.

PERMANOVA model using the Simpson's D index of diversity as *dependent variable* and the following independent variable: mean of the total counts for nucleotides per genomes (total_nucl_mean)

Model = adonis(formula = simpson ~ total_nucl_mean, data = d14, permutations = 1000)

	Df	SumsOfSqs	MeanSqs	F.Model	R^2	Pr(>F)
**total_nucl_mean**	1	0.70701945670127	0.70701945670127	3.93687276447733	0.00726444898350415	0.01998001998002
**Residuals**	538	96.6189385487502	0.179589105109201		0.992735551016496	
**Total**	539	97.3259580054515			1	

F.

PERMANOVA model using the Simpson's D index of diversity as *dependent variable* and the following independent variable: mean of the average GC% per genome (gc_avg_mean)

Model = adonis(formula = simpson ~ gc_avg_mean, data = d14, permutations = 1000)

	Df	SumsOfSqs	MeanSqs	F.Model	R^2	Pr(>F)
**gc_avg_mean**	1	7.47341510145232	7.47341510145232	44.7477299432379	0.0767874804893646	0.000999000999000999
**Residuals**	538	89.8525429039992	0.167012161531597		0.923212519510635	
**Total**	539	97.3259580054515			1	

G.

PERMANOVA model using the Simpson's D index of diversity as *dependent variable* and the following independent variable:
mean of the total counts of unique STs per program (st_count_mean)

Model = adonis(formula = simpson ~ st_count_mean, data = d14, permutations = 1000)

	Df	SumsOfSqs	MeanSqs	F.Model	R^2	Pr(>F)
**st_count_mean**	1	0.110218487813067	0.110218487813067	0.609958292120707	0.00113246753560745	0.533466533466533
**Residuals**	538	97.2157395176384	0.180698400590406		0.998867532464393	
**Total**	539	97.3259580054515			1	

H.

PERMANOVA model using the Simpson's D index of diversity as *dependent variable* and the following independent variable:
mean of the total counts of unique alleles across all genes per program (total_alleles_genes_mean)

Model = adonis(formula = simpson ~ total_alleles_genes_mean, data = d14, permutations = 1000)

	Df	SumsOfSqs	MeanSqs	F.Model	R^2	Pr(>F)
**total_alleles_genes_mean**	1	0.110218487813107	0.110218487813107	0.609958292120928	0.00113246753560786	0.533466533466533
**Residuals**	538	97.2157395176384	0.180698400590406		0.998867532464392	
**Total**	539	97.3259580054515			1	

I.

PERMANOVA model using the Simpson's D index of diversity as *dependent variable* and the following independent variable:
Standard deviation of the number of contigs (num_contigs_sd)

Model = adonis(formula = simpson ~ num_contigs_sd, data = d16, permutations = 1000)

	Df	SumsOfSqs	MeanSqs	F.Model	R^2	Pr(>F)
**num_contigs_sd**	1	18.1227489554589	18.1227489554589	123.101564380841	0.186206735868388	0.000999000999000999
**Residuals**	538	79.2032090499926	0.147217860687719		0.813793264131612	
**Total**	539	97.3259580054515			1	

J.

PERMANOVA model using the Simpson's D index of diversity as *dependent variable* and the following independent variable:
Standard deviation of the total counts for nucleotides per genomes (total_nucl_sd)

Model = adonis(formula = simpson ~ total_nucl_sd, data = d16, permutations = 1000)

	Df	SumsOfSqs	MeanSqs	F.Model	R^2	Pr(>F)
**total_nucl_sd**	1	12.5127094628339	12.5127094628339	79.3724778460993	0.128564976078972	0.000999000999000999
**Residuals**	538	84.8132485426176	0.157645443387765		0.871435023921028	
**Total**	539	97.3259580054515			1	

K.

PERMANOVA model using the Simpson's D index of diversity as *dependent variable* and the following independent variable:
Standard deviation of the average GC% per genome (gc_avg_sd)

Model = adonis(formula = simpson ~ gc_avg_sd, data = d16, permutations = 1000)

	Df	SumsOfSqs	MeanSqs	F.Model	R^2	Pr(>F)
**gc_avg_sd**	1	1.35976083775894	1.35976083775894	7.62301052146506	0.0139712042462791	0.001998001998002
**Residuals**	538	95.9661971676926	0.178375831166715		0.986028795753721	
**Total**	539	97.3259580054515			1	

Figure S13. PERMANOVA results measuring the association between *S. enterica* serovars (serovar), program, or genome-intrinsic and –extrinsic variables and the Simpson's D index of diversity.
PERMANOVA results demonstrating the association (*R*-squared and *p*-values) between the Simpson's D index of diversity (simpson) and: (A) bacterial serovar and program (mlst vs. stringMLST with all k-mer lengths); (B) bacterial serovar; (C) program (mlst vs. stringMLST with all k-mer lengths); (D) the median number of contigs (num_contigs_median); (E) the mean total number of nucleotides (total_nucl_mean); (F) the mean GC% content originally calculated per genome (gc_avg_mean); (G) the mean total count of STs present in each generated database (st_count_mean); (H) the mean total count of unique alleles (across all 7 loci) present in each generated database (total_alleles_genes_mean); (I) the standard deviation (SD) of the number of contigs (num_contigs_sd); (J) the SD of the total number of nucleotides (total_nucl_sd); (K) the SD of the GC% content per genome (gc_avg_sd). The median number of contigs, mean total number of nucleotides, and mean GC% content were grouped by serovar and batch (experimental replicate). The SD of the number of contigs, SD of the total number of nucleotides, and SD of GC% content were calculated by serovar only. The mean total count of STs and mean total count of unique alleles (across all 7 loci) present in each generated database were calculated after grouping by serovar, batch (three experimental replicates), and program. The Simpson's D index of diversity was calculated after grouping by program, serovar, and batch (three experimental replicates). All PERMANOVA models were run with 1,000 permutations.
