# Supplementary material for: Systems-Based Approach for Optimization of Assembly-Free Bacterial MLST Mapping
Source: Life (Basel). 2022 Apr 30;12(5):670. doi: 10.3390/life12050670 (PMC9147691; doi:10.3390/life12050670)
Supplement: Supplementary file 1 [file life-12-00670-s001.zip › Figure S14.pdf]

A.

PERMANOVA model using the proportion of non-classified STs as *dependent variable* and the following independent variables:
***S. enterica* serovars (serovar) and program as MLST or stringMLST (program)**

Model = adonis(formula = prop ~ serovar * program, data = d18b, permutations = 1000)

	Df	SumsOfSqs	MeanSqs	F.Model	R^2	Pr(>F)
**serovar**	19	26.8401628518863	1.41264015009928	37.1490819014527	0.353830505059659	0.000999000999000999
**program**	9	16.0133235978993	1.77925817754437	46.7902655582739	0.211101639270802	0.000999000999000999
**serovar:program**	163	20.8721297433185	0.12804987572588	3.36740770144276	0.275154672104759	0.000999000999000999
**Residuals**	319	12.1303726718492	0.038026246620217		0.15991318356478	
**Total**	510	75.8559888649533			1	

B.

PERMANOVA model using the proportion of non-classified STs as *dependent variable* and the following independent variable:
***S. enterica* serovars (serovar)**

Model = adonis(formula = prop ~ serovar, data = d18b, permutations = 1000)

	Df	SumsOfSqs	MeanSqs	F.Model	R^2	Pr(>F)
**serovar**	19	26.8401628518863	1.41264015009928	14.1506605134807	0.353830505059659	0.000999000999000999
**Residuals**	491	49.015826013067	0.0998285662180591		0.646169494940341	
**Total**	510	75.8559888649533			1	

C.

PERMANOVA model using the proportion of non-classified STs as *dependent variable* and the following independent variable: program as MLST or stringMLST (program)

Model = adonis(formula = prop ~ program, data = d18b, permutations = 1000)

	Df	SumsOfSqs	MeanSqs	F.Model	R^2	Pr(>F)
**program**	9	15.6969648506825	1.74410720563139	14.5247986372593	0.206931121531194	0.000999000999000999
**Residuals**	501	60.1590240142707	0.120077892244053		0.793068878468806	
**Total**	510	75.8559888649532			1	

D.

PERMANOVA model using the proportion of non-classified STs as *dependent variable* and the following independent variable: median of the number of contigs (num_contigs_median)

Model = adonis(formula = prop ~ num_contigs_median, data = d18b, permutations = 1000)

	Df	SumsOfSqs	MeanSqs	F.Model	R^2	Pr(>F)
**num_contigs_median**	1	0.391781222321214	0.391781222321214	2.64253277667546	0.00516480278200188	0.0609390609390609
**Residuals**	509	75.4642076426321	0.148259739965878		0.994835197217998	
**Total**	510	75.8559888649533			1	

E.

PERMANOVA model using the proportion of non-classified STs as *dependent variable* and the following independent variable:
mean of the total counts for nucleotides per genomes (total_nucl_mean)

Model = adonis(formula = prop ~ total_nucl_mean, data = d18b, permutations = 1000)

	Df	SumsOfSqs	MeanSqs	F.Model	R^2	Pr(>F)
**total_nucl_mean**	1	4.48323273411096	4.48323273411096	31.9725002279458	0.0591018955944596	0.000999000999000999
**Residuals**	509	71.3727561308423	0.140221524815014		0.94089810440554	
**Total**	510	75.8559888649533			1	

F.

PERMANOVA model using the proportion of non-classified STs as *dependent variable* and the following independent variable:
mean of the average GC% per genome (gc_avg_mean)

Model = adonis(formula = prop ~ gc_avg_mean, data = d18b, permutations = 1000)

	Df	SumsOfSqs	MeanSqs	F.Model	R^2	Pr(>F)
**gc_avg_mean**	1	0.291982834228857	0.291982834228857	1.96679967658226	0.00384917313184956	0.130869130869131
**Residuals**	509	75.5640060307244	0.148455807525981		0.99615082686815	
**Total**	510	75.8559888649533			1	

G.

PERMANOVA model using the proportion of non-classified STs as *dependent variable* and the following independent variable: mean of the total counts of unique STs per program (st_count_mean)

Model = adonis(formula = prop ~ st_count_mean, data = d18b, permutations = 1000)

	Df	SumsOfSqs	MeanSqs	F.Model	R^2	Pr(>F)
**st_count_mean**	1	6.6959987945063	6.6959987945063	49.2808541894239	0.0882725134125825	0.000999000999000999
**Residuals**	509	69.159990070447	0.135874243753334		0.911727486587418	
**Total**	510	75.8559888649533			1	

H.

PERMANOVA model using the proportion of non-classified STs as *dependent variable* and the following independent variable: mean of the total counts of unique alleles across all genes per program (total_alleles_genes_mean)

Model = adonis(formula = prop ~ total_alleles_genes_mean, data = d18b, permutations = 1000)

	Df	SumsOfSqs	MeanSqs	F.Model	R^2	Pr(>F)
**total_alleles_genes_mean**	1	6.69599879450637	6.69599879450637	49.2808541894246	0.0882725134125835	0.000999000999000999
**Residuals**	509	69.1599900704469	0.135874243753334		0.911727486587416	
**Total**	510	75.8559888649533			1	

I.

PERMANOVA model using the proportion of non-classified STs as *dependent variable* and the following independent variable:
Simpson's D index of diversity per *S. enterica* serovar (simpson)

Model = adonis(formula = prop ~ simpson, data = d18b, permutations = 1000)

	Df	SumsOfSqs	MeanSqs	F.Model	R^2	Pr(>F)
**simpson**	1	1.1819423928987	1.1819423928987	8.05646280613147	0.0155813985234959	0.000999000999000999
**Residuals**	509	74.6740464720546	0.146707360455903		0.984418601476504	
**Total**	510	75.8559888649533			1	

J.

PPERMANOVA model using the proportion of non-classified STs as *dependent variable* and the following independent variable:
Standard deviation of the number of contigs (num_contigs_sd)

Model = adonis(formula = prop ~ num_contigs_sd, data = d18c, permutations = 1000)

	Df	SumsOfSqs	MeanSqs	F.Model	R^2	Pr(>F)
**num_contigs_sd**	1	0.320546129525937	0.320546129525937	2.16001884704885	0.00422571947610632	0.115884115884116
**Residuals**	509	75.5354427354273	0.148399691032274		0.995774280523894	
**Total**	510	75.8559888649533			1	

K.

PERMANOVA model using the proportion of non-classified STs as *dependent variable* and the following independent variable:
Standard deviation of the total counts for nucleotides per genomes (total_nucl_sd)

Model = adonis(formula = prop ~ total_nucl_sd, data = d18c, permutations = 1000)

	Df	SumsOfSqs	MeanSqs	F.Model	R^2	Pr(>F)
**total_nucl_sd**	1	0.54987638138005	0.54987638138005	3.71665816879736	0.00724895146194715	0.043956043956044
**Residuals**	509	75.3061124835732	0.14794914043924		0.992751048538053	
**Total**	510	75.8559888649533			1	

L.

PermPERMANOVA model using the proportion of non-classified STs as *dependent variable* and the following independent variable:
Standard deviation of the average GC% per genome (gc_avg_sd)

Model = adonis(formula = prop ~ gc_avg_sd, data = d18c, permutations = 1000)

	Df	SumsOfSqs	MeanSqs	F.Model	R^2	Pr(>F)
**gc_avg_sd**	1	0.332391331936203	0.332391331936203	2.24018973515612	0.00438187329583113	0.0929070929070929
**Residuals**	509	75.5235975330171	0.148376419514768		0.995618126704169	
**Total**	510	75.8559888649533			1	

Figure S14. PERMANOVA results measuring the association between *S. enterica* serovars (serovar), program, or genome-intrinsic and –extrinsic variables and the proportion of non-classified STs.
PERMANOVA results demonstrating the association (*R*-squared and *p*-values) between the proportion of non-classified STs (prop) with: (A) bacterial serovar and program (mlst vs. stringMLST with all k-mer lengths); (B) bacterial serovar; (C) program (mlst vs. stringMLST with all k-mer lengths); (D) the median number of

contigs (num_contigs_median); (E) the mean total number of nucleotides (total_nucl_mean); (F) the mean GC% content originally calculated per genome (gc_avg_mean); (G) the mean total count of STs present in each generated database (st_count_mean); (H) the mean total count of unique alleles (across all 7 loci) present in each generated database (total_alleles_genes_mean); (I) the Simpson's D index of diversity (simpson); (J) the standard deviation (SD) of the number of contigs (num_contigs_sd); (K) the SD of the total number of nucleotides (total_nucl_sd); (L) the SD of the GC% content per genome (gc_avg_sd). The median number of contigs, mean total number of nucleotides, and mean GC% content were grouped by serovar and batch (experimental replicate). The SD of the number of contigs, SD of the total number of nucleotides, and SD of GC% content were calculated by serovar only. The mean total count of STs and mean total count of unique alleles (across all 7 loci) present in each generated database were calculated after grouping by serovar, batch (three experimental replicates), and program. The Simpson's D index of diversity was calculated after grouping by program, serovar, and batch (three experimental replicates). All PERMANOVA models were run with 1,000 permutations.
