# Supplementary material for: Systems-Based Approach for Optimization of Assembly-Free Bacterial MLST Mapping
Source: Life (Basel). 2022 Apr 30;12(5):670. doi: 10.3390/life12050670 (PMC9147691; doi:10.3390/life12050670)
Supplement: Supplementary file 1 [file life-12-00670-s001.zip › Figure S15.pdf]

A.

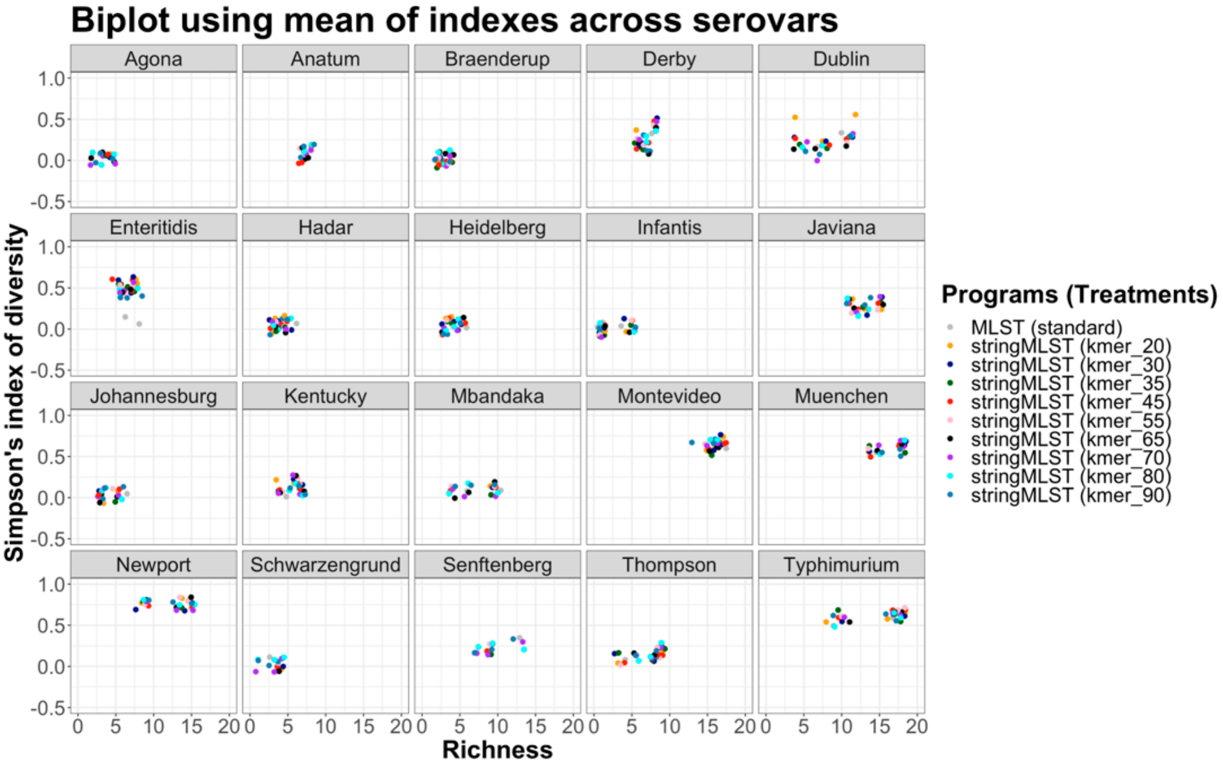

Biplot using mean of indexes across serovars
Agona
Anatum
Braenderup
Derby
Dublin
Enteritidis
Hadar
Heidelberg
Infantis
Javiana
Johannesburg
Kentucky
Mbandaka
Montevideo
Muenchen
Newport
Schwarzengrund
Senftenberg
Thompson
Typhimurium
Simpson's index of diversity
Richness
Programs (Treatments)
MLST (standard)
stringMLST (kmer_20)
stringMLST (kmer_30)
stringMLST (kmer_35)
stringMLST (kmer_45)
stringMLST (kmer_55)
stringMLST (kmer_65)
stringMLST (kmer_70)
stringMLST (kmer_80)
stringMLST (kmer_90)

B.

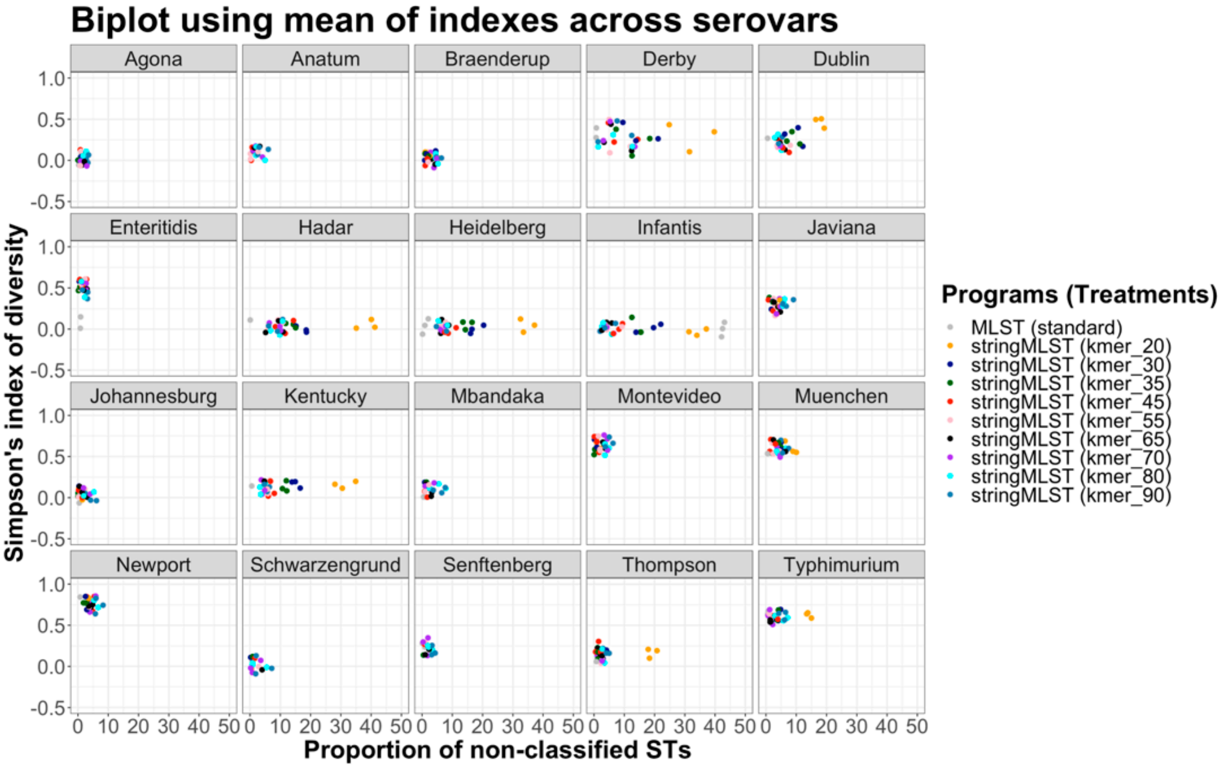

Biplot using mean of indexes across serovars
Agona
Anatum
Braenderup
Derby
Dublin
Enteritidis
Hadar
Heidelberg
Infantis
Javiana
Johannesburg
Kentucky
Mbandaka
Montevideo
Muenchen
Newport
Schwarzengrund
Senftenberg
Thompson
Typhimurium
Simpson's index of diversity
Proportion of non-classified STs
Programs (Treatments)
MLST (standard)
stringMLST (kmer_20)
stringMLST (kmer_30)
stringMLST (kmer_35)
stringMLST (kmer_45)
stringMLST (kmer_55)
stringMLST (kmer_65)
stringMLST (kmer_70)
stringMLST (kmer_80)
stringMLST (kmer_90)

C.

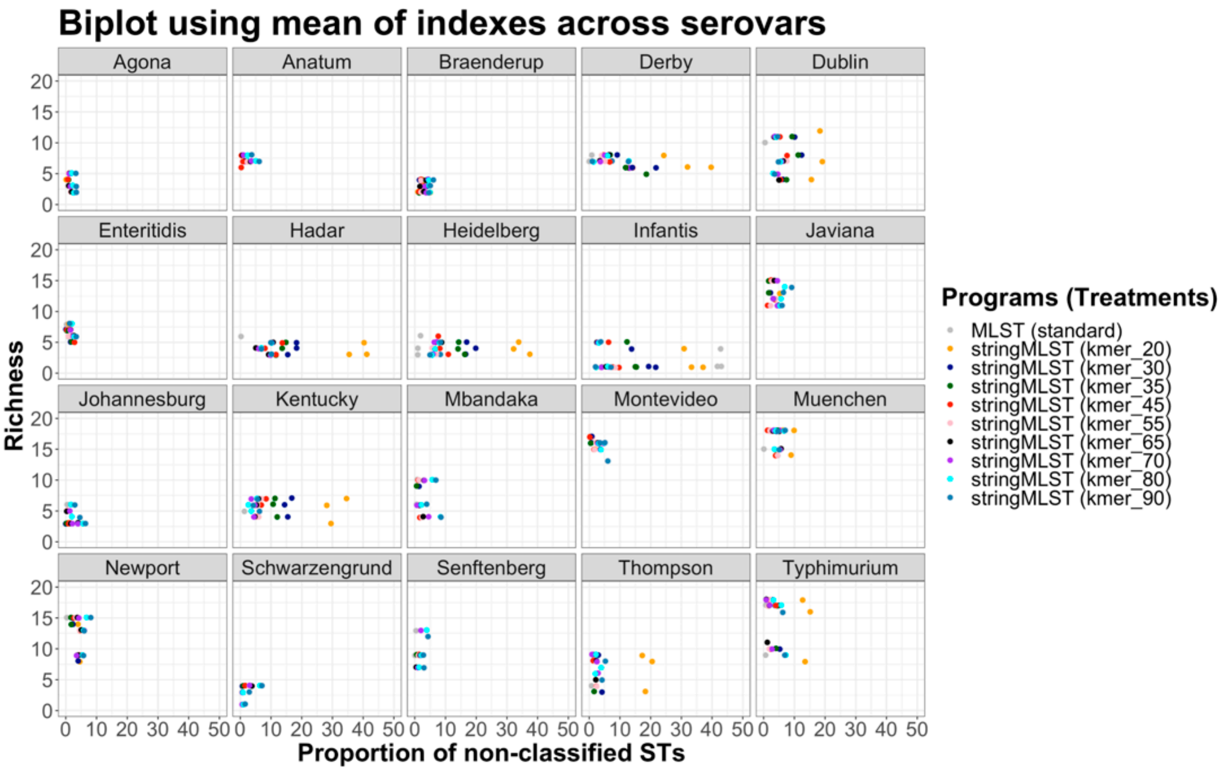
Biplot using mean of indexes across serovars
Agona
Anatum
Braenderup
Derby
Dublin
Enteritidis
Hadar
Heidelberg
Infantis
Javiana
Johannesburg
Kentucky
Mbandaka
Montevideo
Muenchen
Newport
Schwarzengrund
Senftenberg
Thompson
Typhimurium
Richness
Proportion of non-classified STs
Programs (Treatments)
MLST (standard)
stringMLST (kmer_20)
stringMLST (kmer_30)
stringMLST (kmer_35)
stringMLST (kmer_45)
stringMLST (kmer_55)
stringMLST (kmer_65)
stringMLST (kmer_70)
stringMLST (kmer_80)
stringMLST (kmer_90)

D.

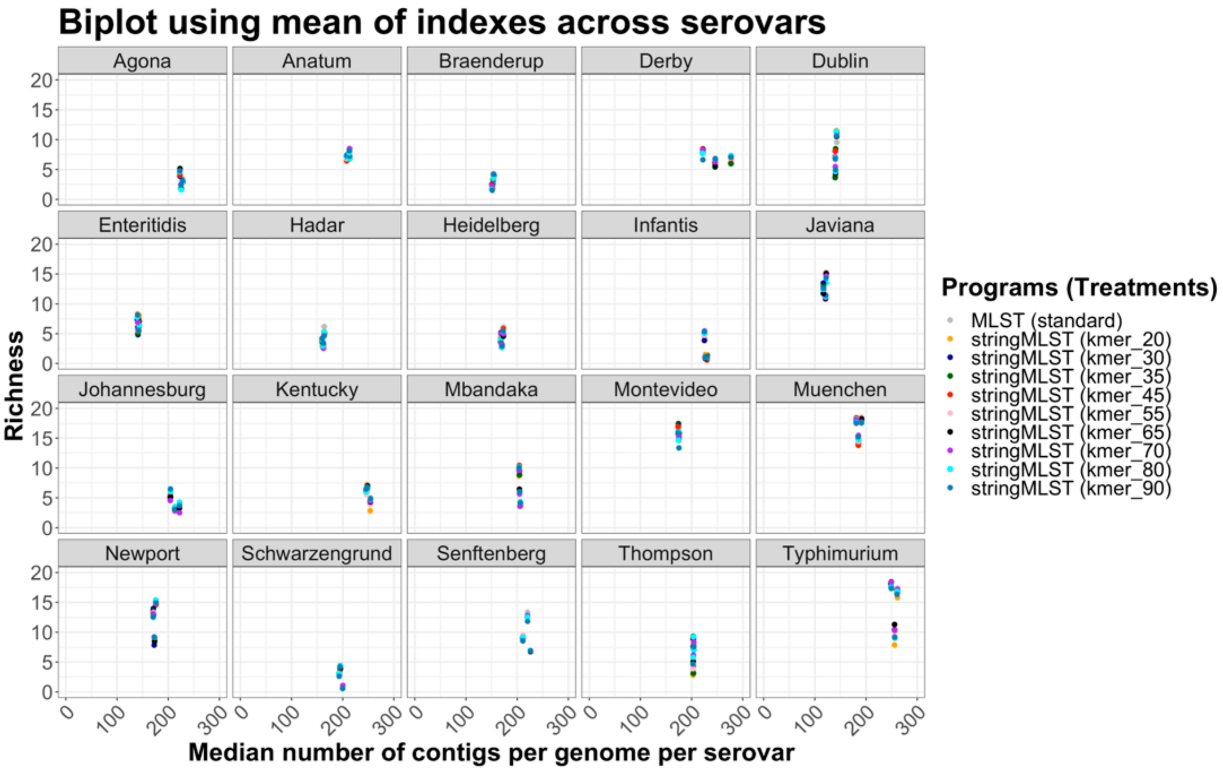
Biplot using mean of indexes across serovars
Agona
Anatum
Braenderup
Derby
Dublin
Enteritidis
Hadar
Heidelberg
Infantis
Javiana
Johannesburg
Kentucky
Mbandaka
Montevideo
Muenchen
Newport
Schwarzengrund
Senftenberg
Thompson
Typhimurium
Richness
Median number of contigs per genome per serovar
Programs (Treatments)
MLST (standard)
stringMLST (kmer_20)
stringMLST (kmer_30)
stringMLST (kmer_35)
stringMLST (kmer_45)
stringMLST (kmer_55)
stringMLST (kmer_65)
stringMLST (kmer_70)
stringMLST (kmer_80)
stringMLST (kmer_90)

E.

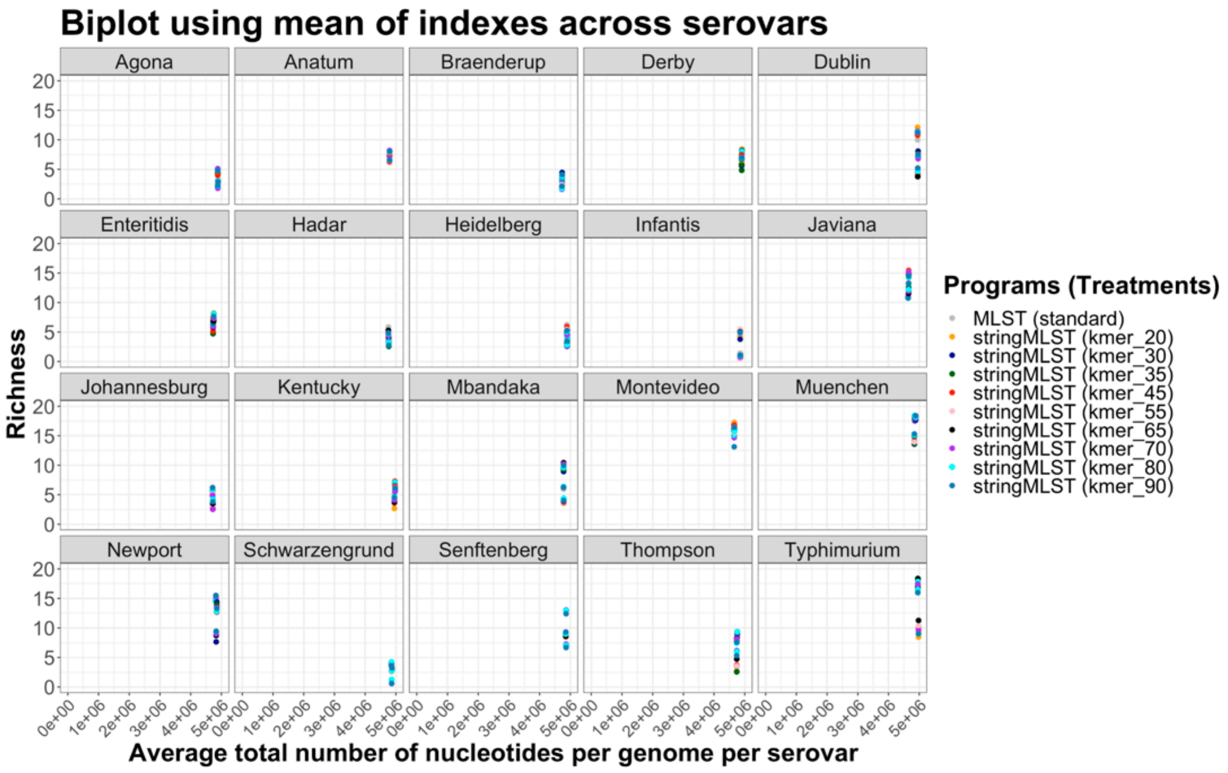
Biplot using mean of indexes across serovars
Agona
Anatum
Braenderup
Derby
Dublin
Enteritidis
Hadar
Heidelberg
Infantis
Javiana
Johannesburg
Kentucky
Mbandaka
Montevideo
Muenchen
Newport
Schwarzengrund
Senftenberg
Thompson
Typhimurium
Richness
Average total number of nucleotides per genome per serovar
Programs (Treatments)
MLST (standard)
stringMLST (kmer_20)
stringMLST (kmer_30)
stringMLST (kmer_35)
stringMLST (kmer_45)
stringMLST (kmer_55)
stringMLST (kmer_65)
stringMLST (kmer_70)
stringMLST (kmer_80)
stringMLST (kmer_90)

F.

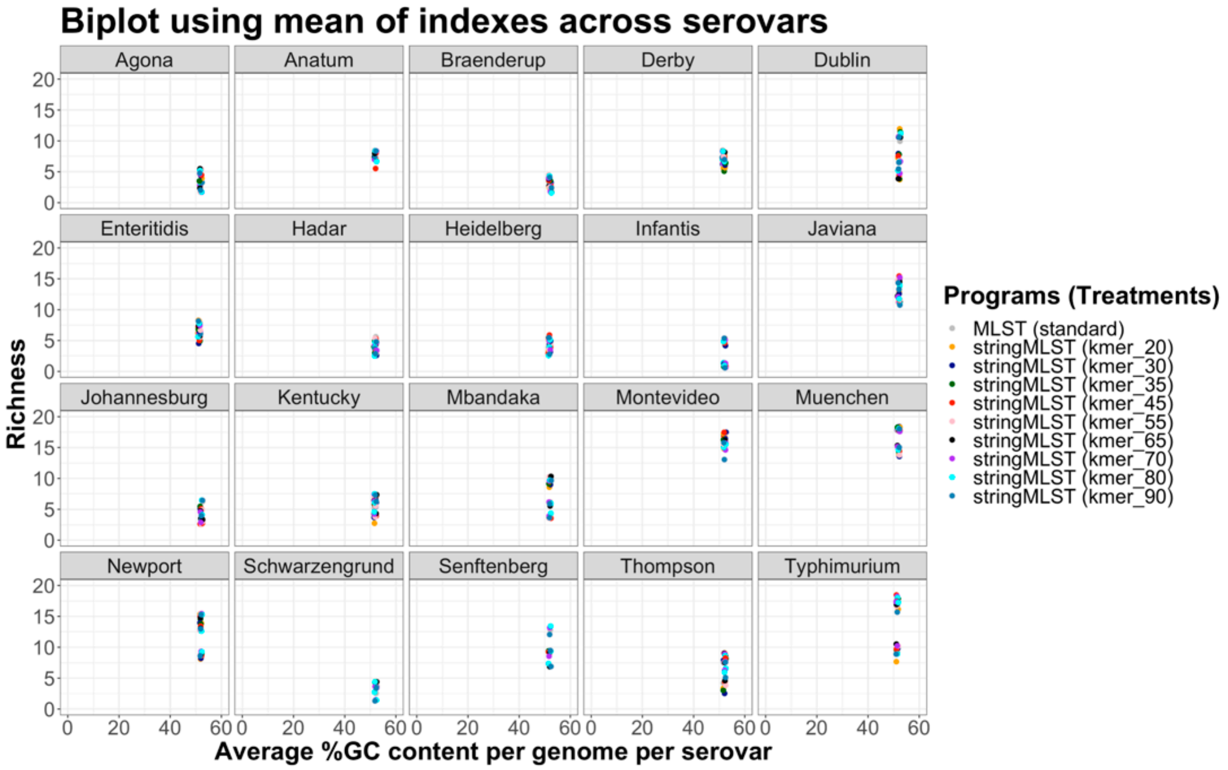
Biplot using mean of indexes across serovars
Agona
Anatum
Braenderup
Derby
Dublin
Enteritidis
Hadar
Heidelberg
Infantis
Javiana
Johannesburg
Kentucky
Mbandaka
Montevideo
Muenchen
Newport
Schwarzengrund
Senftenberg
Thompson
Typhimurium
Richness
Average %GC content per genome per serovar
Programs (Treatments)
MLST (standard)
stringMLST (kmer_20)
stringMLST (kmer_30)
stringMLST (kmer_35)
stringMLST (kmer_45)
stringMLST (kmer_55)
stringMLST (kmer_65)
stringMLST (kmer_70)
stringMLST (kmer_80)
stringMLST (kmer_90)

G.

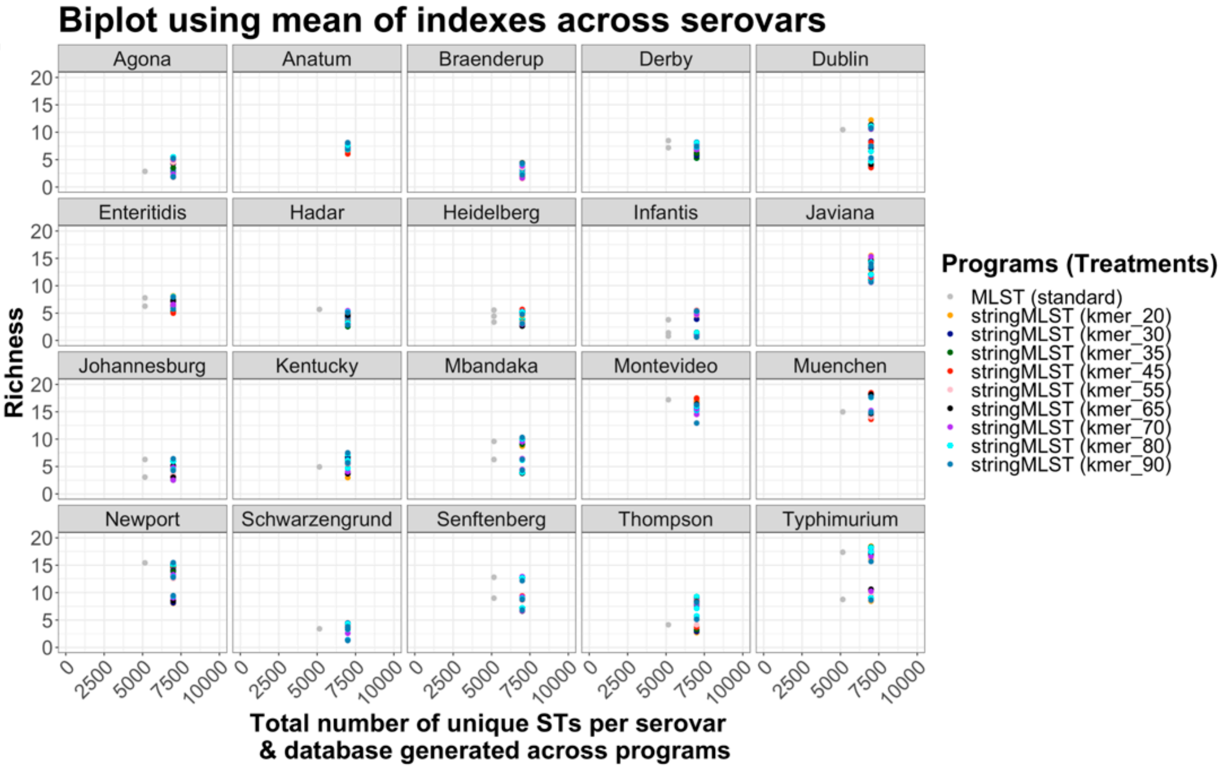
Biplot using mean of indexes across serovars
Agona
Anatum
Braenderup
Derby
Dublin
Enteritidis
Hadar
Heidelberg
Infantis
Javiana
Johannesburg
Kentucky
Mbandaka
Montevideo
Muenchen
Newport
Schwarzengrund
Senftenberg
Thompson
Typhimurium
Richness
0
5
10
15
20
0
2500
5000
7500
10000
Total number of unique STs per serovar
& database generated across programs
Programs (Treatments)
MLST (standard)
stringMLST (kmer_20)
stringMLST (kmer_30)
stringMLST (kmer_35)
stringMLST (kmer_45)
stringMLST (kmer_55)
stringMLST (kmer_65)
stringMLST (kmer_70)
stringMLST (kmer_80)
stringMLST (kmer_90)

H.

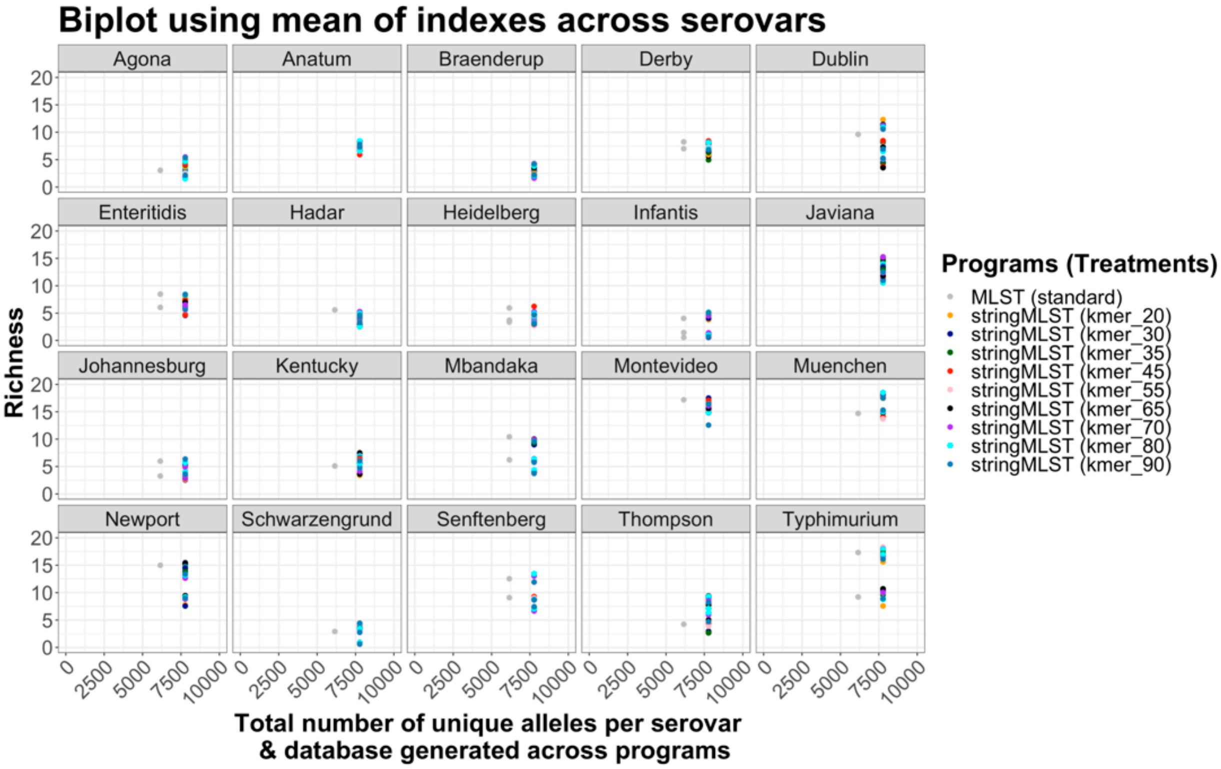
Biplot using mean of indexes across serovars
Agona
Anatum
Braenderup
Derby
Dublin
Enteritidis
Hadar
Heidelberg
Infantis
Javiana
Johannesburg
Kentucky
Mbandaka
Montevideo
Muenchen
Newport
Schwarzengrund
Senftenberg
Thompson
Typhimurium
Richness
0
5
10
15
20
0
2500
5000
7500
10000
Total number of unique alleles per serovar
& database generated across programs
Programs (Treatments)
MLST (standard)
stringMLST (kmer_20)
stringMLST (kmer_30)
stringMLST (kmer_35)
stringMLST (kmer_45)
stringMLST (kmer_55)
stringMLST (kmer_65)
stringMLST (kmer_70)
stringMLST (kmer_80)
stringMLST (kmer_90)

I.

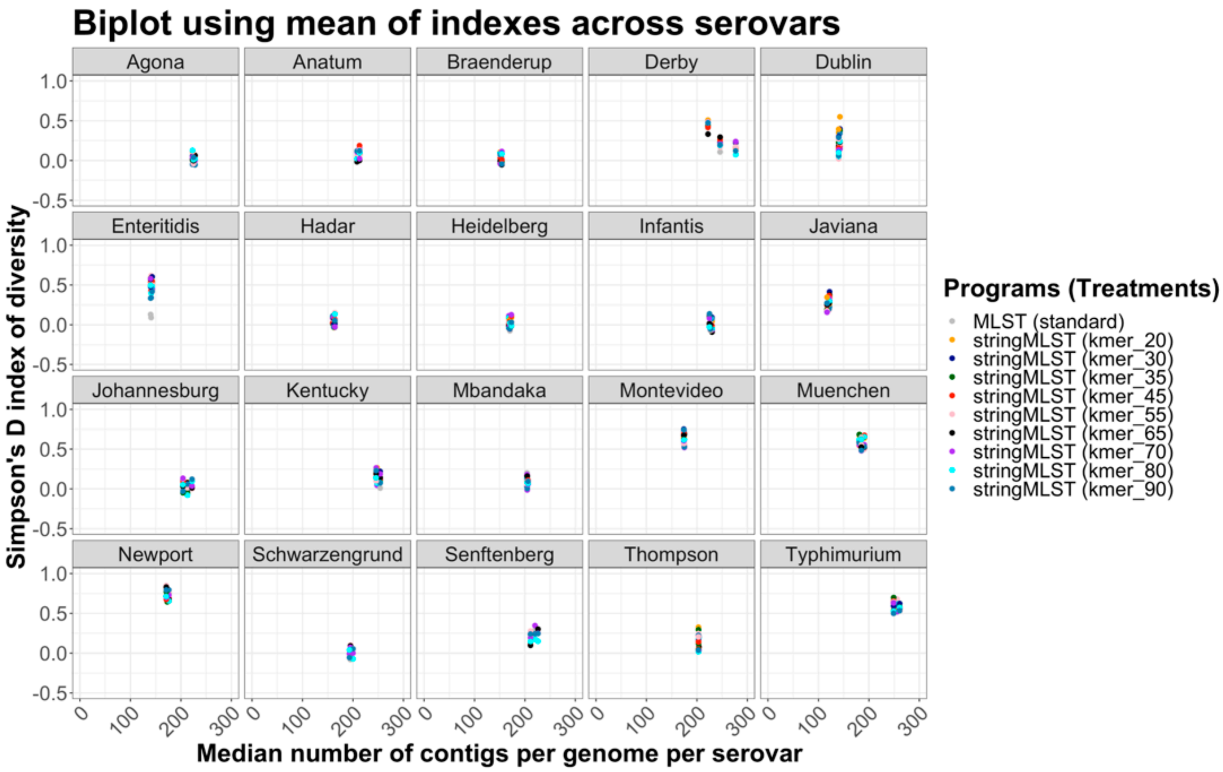
Biplot using mean of indexes across serovars
Agona
Anatum
Braenderup
Derby
Dublin
Enteritidis
Hadar
Heidelberg
Infantis
Javiana
Johannesburg
Kentucky
Mbandaka
Montevideo
Muenchen
Newport
Schwarzengrund
Senftenberg
Thompson
Typhimurium
Simpson's D index of diversity
Median number of contigs per genome per serovar
Programs (Treatments)
MLST (standard)
stringMLST (kmer_20)
stringMLST (kmer_30)
stringMLST (kmer_35)
stringMLST (kmer_45)
stringMLST (kmer_55)
stringMLST (kmer_65)
stringMLST (kmer_70)
stringMLST (kmer_80)
stringMLST (kmer_90)

J.

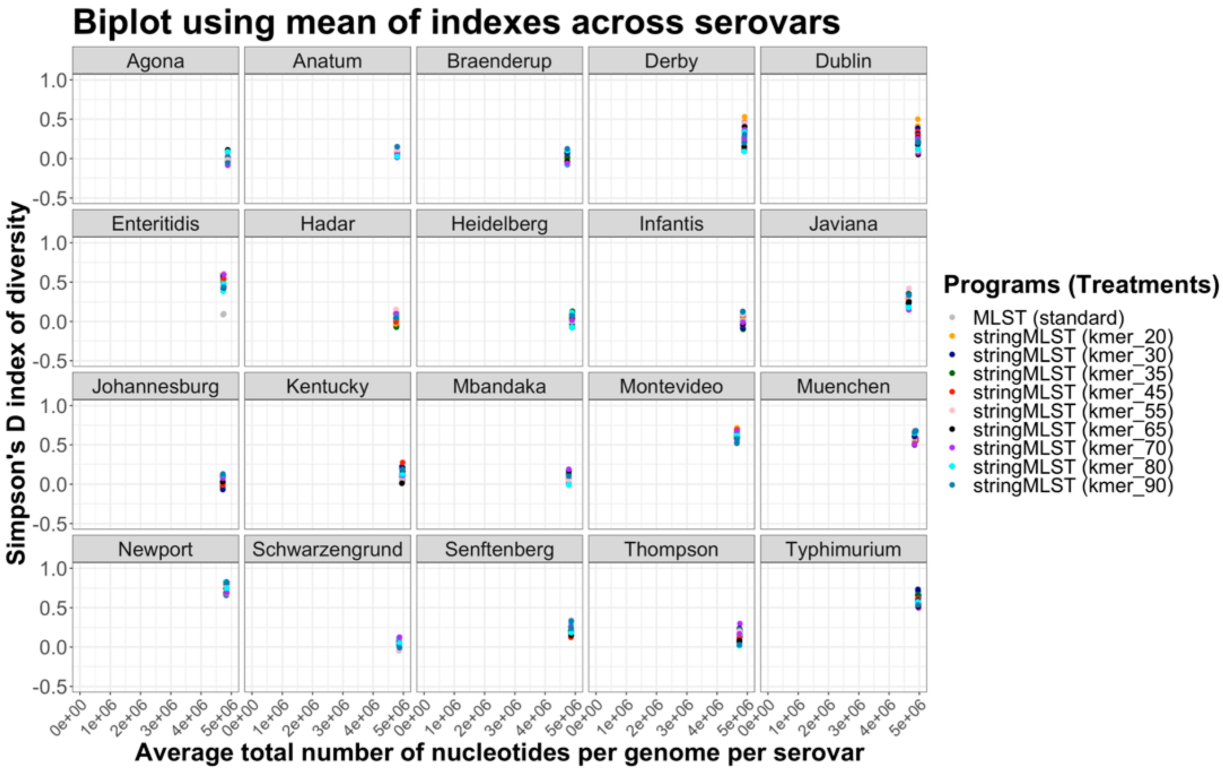
Biplot using mean of indexes across serovars
Agona
Anatum
Braenderup
Derby
Dublin
Enteritidis
Hadar
Heidelberg
Infantis
Javiana
Johannesburg
Kentucky
Mbandaka
Montevideo
Muenchen
Newport
Schwarzengrund
Senftenberg
Thompson
Typhimurium
Simpson's D index of diversity
Average total number of nucleotides per genome per serovar
Programs (Treatments)
MLST (standard)
stringMLST (kmer_20)
stringMLST (kmer_30)
stringMLST (kmer_35)
stringMLST (kmer_45)
stringMLST (kmer_55)
stringMLST (kmer_65)
stringMLST (kmer_70)
stringMLST (kmer_80)
stringMLST (kmer_90)

K.

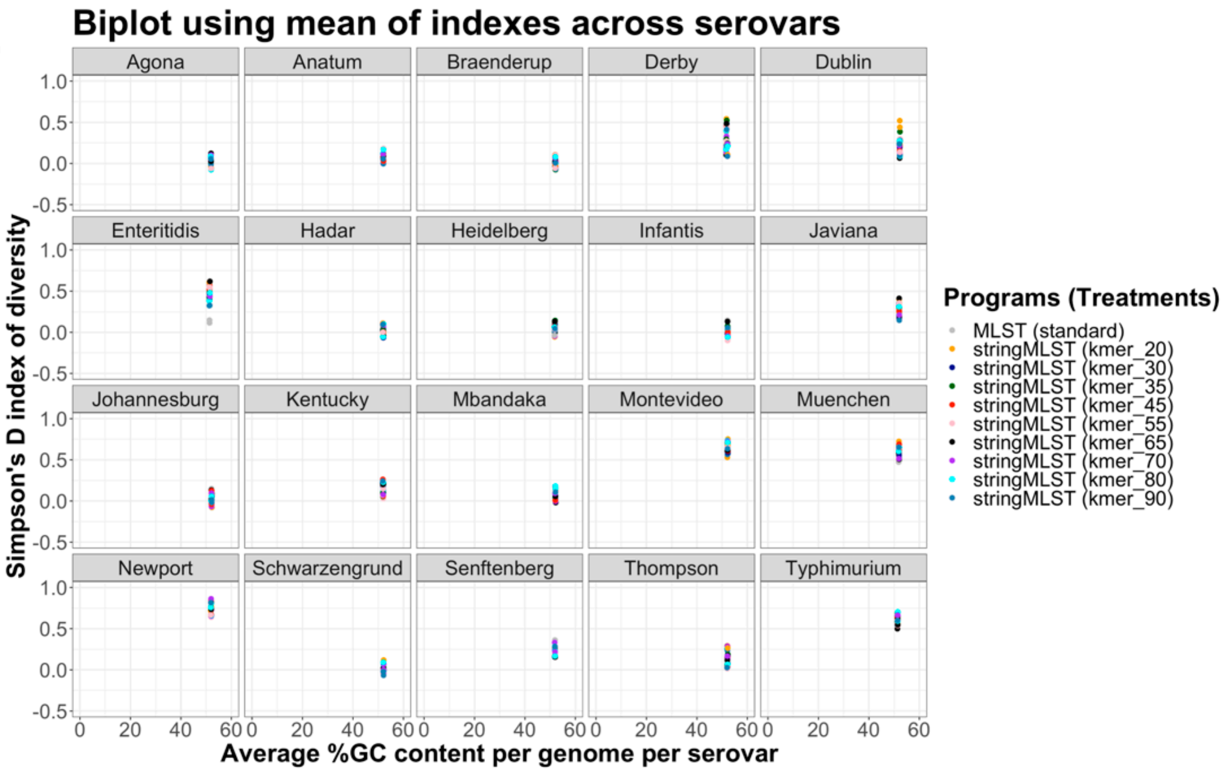
Biplot using mean of indexes across serovars
Agona
Anatum
Braenderup
Derby
Dublin
Enteritidis
Hadar
Heidelberg
Infantis
Javiana
Johannesburg
Kentucky
Mbandaka
Montevideo
Muenchen
Newport
Schwarzengrund
Senftenberg
Thompson
Typhimurium
Simpson's D index of diversity
Average %GC content per genome per serovar
Programs (Treatments)
MLST (standard)
stringMLST (kmer_20)
stringMLST (kmer_30)
stringMLST (kmer_35)
stringMLST (kmer_45)
stringMLST (kmer_55)
stringMLST (kmer_65)
stringMLST (kmer_70)
stringMLST (kmer_80)
stringMLST (kmer_90)

L.

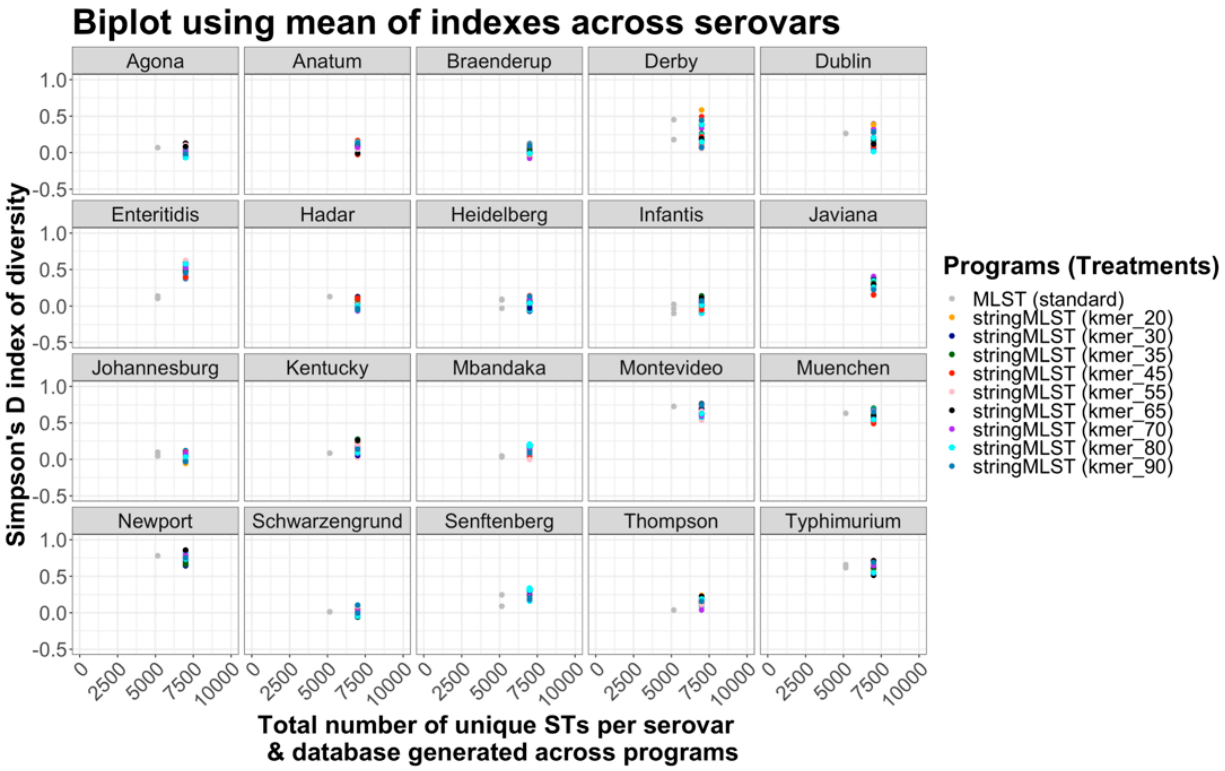
Biplot using mean of indexes across serovars
Agona
Anatum
Braenderup
Derby
Dublin
Enteritidis
Hadar
Heidelberg
Infantis
Javiana
Johannesburg
Kentucky
Mbandaka
Montevideo
Muenchen
Newport
Schwarzengrund
Senftenberg
Thompson
Typhimurium
Simpson's D index of diversity
Total number of unique STs per serovar
& database generated across programs
Programs (Treatments)
MLST (standard)
stringMLST (kmer_20)
stringMLST (kmer_30)
stringMLST (kmer_35)
stringMLST (kmer_45)
stringMLST (kmer_55)
stringMLST (kmer_65)
stringMLST (kmer_70)
stringMLST (kmer_80)
stringMLST (kmer_90)

M.

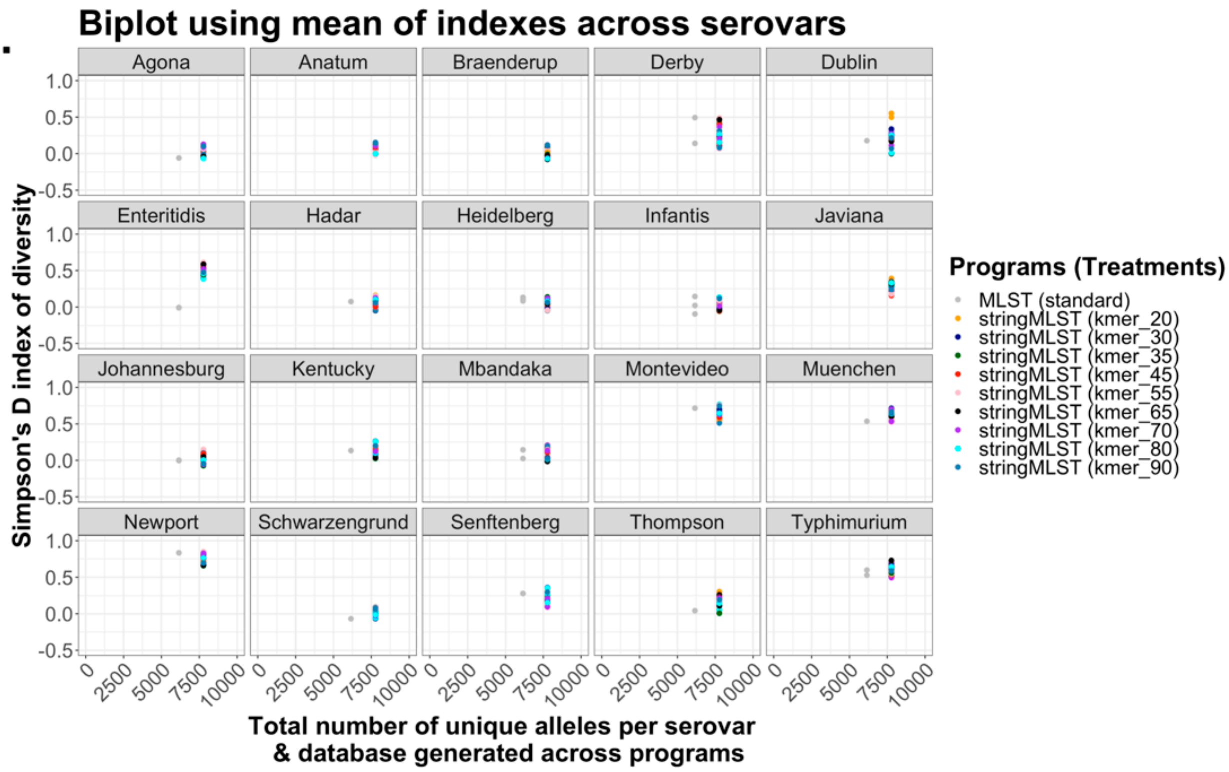
Biplot using mean of indexes across serovars
Agona
Anatum
Braenderup
Derby
Dublin
Enteritidis
Hadar
Heidelberg
Infantis
Javiana
Johannesburg
Kentucky
Mbandaka
Montevideo
Muenchen
Newport
Schwarzengrund
Senftenberg
Thompson
Typhimurium
Simpson's D index of diversity
Total number of unique alleles per serovar & database generated across programs
Programs (Treatments)
MLST (standard)
stringMLST (kmer_20)
stringMLST (kmer_30)
stringMLST (kmer_35)
stringMLST (kmer_45)
stringMLST (kmer_55)
stringMLST (kmer_65)
stringMLST (kmer_70)
stringMLST (kmer_80)
stringMLST (kmer_90)

N.

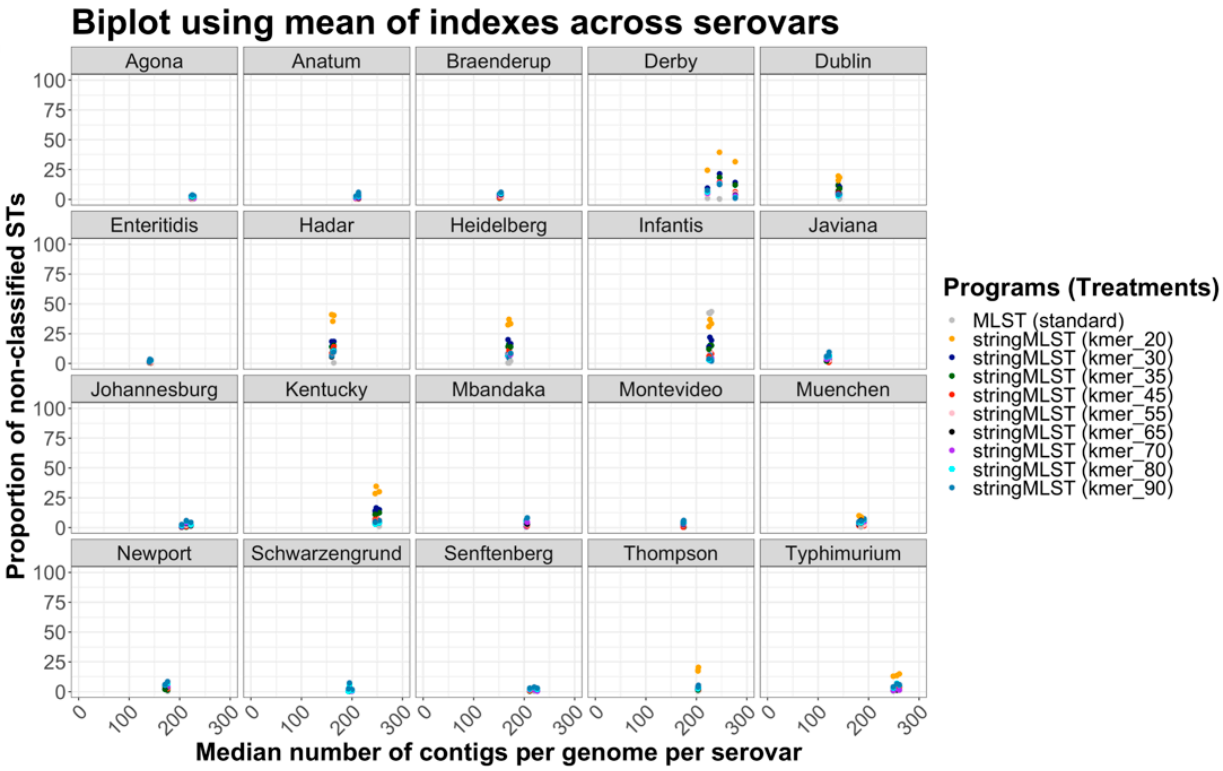
Biplot using mean of indexes across serovars
Agona
Anatum
Braenderup
Derby
Dublin
Enteritidis
Hadar
Heidelberg
Infantis
Javiana
Johannesburg
Kentucky
Mbandaka
Montevideo
Muenchen
Newport
Schwarzengrund
Senftenberg
Thompson
Typhimurium
Proportion of non-classified STs
Median number of contigs per genome per serovar
Programs (Treatments)
MLST (standard)
stringMLST (kmer_20)
stringMLST (kmer_30)
stringMLST (kmer_35)
stringMLST (kmer_45)
stringMLST (kmer_55)
stringMLST (kmer_65)
stringMLST (kmer_70)
stringMLST (kmer_80)
stringMLST (kmer_90)

O.

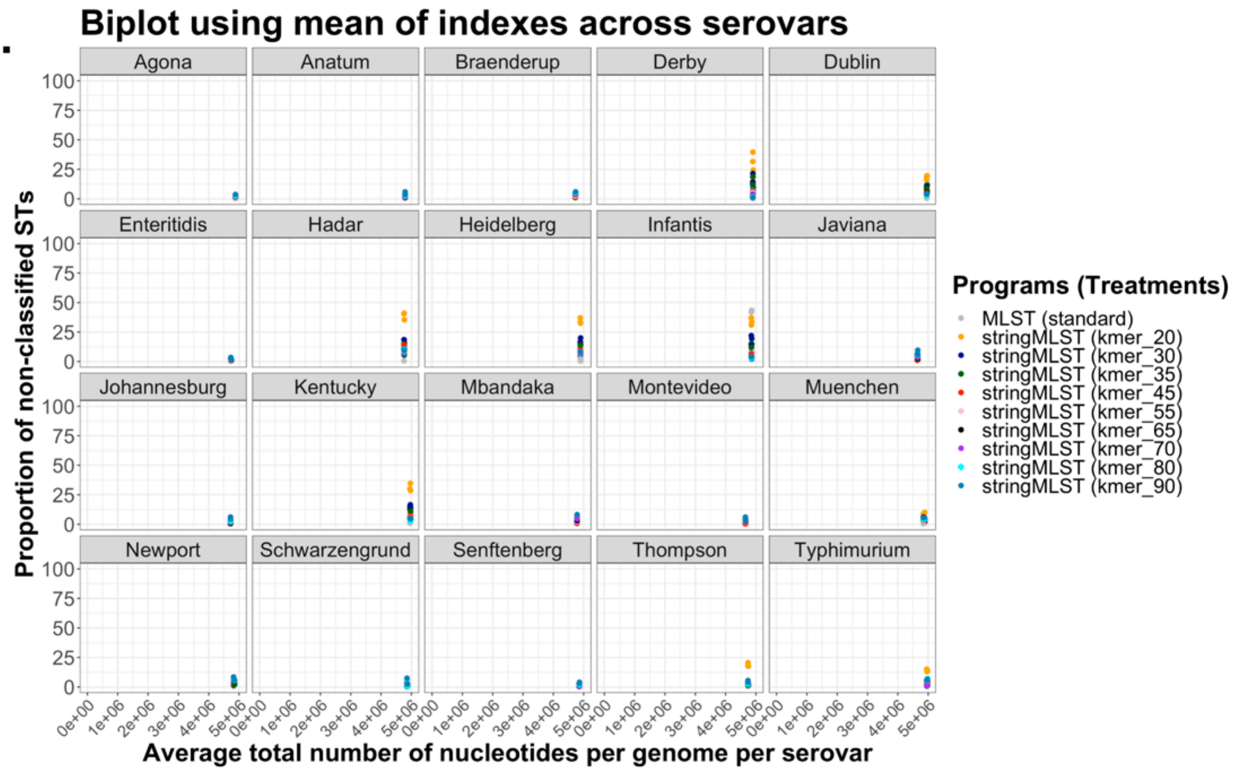
Biplot using mean of indexes across serovars
Agona
Anatum
Braenderup
Derby
Dublin
Enteritidis
Hadar
Heidelberg
Infantis
Javiana
Johannesburg
Kentucky
Mbandaka
Montevideo
Muenchen
Newport
Schwarzengrund
Senftenberg
Thompson
Typhimurium
Proportion of non-classified STs
Average total number of nucleotides per genome per serovar
Programs (Treatments)
MLST (standard)
stringMLST (kmer_20)
stringMLST (kmer_30)
stringMLST (kmer_35)
stringMLST (kmer_45)
stringMLST (kmer_55)
stringMLST (kmer_65)
stringMLST (kmer_70)
stringMLST (kmer_80)
stringMLST (kmer_90)

P.

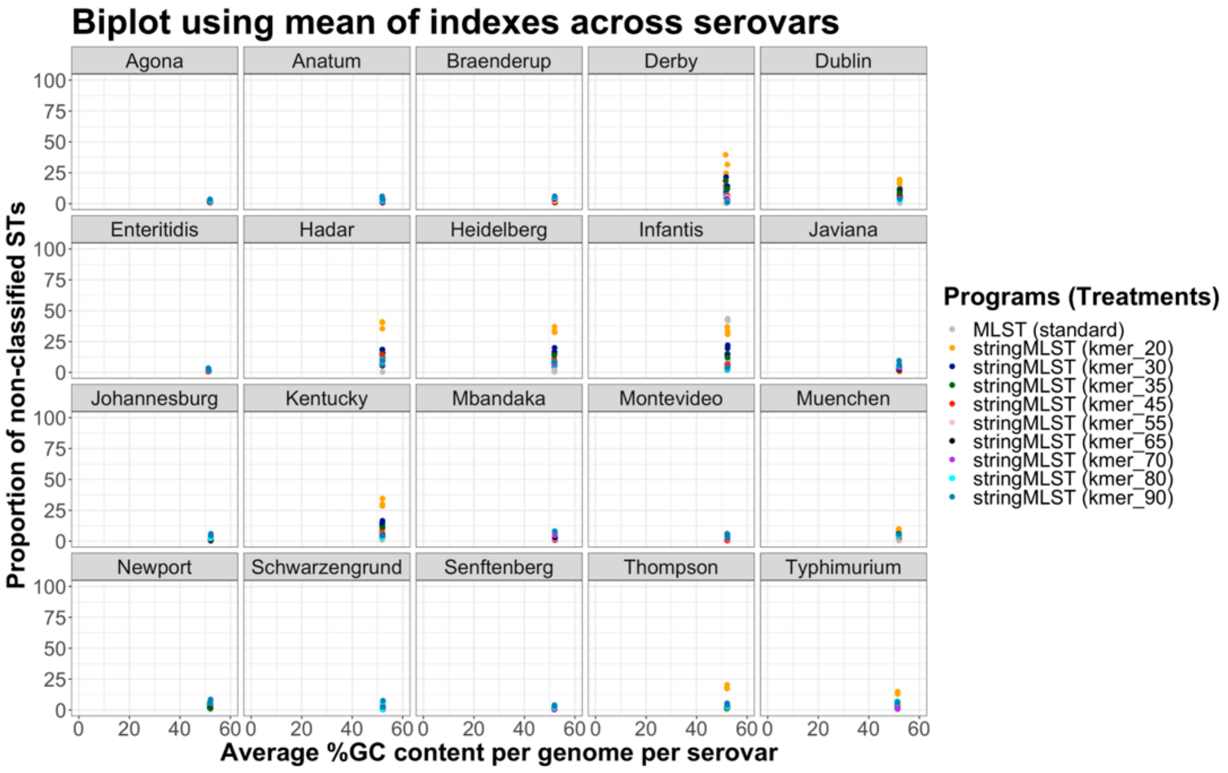
Biplot using mean of indexes across serovars
Agona
Anatum
Braenderup
Derby
Dublin
Enteritidis
Hadar
Heidelberg
Infantis
Javiana
Johannesburg
Kentucky
Mbandaka
Montevideo
Muenchen
Newport
Schwarzengrund
Senftenberg
Thompson
Typhimurium
Proportion of non-classified STs
Average %GC content per genome per serovar
Programs (Treatments)
MLST (standard)
stringMLST (kmer_20)
stringMLST (kmer_30)
stringMLST (kmer_35)
stringMLST (kmer_45)
stringMLST (kmer_55)
stringMLST (kmer_65)
stringMLST (kmer_70)
stringMLST (kmer_80)
stringMLST (kmer_90)

Q.

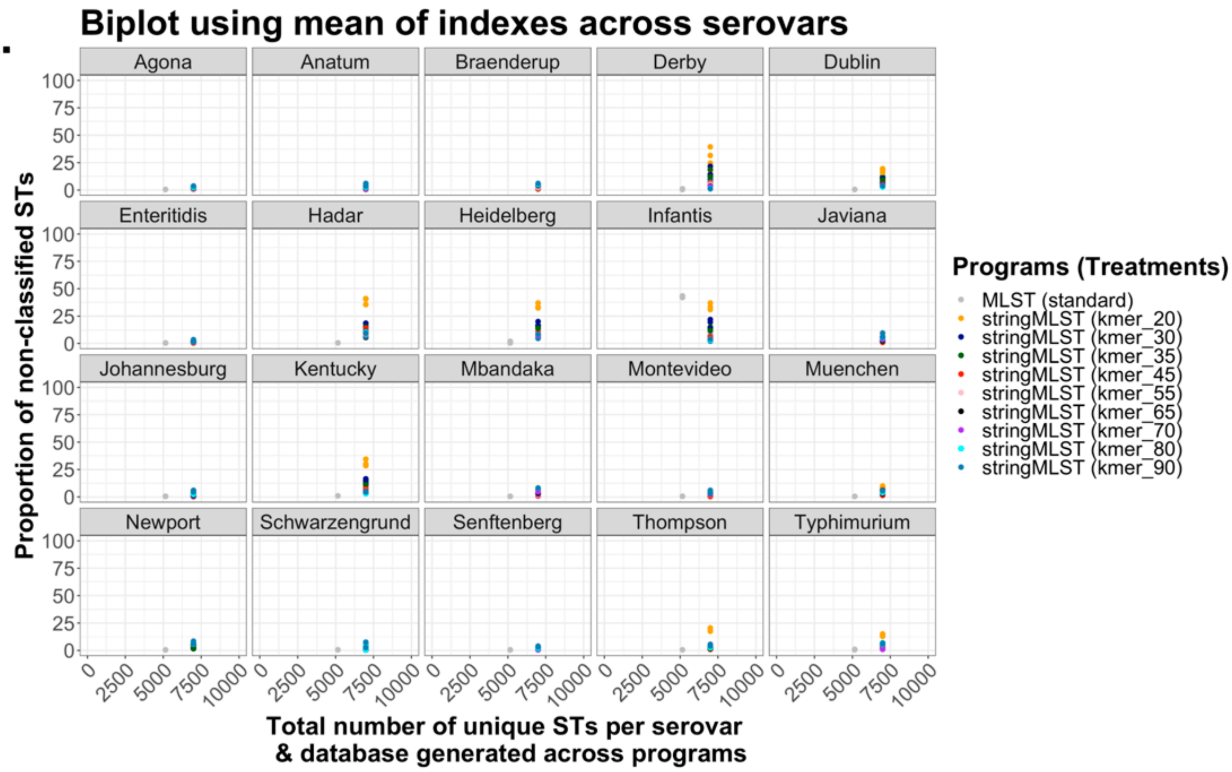
Biplot using mean of indexes across serovars
Agona
Anatum
Braenderup
Derby
Dublin
Enteritidis
Hadar
Heidelberg
Infantis
Javiana
Johannesburg
Kentucky
Mbandaka
Montevideo
Muenchen
Newport
Schwarzengrund
Senftenberg
Thompson
Typhimurium
Proportion of non-classified STs
Total number of unique STs per serovar & database generated across programs
Programs (Treatments)
MLST (standard)
stringMLST (kmer_20)
stringMLST (kmer_30)
stringMLST (kmer_35)
stringMLST (kmer_45)
stringMLST (kmer_55)
stringMLST (kmer_65)
stringMLST (kmer_70)
stringMLST (kmer_80)
stringMLST (kmer_90)

R.

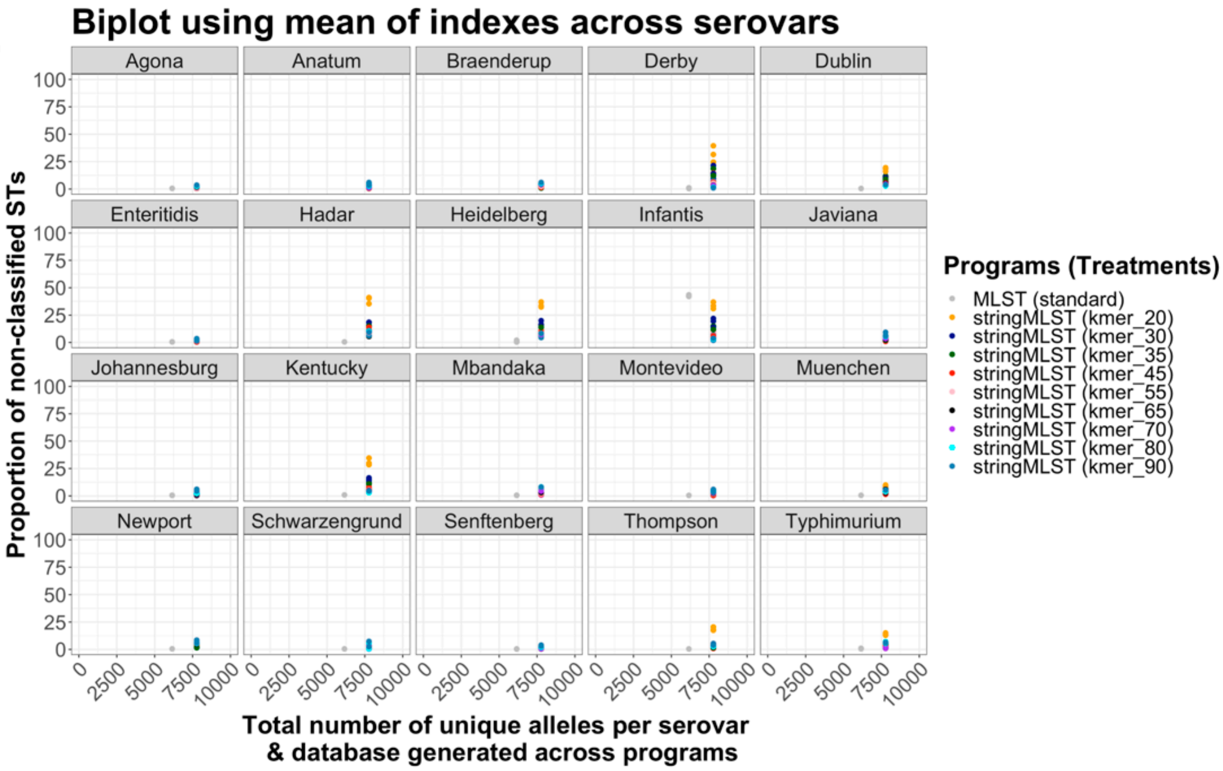
Biplot using mean of indexes across serovars
Agona
Anatum
Braenderup
Derby
Dublin
Enteritidis
Hadar
Heidelberg
Infantis
Javiana
Johannesburg
Kentucky
Mbandaka
Montevideo
Muenchen
Newport
Schwarzengrund
Senftenberg
Thompson
Typhimurium
Proportion of non-classified STs
Total number of unique alleles per serovar & database generated across programs
Programs (Treatments)
MLST (standard)
stringMLST (kmer_20)
stringMLST (kmer_30)
stringMLST (kmer_35)
stringMLST (kmer_45)
stringMLST (kmer_55)
stringMLST (kmer_65)
stringMLST (kmer_70)
stringMLST (kmer_80)
stringMLST (kmer_90)

Figure S15. Bivariate association between accuracy-based classification metrics and genome-intrinsic and –extrinsic variables across *S. enterica* serovars.
(A) Scatter plot demonstrating the relationship between the Simpson's D index of diversity (1 – *D*) vs. ST richness (Richness). (B) Scatter plot demonstrating the relationship between the Simpson's D index of diversity (1 – *D*) vs. proportion of non-classified STs. (C) Scatter plot demonstrating the relationship between the ST richness (Richness) vs. proportion of non-classified STs across *S. enterica* serovars and programs. (D) Scatter plot demonstrating the relationship between the ST richness (Richness) vs. median number of contigs per genome across *S. enterica* serovars and programs. (E) Scatter plot demonstrating the relationship between the ST richness (Richness) vs. the average total number of nucleotides per genome across *S. enterica* serovars and programs. (F) Scatter plot demonstrating the relationship between the ST richness (Richness) vs. the average GC% content per genome. (G) Scatter plot demonstrating the relationship between the ST richness (Richness) vs. the total number of unique STs in the database. (H) Scatter plot demonstrating the relationship between the ST richness (Richness) vs. the total number of unique alleles across all 7 loci in the database. (I) Scatter plot demonstrating the relationship between the Simpson's D index of diversity (1 – *D*) vs. the median number of contigs per genome. (J) Scatter plot demonstrating the relationship between the Simpson's D index of diversity (1 – *D)* vs. the average total number of nucleotides per genome. (K) Scatter plot demonstrating the relationship between the Simpson's D index of diversity (1 – *D)* vs. the average GC% content per genome. (L) Scatter plot demonstrating the relationship between the Simpson's D index of diversity (1 – *D)* vs. the total number of unique STs in the database. (M) Scatter plot demonstrating the relationship between the Simpson's D index of diversity (1 – *D)* vs. the total number of unique alleles across all 7 loci in the database. (N) Scatter plot demonstrating the relationship between the proportion of non-classified STs vs. the median number of contigs per genome. (O) Scatter plot demonstrating the relationship between the proportion of non-classified STs vs. the average total number of nucleotides per genome. (P) Scatter plot demonstrating the relationship between the proportion of non-classified STs vs. the average GC% content per genome. (Q) Scatter plot demonstrating the relationship between the proportion of non-classified STs vs. the total number of unique STs in the database. (R) Scatter plot demonstrating the relationship between the proportion of non-classified STs vs. the total number of unique alleles across all 7 loci in the database. All plots were stratified by *S. enterica* serovars and data points were colored by the program (mlst vs. stringMLST including all k-mer lengths).
