# Supplementary material for: Systems-Based Approach for Optimization of Assembly-Free Bacterial MLST Mapping
Source: Life (Basel). 2022 Apr 30;12(5):670. doi: 10.3390/life12050670 (PMC9147691; doi:10.3390/life12050670)
Supplement: Supplementary file 1 [file life-12-00670-s001.zip › Figure S16.pdf]

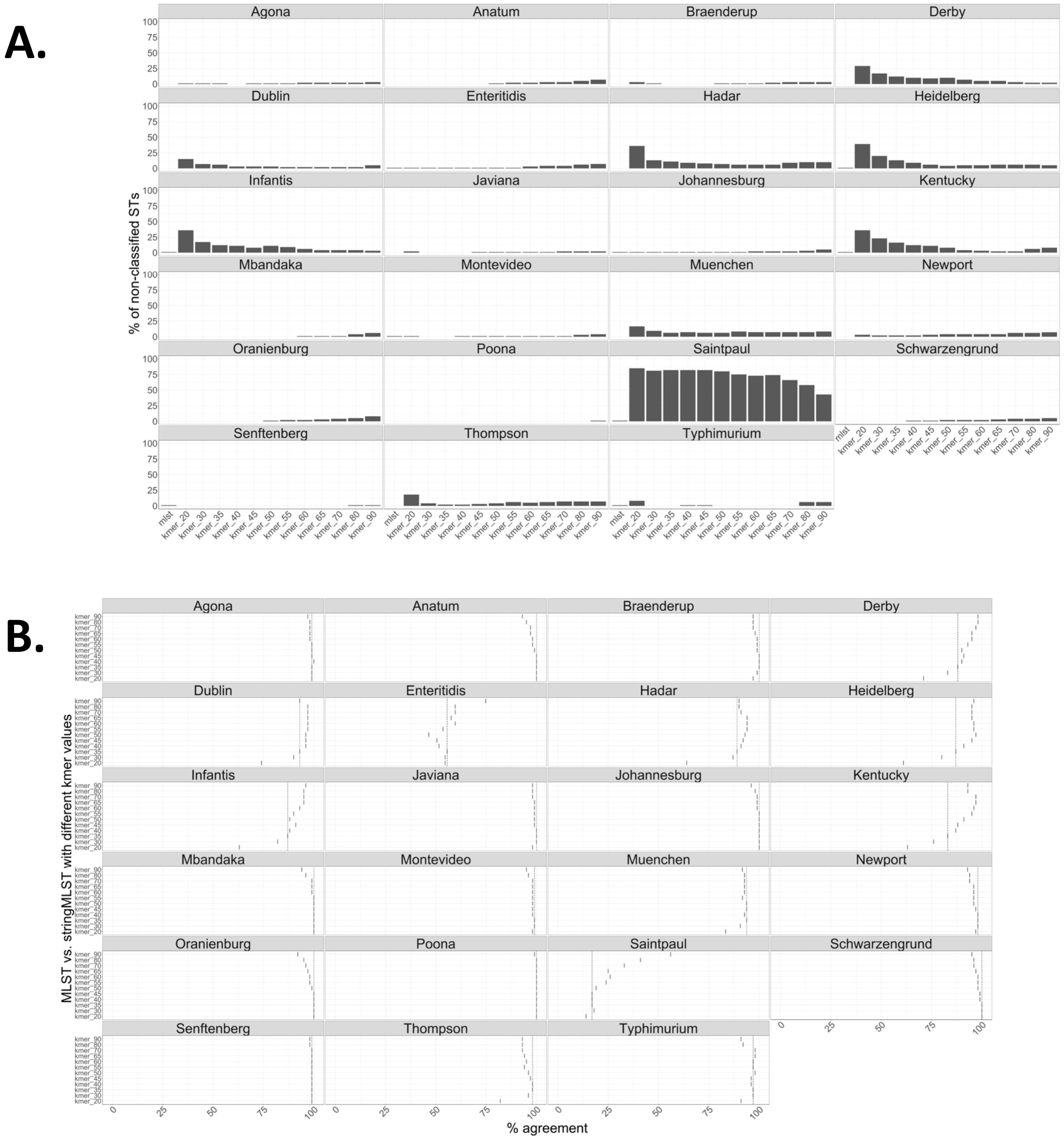
A.
% of non-classified STs
Agona
Anatum
Braenderup
Derby
Dublin
Enteritidis
Hadar
Heidelberg
Infantis
Javiana
Johannesburg
Kentucky
Mbandaka
Montevideo
Muenchen
Newport
Oranienburg
Poona
Saintpaul
Schwarzengrund
Senftenberg
Thompson
Typhimurium
0
25
50
75
100
mlst
kmer_20
kmer_30
kmer_35
kmer_40
kmer_45
kmer_50
kmer_55
kmer_60
kmer_65
kmer_70
kmer_80
kmer_90
B.
MLST vs. stringMLST with different kmer values
% agreement

C.

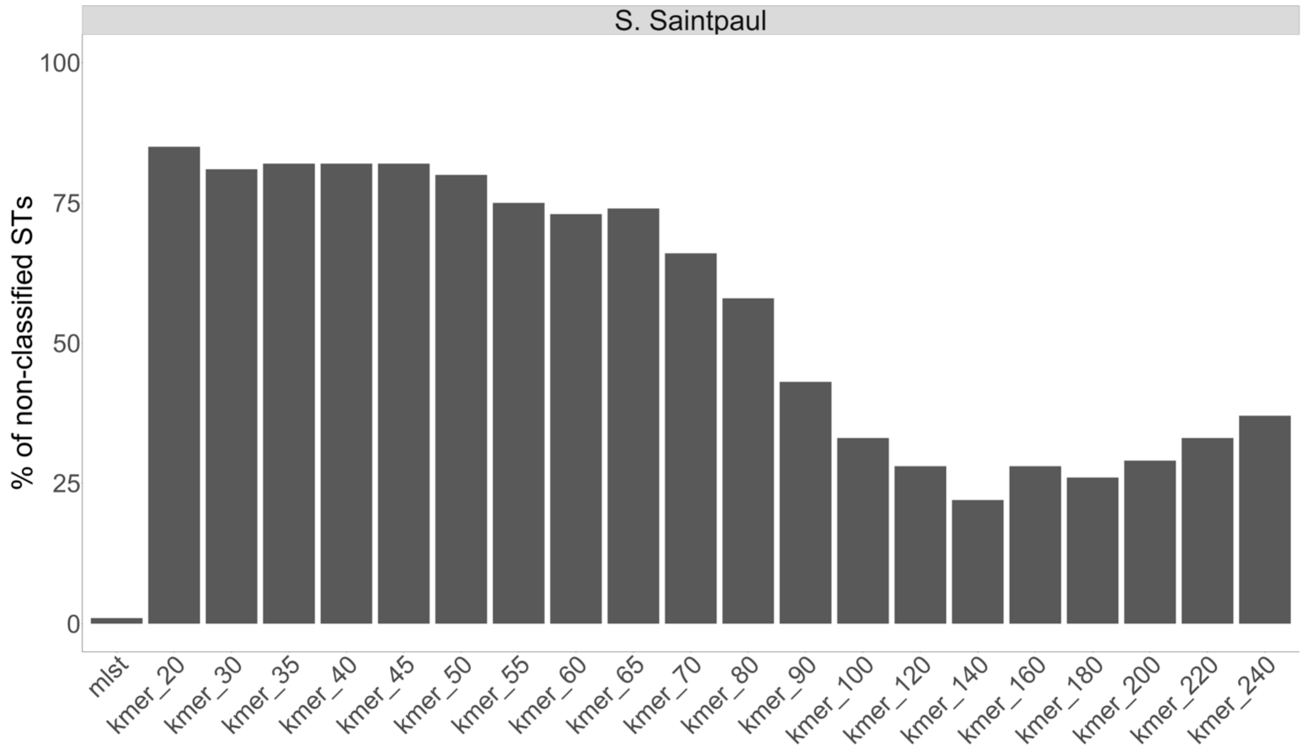
S. Saintpaul
% of non-classified STs
100
75
50
25
0
mlst
kmer_20
kmer_30
kmer_35
kmer_40
kmer_45
kmer_50
kmer_55
kmer_60
kmer_65
kmer_70
kmer_80
kmer_90
kmer_100
kmer_120
kmer_140
kmer_160
kmer_180
kmer_200
kmer_220
kmer_240

D.

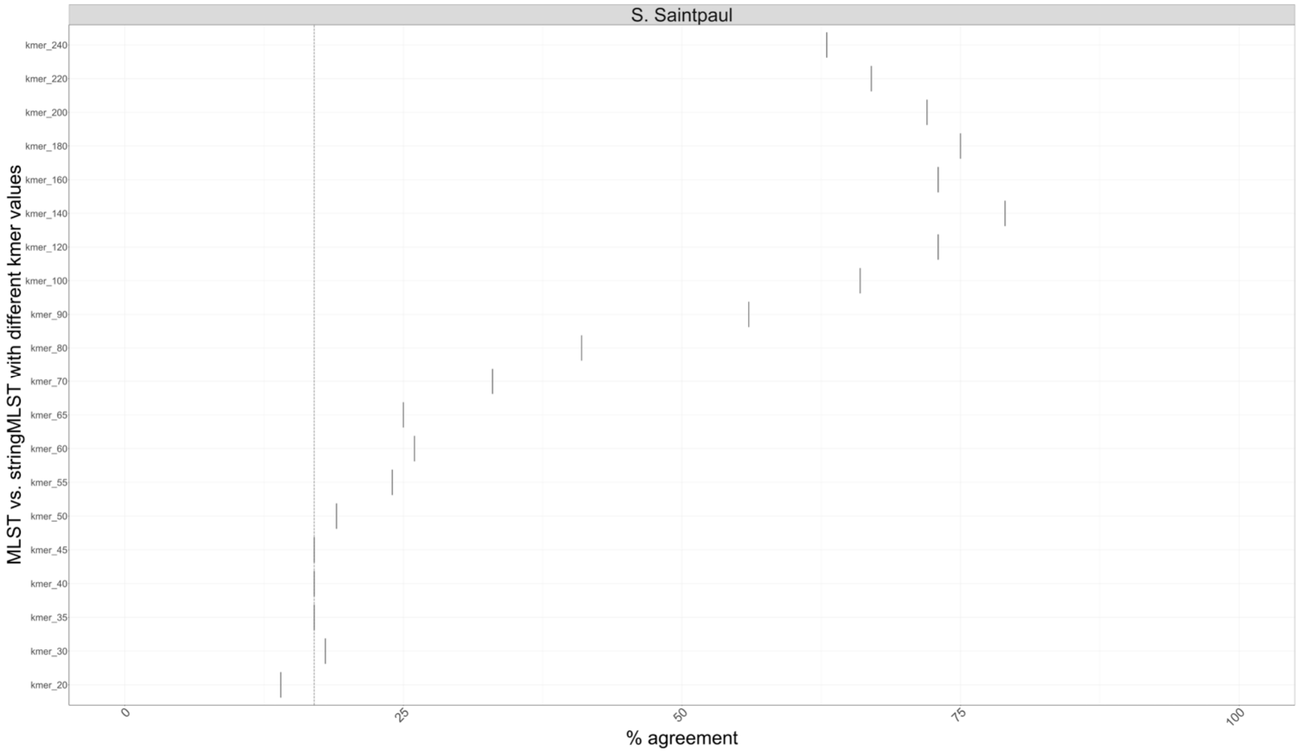
S. Saintpaul
MLST vs. stringMLST with different kmer values
kmer_240
kmer_220
kmer_200
kmer_180
kmer_160
kmer_140
kmer_120
kmer_100
kmer_90
kmer_80
kmer_70
kmer_65
kmer_60
kmer_55
kmer_50
kmer_45
kmer_40
kmer_35
kmer_30
kmer_20
0
25
50
75
100
% agreement

E.

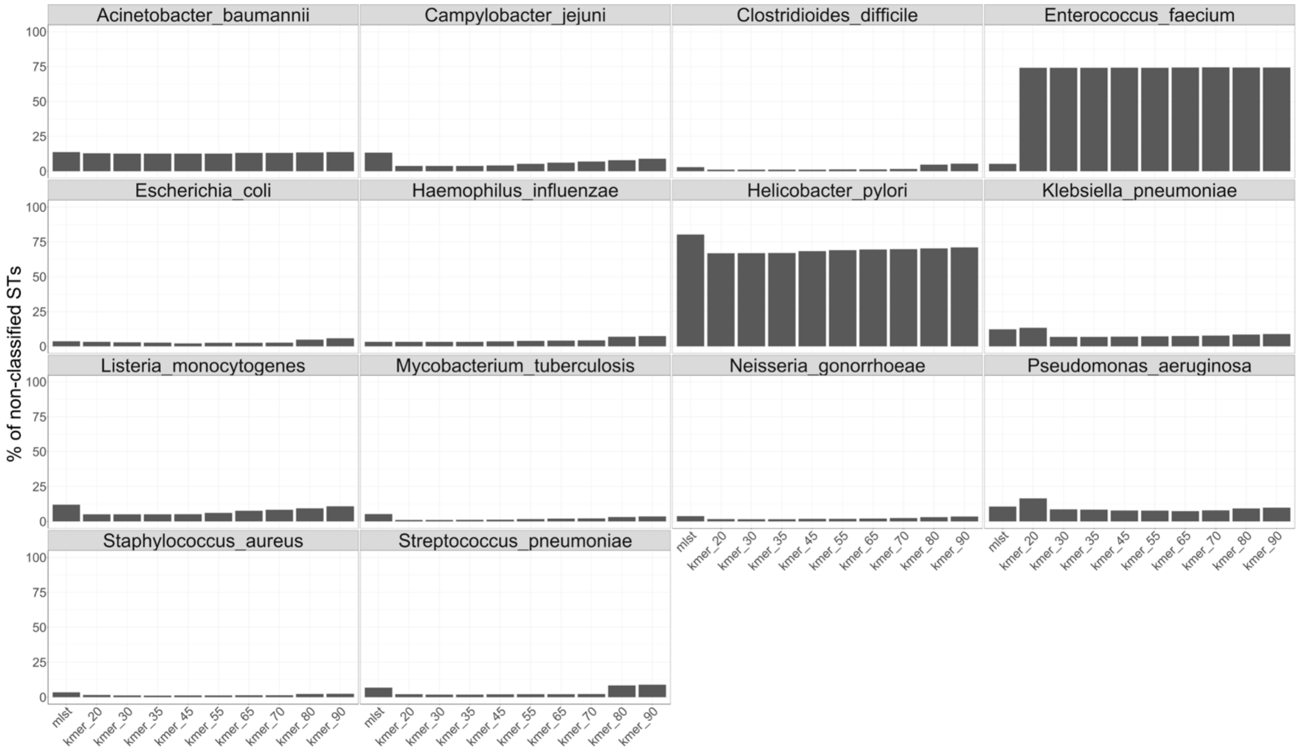
Acinetobacter_baumannii
Campylobacter_jejuni
Clostridioides_difficile
Enterococcus_faecium
Escherichia_coli
Haemophilus_influenzae
Helicobacter_pylori
Klebsiella_pneumoniae
Listeria_monocytogenes
Mycobacterium_tuberculosis
Neisseria_gonorrhoeae
Pseudomonas_aeruginosa
Staphylococcus_aureus
Streptococcus_pneumoniae
% of non-classified STs
0
25
50
75
100
mlst
kmer_20
kmer_30
kmer_35
kmer_45
kmer_55
kmer_65
kmer_70
kmer_80
kmer_90

F.

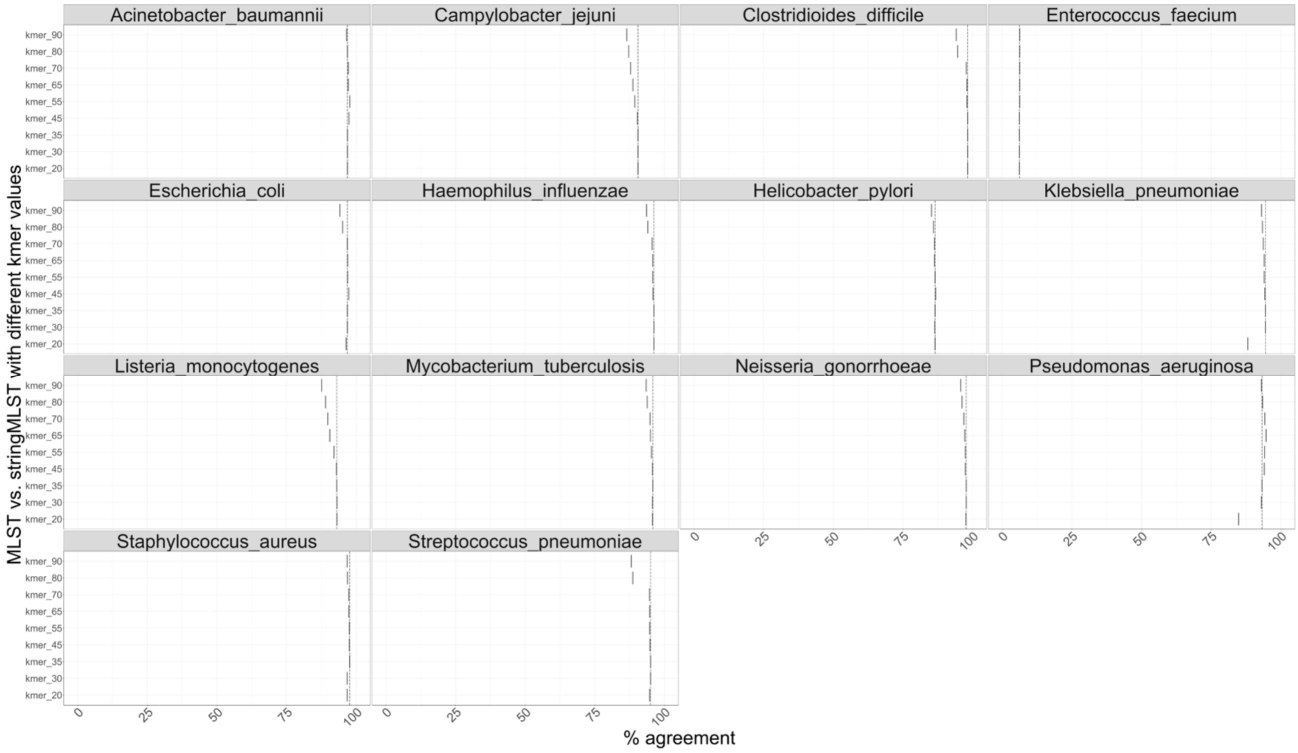
Acinetobacter_baumannii
Campylobacter_jejuni
Clostridioides_difficile
Enterococcus_faecium
Escherichia_coli
Haemophilus_influenzae
Helicobacter_pylori
Klebsiella_pneumoniae
Listeria_monocytogenes
Mycobacterium_tuberculosis
Neisseria_gonorrhoeae
Pseudomonas_aeruginosa
Staphylococcus_aureus
Streptococcus_pneumoniae
MLST vs. stringMLST with different kmer values
kmer_90
kmer_80
kmer_70
kmer_65
kmer_55
kmer_45
kmer_35
kmer_30
kmer_20
0
25
50
75
100
% agreement

G.

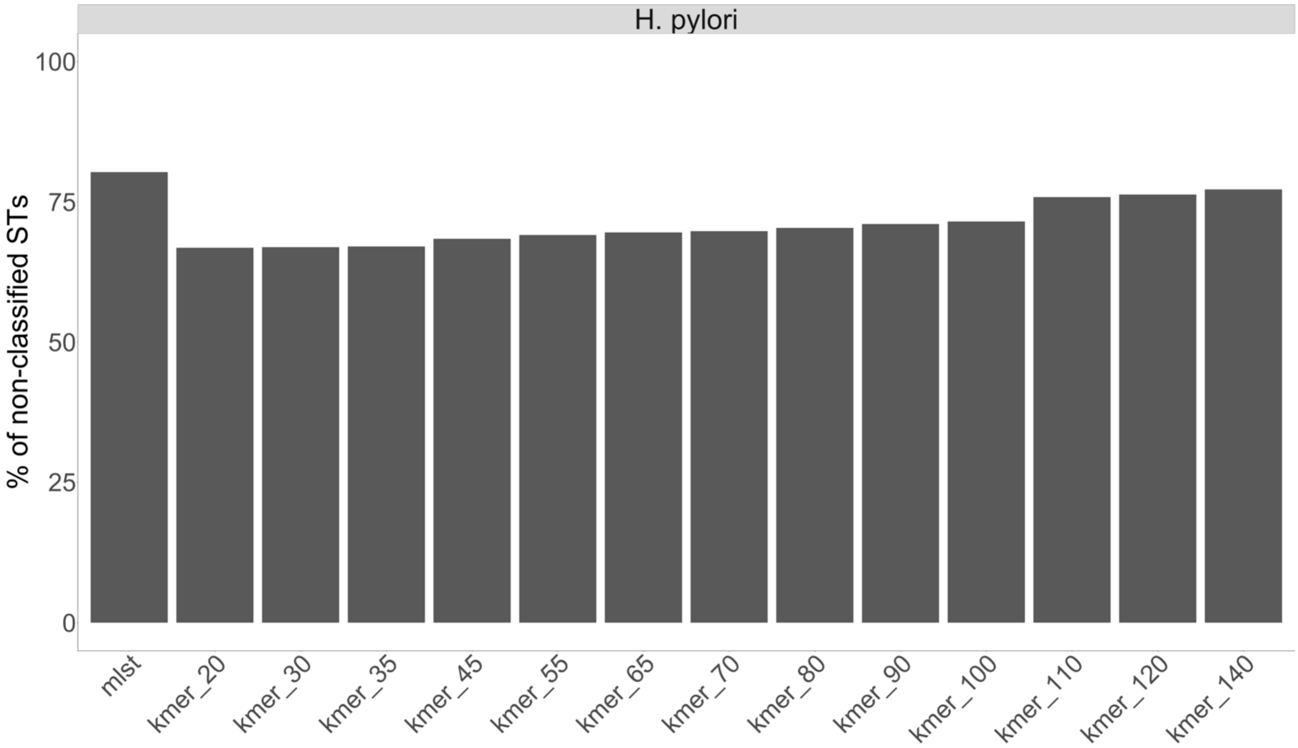
H. pylori
% of non-classified STs
100
75
50
25
0
mlst
kmer_20
kmer_30
kmer_35
kmer_45
kmer_55
kmer_65
kmer_70
kmer_80
kmer_90
kmer_100
kmer_110
kmer_120
kmer_140

H.

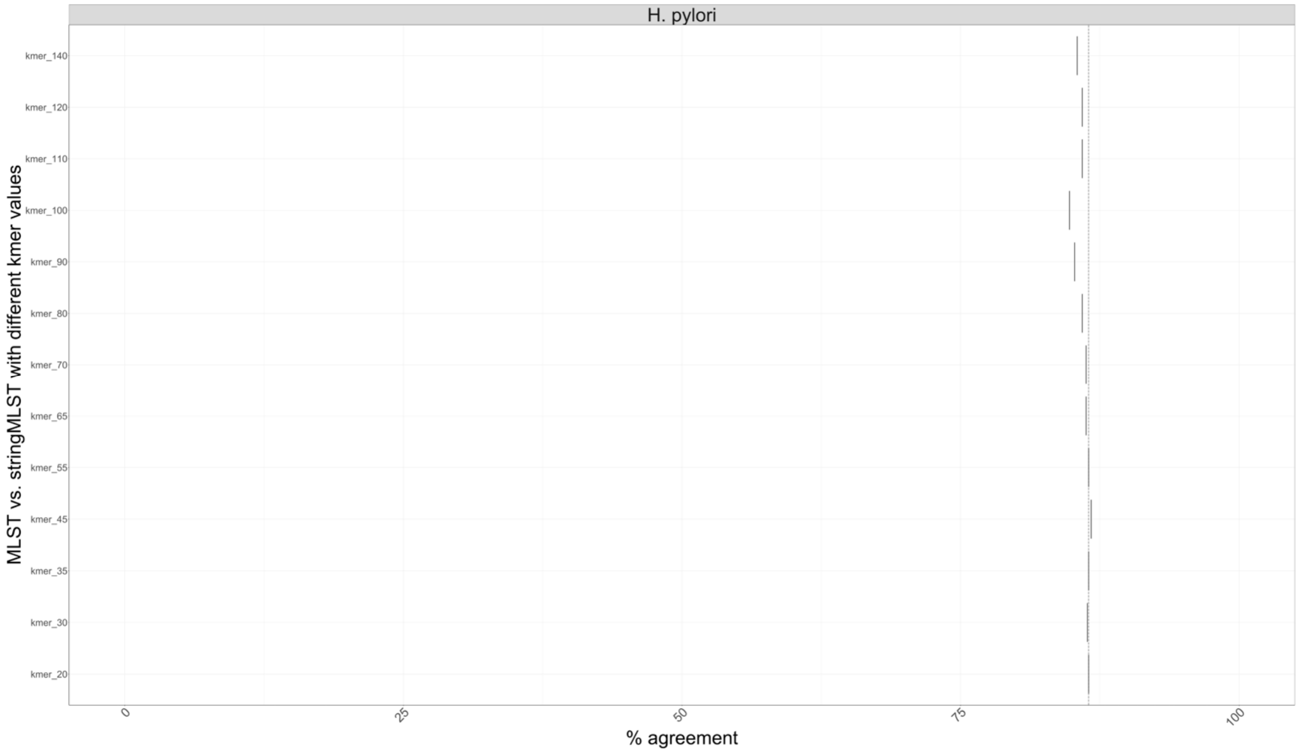
H. pylori
MLST vs. stringMLST with different kmer values
kmer_140
kmer_120
kmer_110
kmer_100
kmer_90
kmer_80
kmer_70
kmer_65
kmer_55
kmer_45
kmer_35
kmer_30
kmer_20
0
25
50
75
100
% agreement

I.

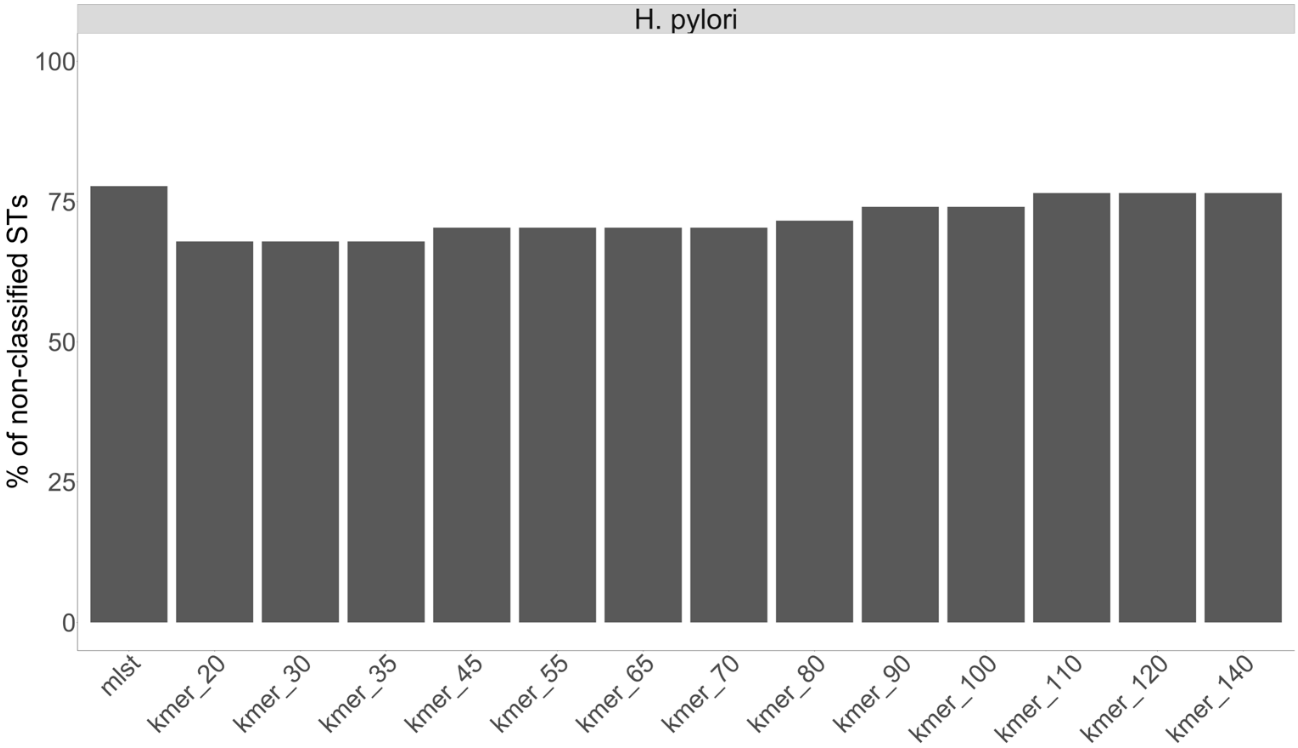
H. pylori
% of non-classified STs
100
75
50
25
0
mlst
kmer_20
kmer_30
kmer_35
kmer_45
kmer_55
kmer_65
kmer_70
kmer_80
kmer_90
kmer_100
kmer_110
kmer_120
kmer_140

J.

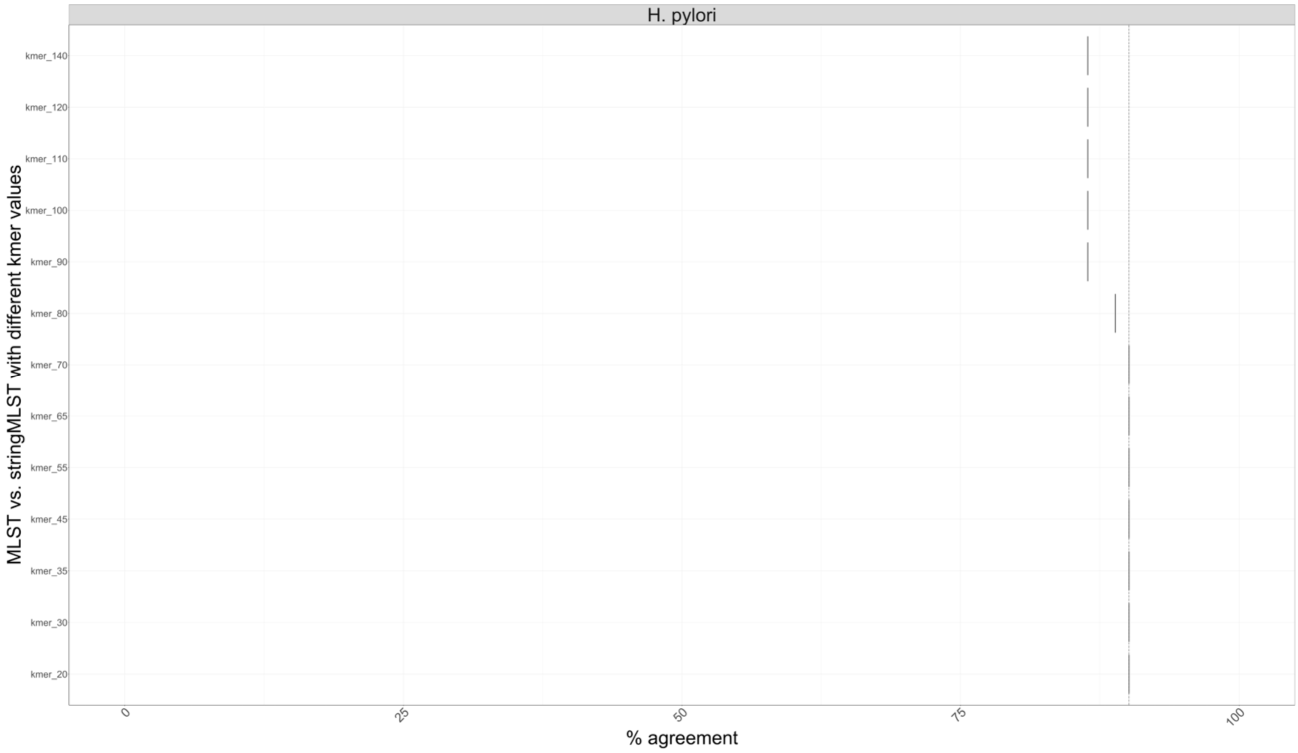
H. pylori
MLST vs. stringMLST with different kmer values
kmer_140
kmer_120
kmer_110
kmer_100
kmer_90
kmer_80
kmer_70
kmer_65
kmer_55
kmer_45
kmer_35
kmer_30
kmer_20
0
25
50
75
100
% agreement

K.

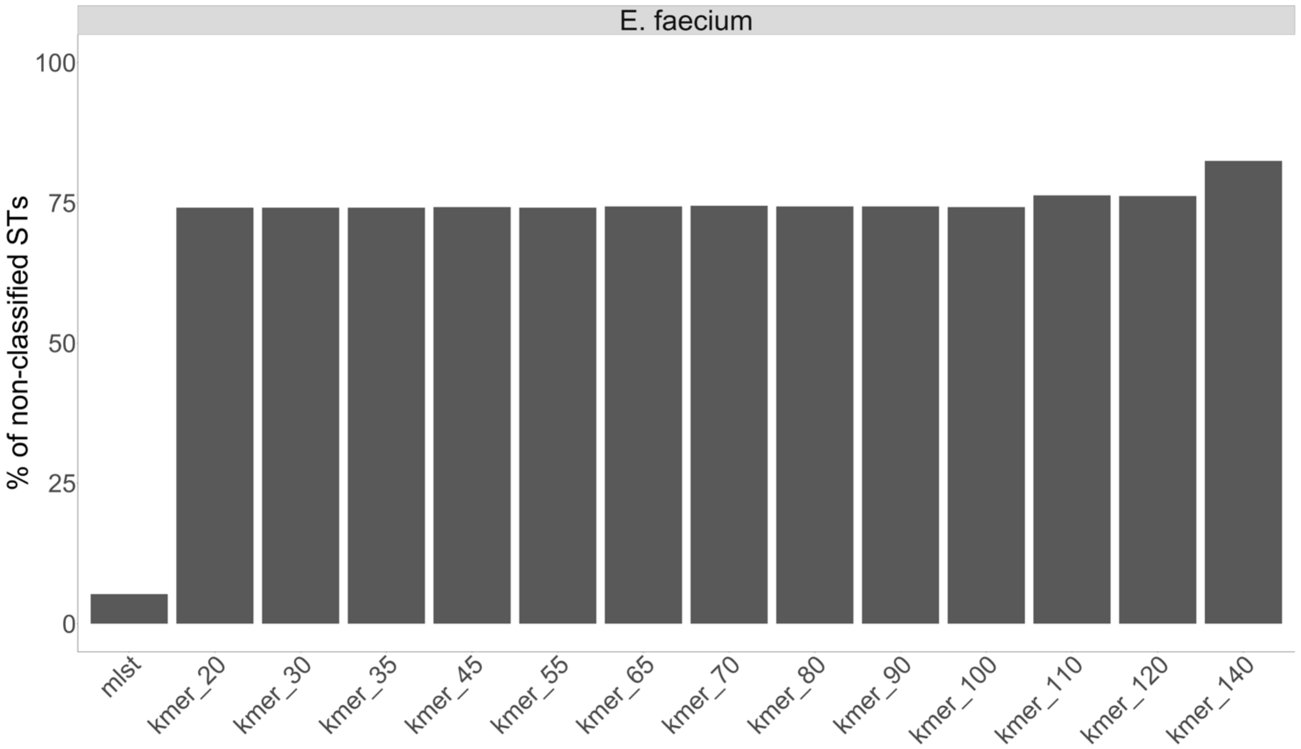
E. faecium
% of non-classified STs
100
75
50
25
0
mlst
kmer_20
kmer_30
kmer_35
kmer_45
kmer_55
kmer_65
kmer_70
kmer_80
kmer_90
kmer_100
kmer_110
kmer_120
kmer_140

L.

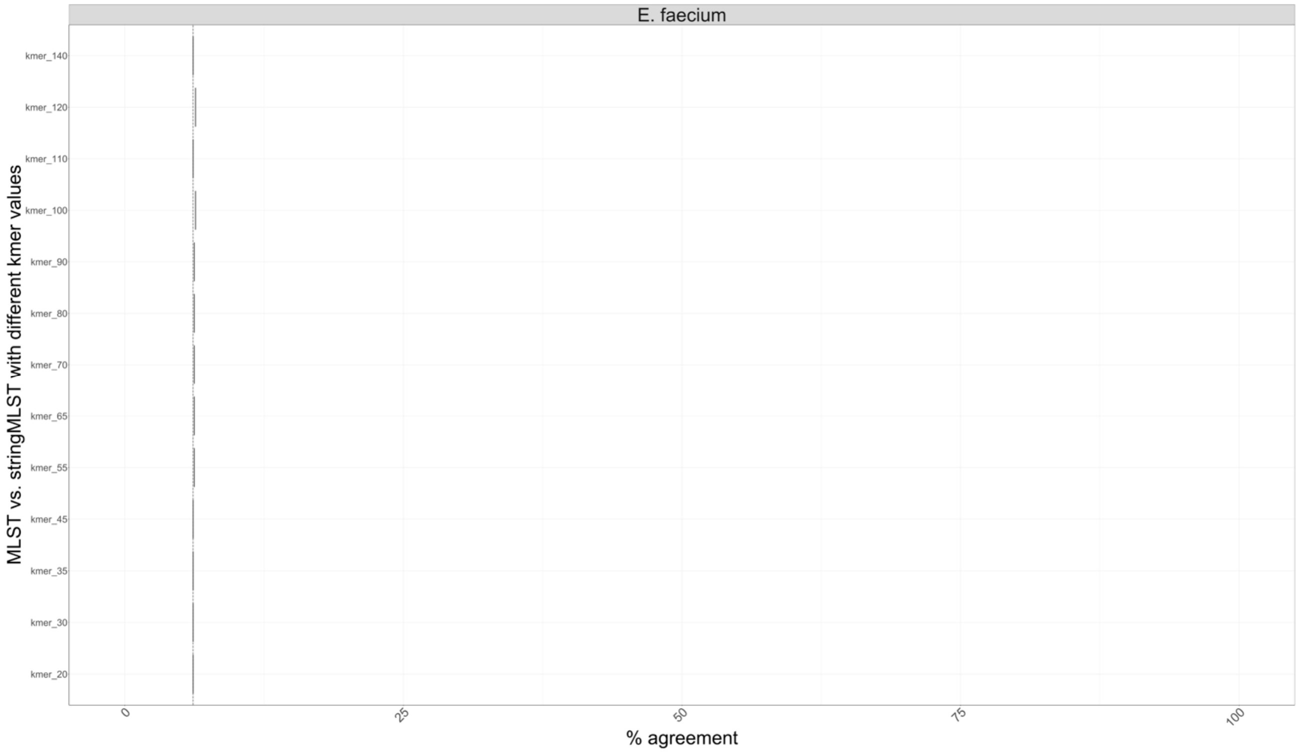
E. faecium
MLST vs. stringMLST with different kmer values
kmer_140
kmer_120
kmer_110
kmer_100
kmer_90
kmer_80
kmer_70
kmer_65
kmer_55
kmer_45
kmer_35
kmer_30
kmer_20
0
25
50
75
100
% agreement

M.

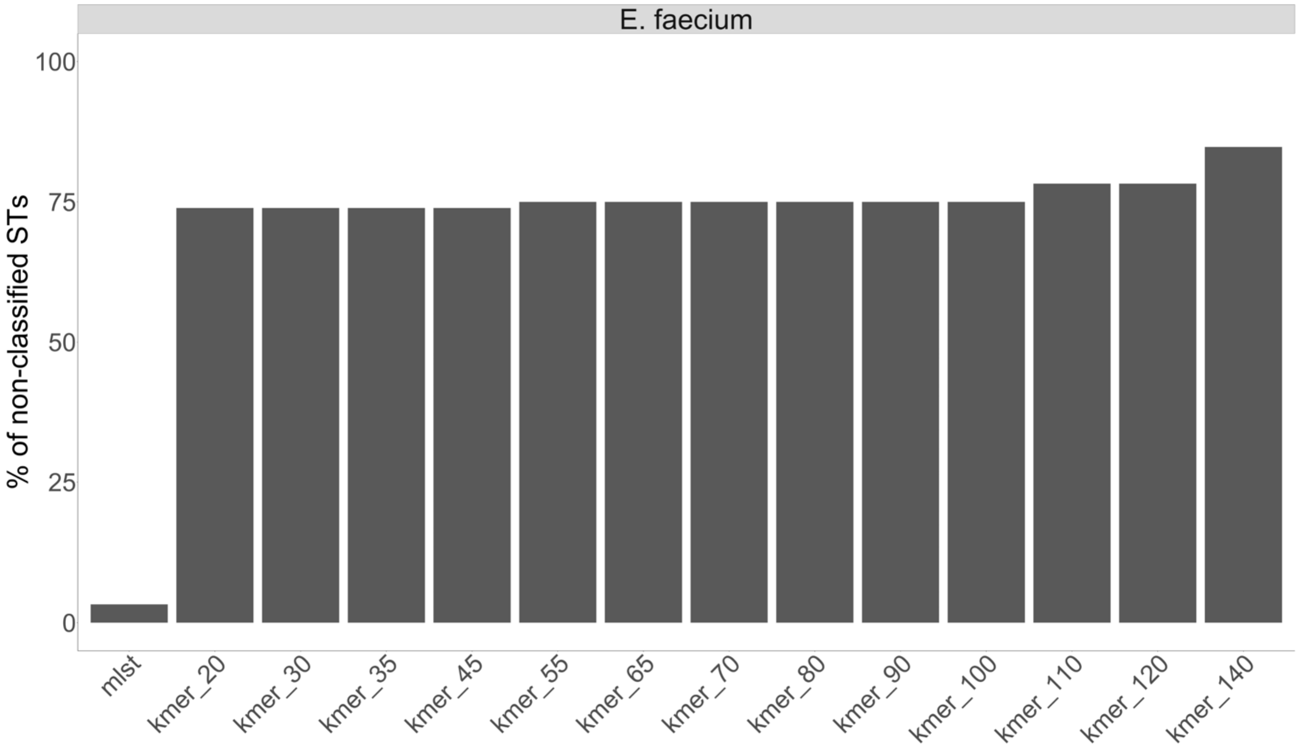
E. faecium
% of non-classified STs
100
75
50
25
0
mlst
kmer_20
kmer_30
kmer_35
kmer_45
kmer_55
kmer_65
kmer_70
kmer_80
kmer_90
kmer_100
kmer_110
kmer_120
kmer_140

N.

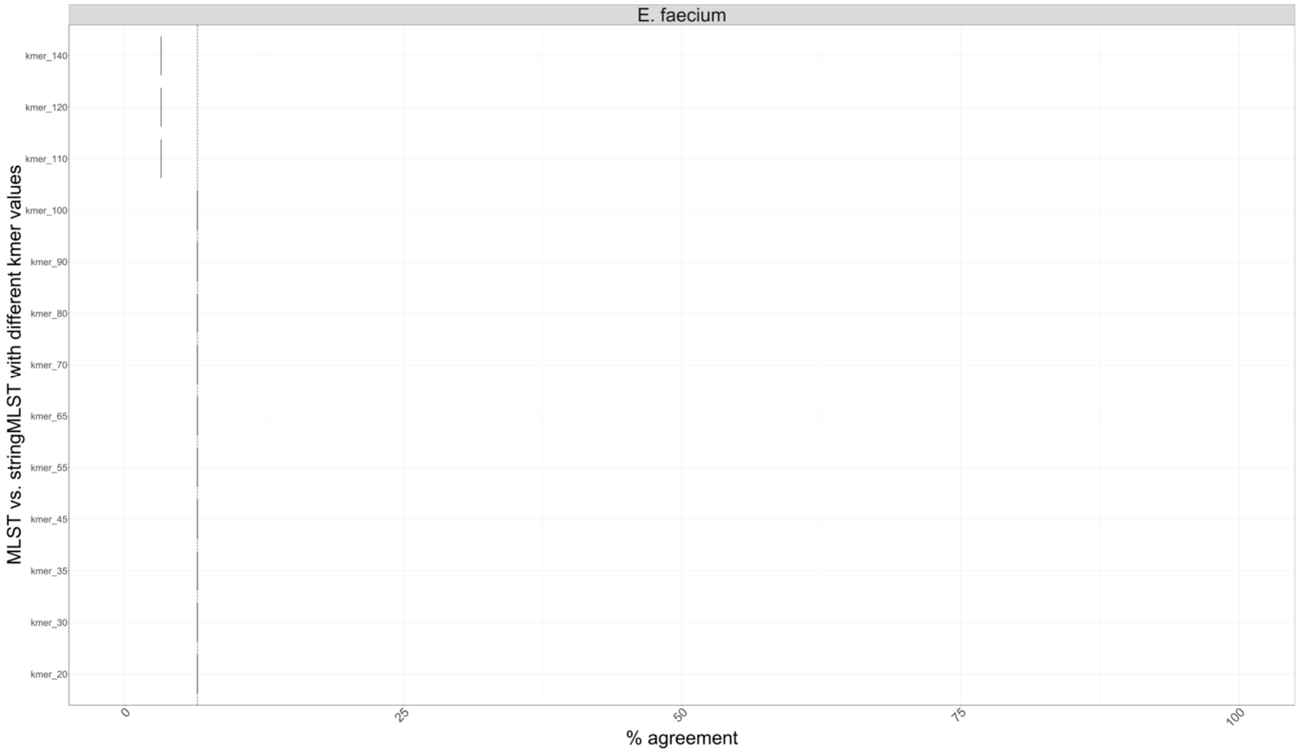
E. faecium
MLST vs. stringMLST with different kmer values
kmer_140
kmer_120
kmer_110
kmer_100
kmer_90
kmer_80
kmer_70
kmer_65
kmer_55
kmer_45
kmer_35
kmer_30
kmer_20
0
25
50
75
100
% agreement

O.

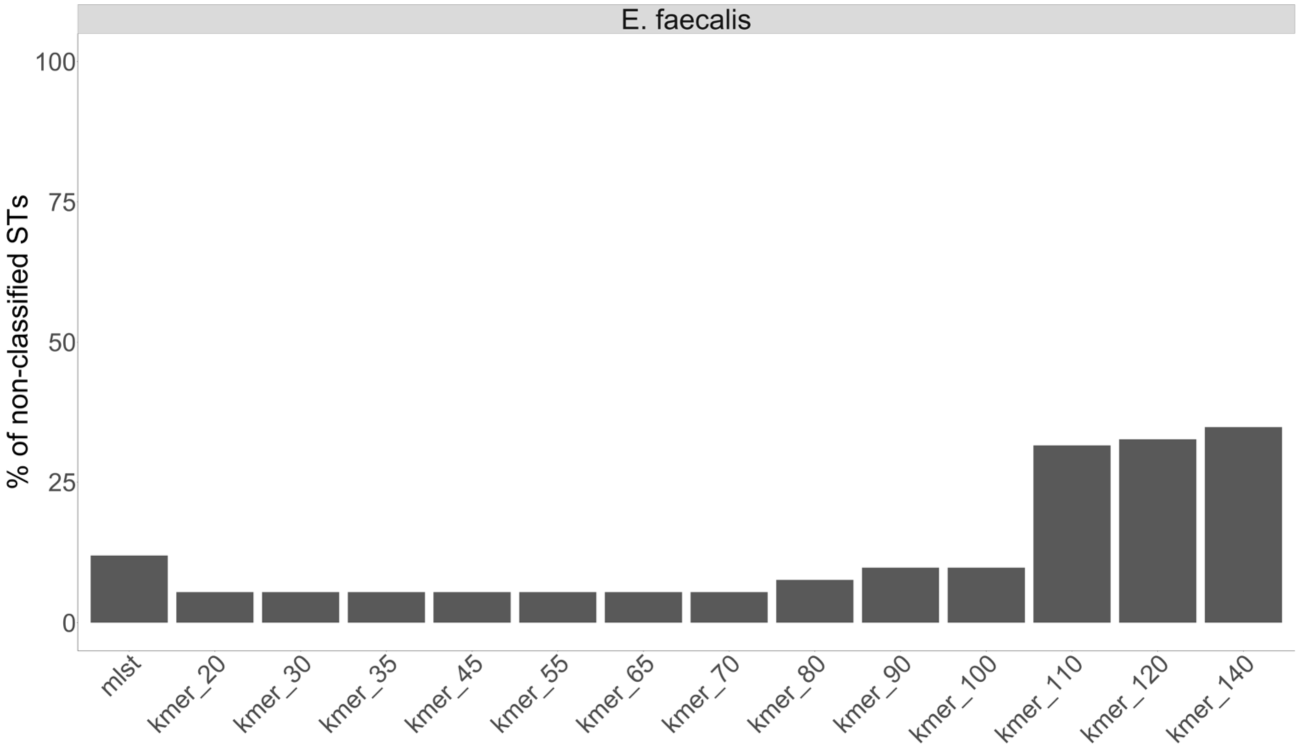
E. faecalis
% of non-classified STs
100
75
50
25
0
mlst
kmer_20
kmer_30
kmer_35
kmer_45
kmer_55
kmer_65
kmer_70
kmer_80
kmer_90
kmer_100
kmer_110
kmer_120
kmer_140

P.

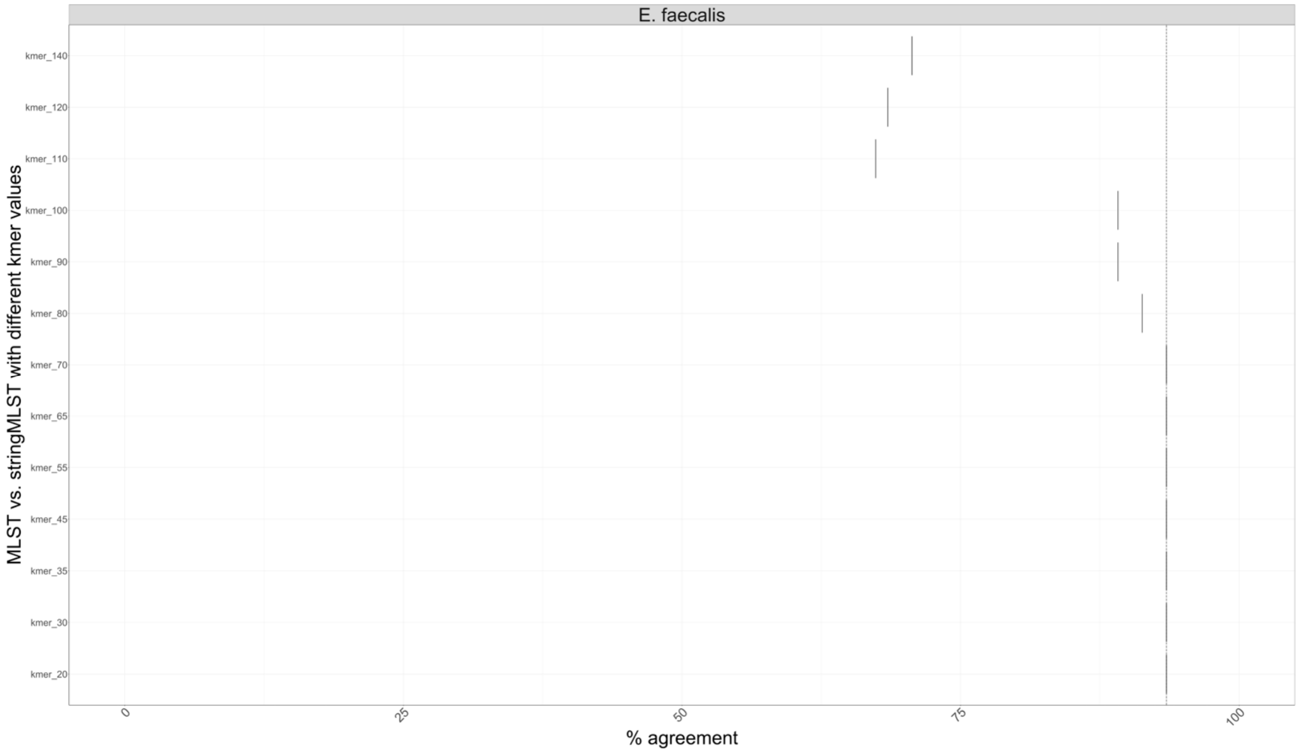
E. faecalis
MLST vs. stringMLST with different kmer values
kmer_140
kmer_120
kmer_110
kmer_100
kmer_90
kmer_80
kmer_70
kmer_65
kmer_55
kmer_45
kmer_35
kmer_30
kmer_20
0
25
50
75
100
% agreement

Figure S16. Comparison of the statistical performance metrics between mlst and stringMLST with different k-mer lengths across *Salmonella enterica* serovars and other phylogenetic divergent bacterial pathogens.

For the twenty-three *Salmonella enterica* serovars (*S*. Agona, *S*. Anatum, *S*. Braenderup, *S*. Derby, *S*. Dublin, *S*. Enteritidis, *S*. Hadar, *S*. Heidelberg, *S*. Infantis, *S*. Javiana, *S*. Johannesburg, *S*. Kentucky, *S*. Mbandaka, *S*. Montevideo, *S*. Muenchen, *S*. Newport, *S*. Oranienburg, *S*. Poona, *S*. Saintpaul, *S*. Schwarzengrund, *S*. Senftenberg, *S*. Thompson, and *S*. Typhimurium), we randomly chose and downloaded 100 paired-end reads from NCBI-SRA. Next, for each dataset we ran mlst and stringMLST with k-mer lengths ranging from 20, 30, 35, 40, 45, 50, 55, 60, 65, 70, 80, 90. For the fourteen bacterial pathogens (*Acinetobacter baumannii, Clostridioides difficile, Enterococcus faecium, Escherichia coli, Haemophilus influenzae, Helicobacter pylori, Klebsiella pneumoniae, Mycobacterium tuberculosis, Neisseria gonorrhoeae, Pseudomonas aeruginosa, Streptococcus pneumoniae, Campylobacter jejuni, Listeria monocytogenes,* and *Staphylococcus aureus),* we randomly chose and downloaded 1,000 paired-end reads from NCBI-SRA. Next, for each dataset we ran mlst and stringMLST with k-mer lengths ranging from 20, 30, 35, 45, 55, 65, 70, 80, 90. (A) Percentage of non-classified STs (ST calls that returned missing/blank values) across tweenty-three *S. enterica* serovars for mlst and stringMLST using an array of k-mer lengths (20, 30, 35, 40, 45, 50, 55, 60, 65, 70, 80, 90); (B) Percentage of agreement (concordance) between programs ("good" or "bad" ST calls that matched between mlst and stringMLST for different k-mer lengths) across all twenty-three S. *enterica* serovars; (C) Percentage of non-classified STs for *S*. Saintpaul using mlst and longer k-mer lenghts with stringMLST; (D) Percentage of agreement between programs for *S*. Saintpaul using mlst and longer k-mer lengths with stringMLST; (E) Percentage of non-classified STs (ST calls that returned missing/blank values) across fourteen phylogenetic divergent bacterial pathogens for mlst and stringMLST with range of k-mer lengths (20, 30, 35, 45, 55, 65, 70, 80, 90); (F) Percentage of agreement between programs ("good" or "bad" ST calls that matched between mlst and stringMLST for different k-mer lengths) across all fourteen phylogenetic divergent bacterial pathogens; (G) Percentage of non-classified STs for *H. pylori* (dataset of 1,000 genomes) using mlst and longer k-mer lengths with stringMLST; (H) Percentage of agreement between programs for *H. pylori* (dataset of 1,000 genomes) using mlst and lost k-mer lengths with stringMLST; (I) Percentage of non-classified STs for *H. pylori* (dataset of 100 genomes) using mlst and longer k-mer lengths with stringMLST; (J) Percentage of agreement between programs for *H. pylori* (dataset of 100 genomes) using mlst and lost k-mer lengths with stringMLST; (K) Percentage of non-classified STs for *E. faecium* (dataset of 1,000 genomes) using mlst and longer k-mer lengths with

stringMLST; (L) Percentage of agreement (concordance) between programs for *E. faecium* (dataset of 1,000 genomes) using mlst and longer k-mer lengths with stringMLST; (M) Percentage of non-classified STs for *E. faecium* (dataset of 100 genomes) using mlst and longer k-mer lengths with stringMLST; (N) Percentage of agreement (concordance) between programs for *E. faecium* (dataset of 100 genomes) using mlst and longer k-mer lengths with stringMLST; (O) Percentage of non-classified STs for *Enterococcus faecalis* (dataset of 100 genomes) using mlst and longer k-mer lenghts with stringMLST; (P) Percentage of agreement (concordance) between programs for *E. faecalis* (dataset of 100 genomes) using mlst and longer k-mer lengths with stringMLST.
