# Supplementary material for: Systems-Based Approach for Optimization of Assembly-Free Bacterial MLST Mapping
Source: Life (Basel). 2022 Apr 30;12(5):670. doi: 10.3390/life12050670 (PMC9147691; doi:10.3390/life12050670)
Supplement: Supplementary file 1 [file life-12-00670-s001.zip › Figure S17.pdf]

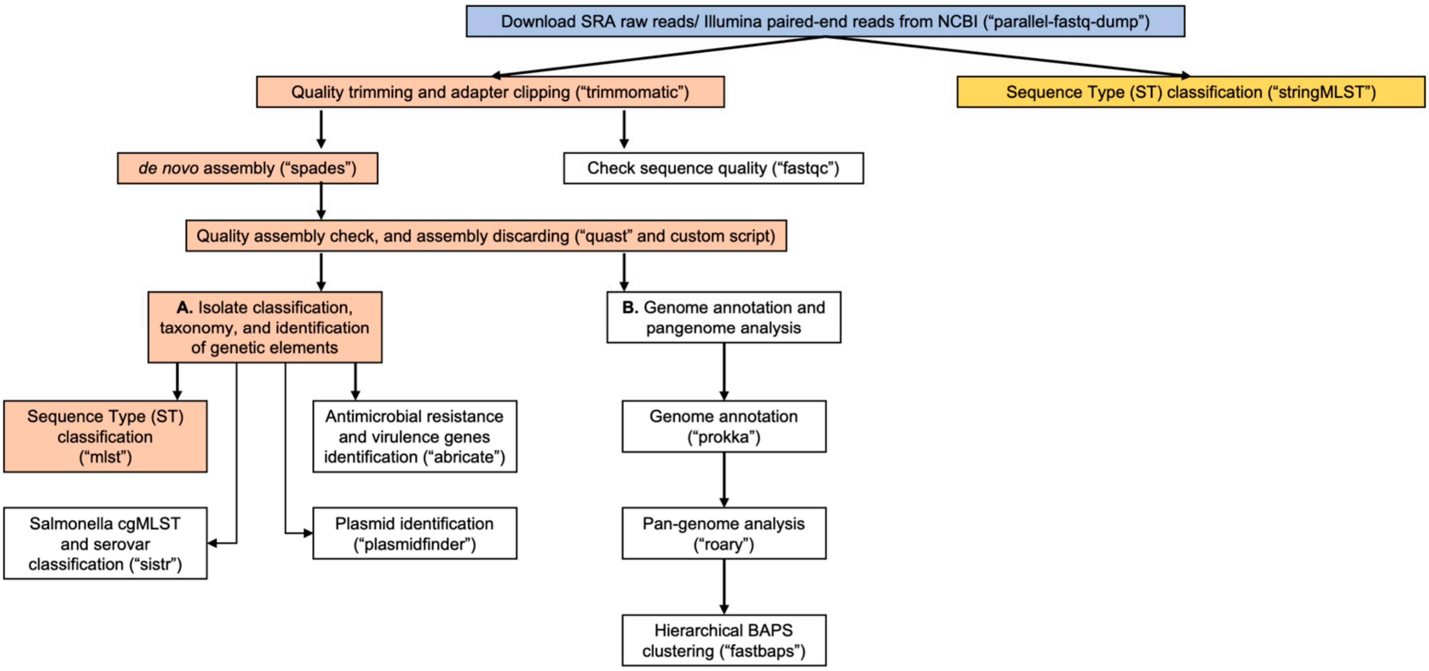


**Figure S17**. ProkEvo's computational workflow with both mlst and stringMLST included for ST-based classification.
Top-down flow of tasks for the ProkEvo computational pipeline. The squares represent the steps, while the bioinformatics tool used for each step is shown in brackets. The sub-workflow on the left includes all the steps of the current ProkEvo platform, for which the steps needed for obtaining ST classifications with mlst are colored in orange. The sub-workflow on the right includes all the steps of the alternative path in the ProkEvo platform, where stringMLST was integrated for ST-based classification.
