# Supplementary material for: Systems-Based Approach for Optimization of Assembly-Free Bacterial MLST Mapping
Source: Life (Basel). 2022 Apr 30;12(5):670. doi: 10.3390/life12050670 (PMC9147691; doi:10.3390/life12050670)
Supplement: Supplementary file 1 [file life-12-00670-s001.zip › Figure S18.pdf]

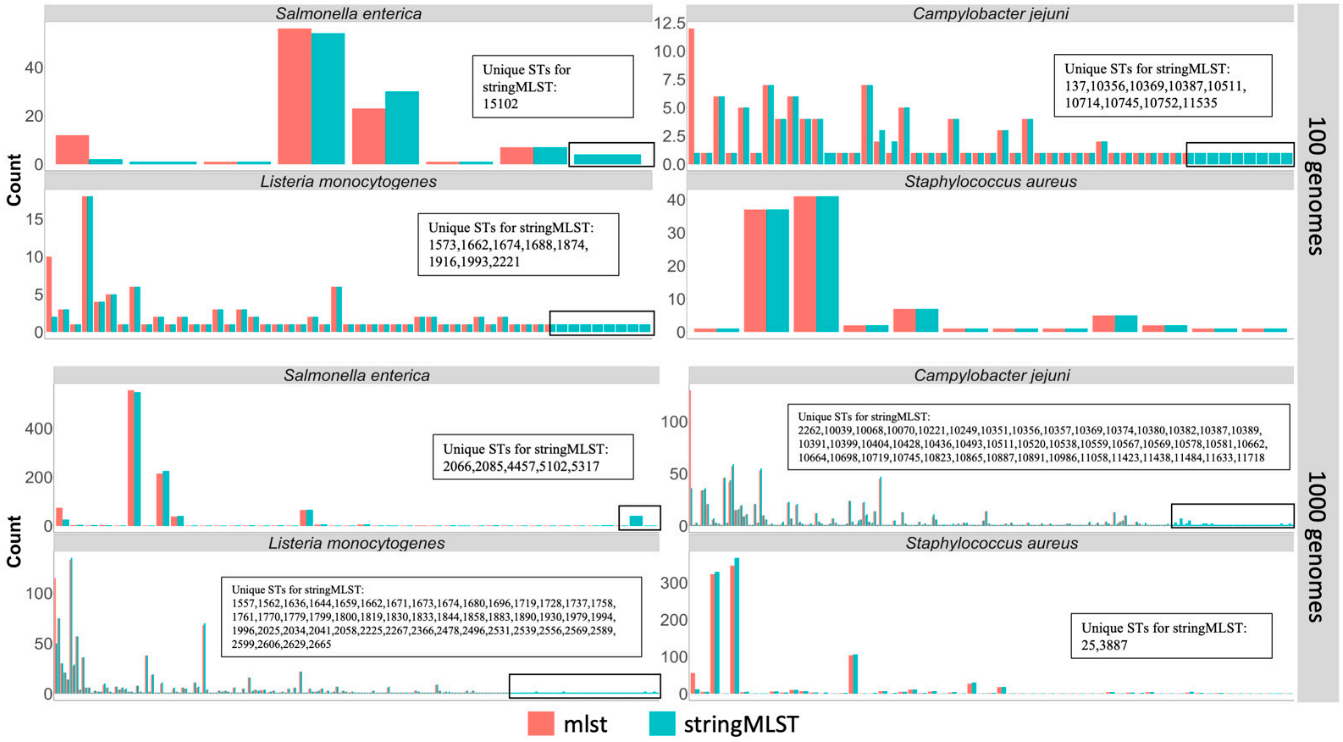

Figure S18. Frequency of ST lineages across bacterial species and programs using two random sample sizes.

Pairwise frequency-based (y-axis = count) distribution of unique ST lineages identified by either mlst or stringMLST across bacterial species using a random sampling of 100 or 1,000 genomes. Each couple of pairwise bar counts (both programs) represents a unique ST number or lineage. Highlighted with black rectangles are the ST lineages uniquely found by the stringMLST program. Moreover, the STs unique for stringMLST are listed in a text box in each plot.
