# Supplementary material for: Systems-Based Approach for Optimization of Assembly-Free Bacterial MLST Mapping
Source: Life (Basel). 2022 Apr 30;12(5):670. doi: 10.3390/life12050670 (PMC9147691; doi:10.3390/life12050670)
Supplement: Supplementary file 1 [file life-12-00670-s001.zip › Figure S19.pdf]

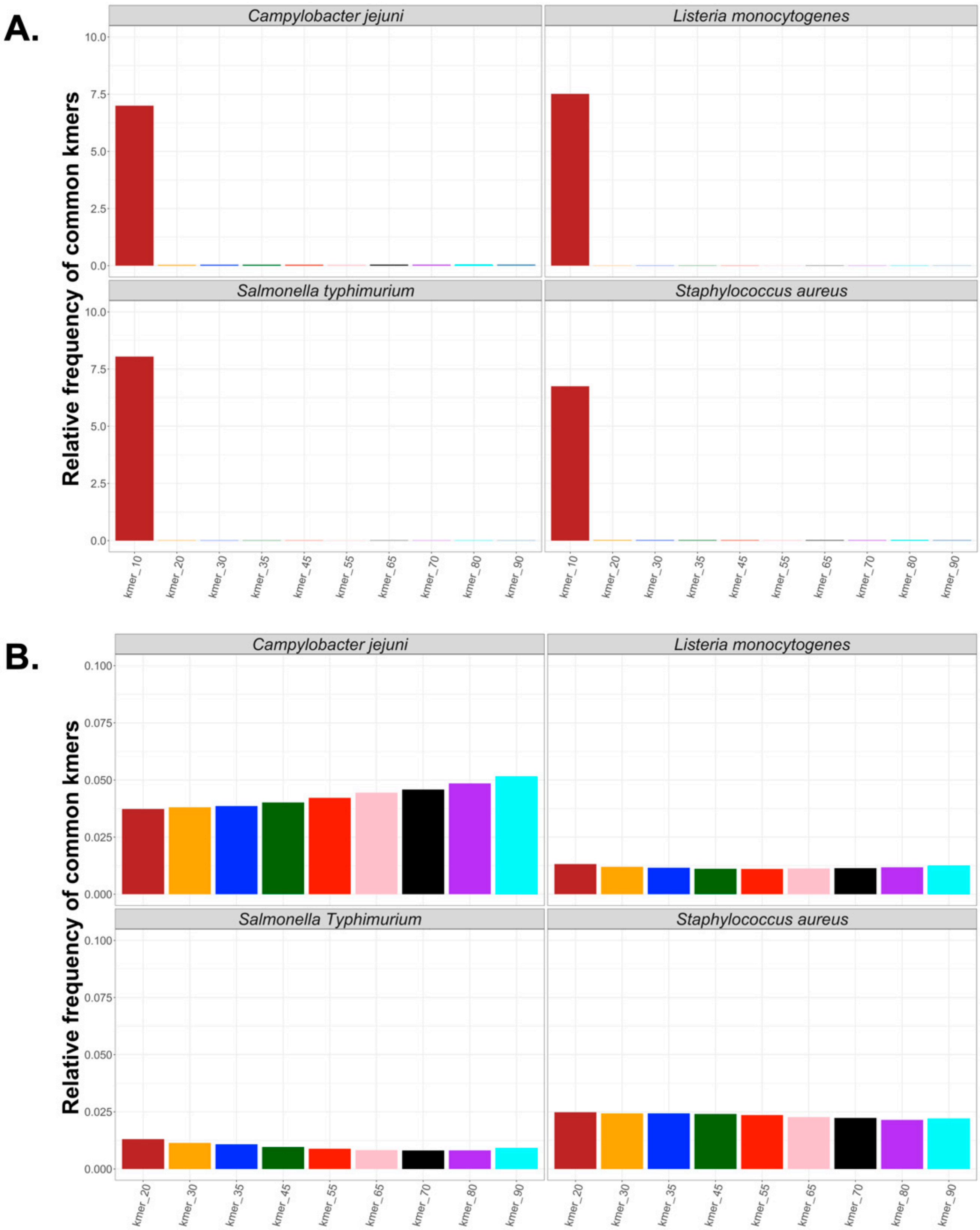
A.
Campylobacter jejuni
Listeria monocytogenes
Salmonella typhimurium
Staphylococcus aureus
Relative frequency of common kmers
0.0
2.5
5.0
7.5
10.0
kmer_10
kmer_20
kmer_30
kmer_35
kmer_45
kmer_55
kmer_65
kmer_70
kmer_80
kmer_90
B.
Campylobacter jejuni
Listeria monocytogenes
Salmonella Typhimurium
Staphylococcus aureus
Relative frequency of common kmers
0.000
0.025
0.050
0.075
0.100
kmer_20
kmer_30
kmer_35
kmer_45
kmer_55
kmer_65
kmer_70
kmer_80
kmer_90

Figure S19. Relative frequency of common k-mers found in the raw reads and the stringMLST database.

Random 100 raw paired-end reads from the initial *C. jejuni*, *L. monocytogenes*, *S. aureus* and *S.* Typhimurium (major representative zoonotic serovar of *S. enterica*) datasets were selected, and DSK was used to count the occurrence of k-mers of lengths 10, 20, 30, 35, 45, 55, 65, 70, 80 and 90 respectively in the raw reads. (A) Next, for each database created with stringMLST, a file with the k-mer frequency for the used ST scheme was generated. Using the k-mers generated from the raw reads and the stringMLST database, a relative frequency of the common k-mers was calculated. (B) Due to the large range of frequency-based values between k-mer 10 and the remaining k-mer lengths, the k-mer 10 data were excluded from the plot.
