# Supplementary material for: Systems-Based Approach for Optimization of Assembly-Free Bacterial MLST Mapping
Source: Life (Basel). 2022 Apr 30;12(5):670. doi: 10.3390/life12050670 (PMC9147691; doi:10.3390/life12050670)
Supplement: Supplementary file 1 [file life-12-00670-s001.zip › Figure S2.pdf]

A.

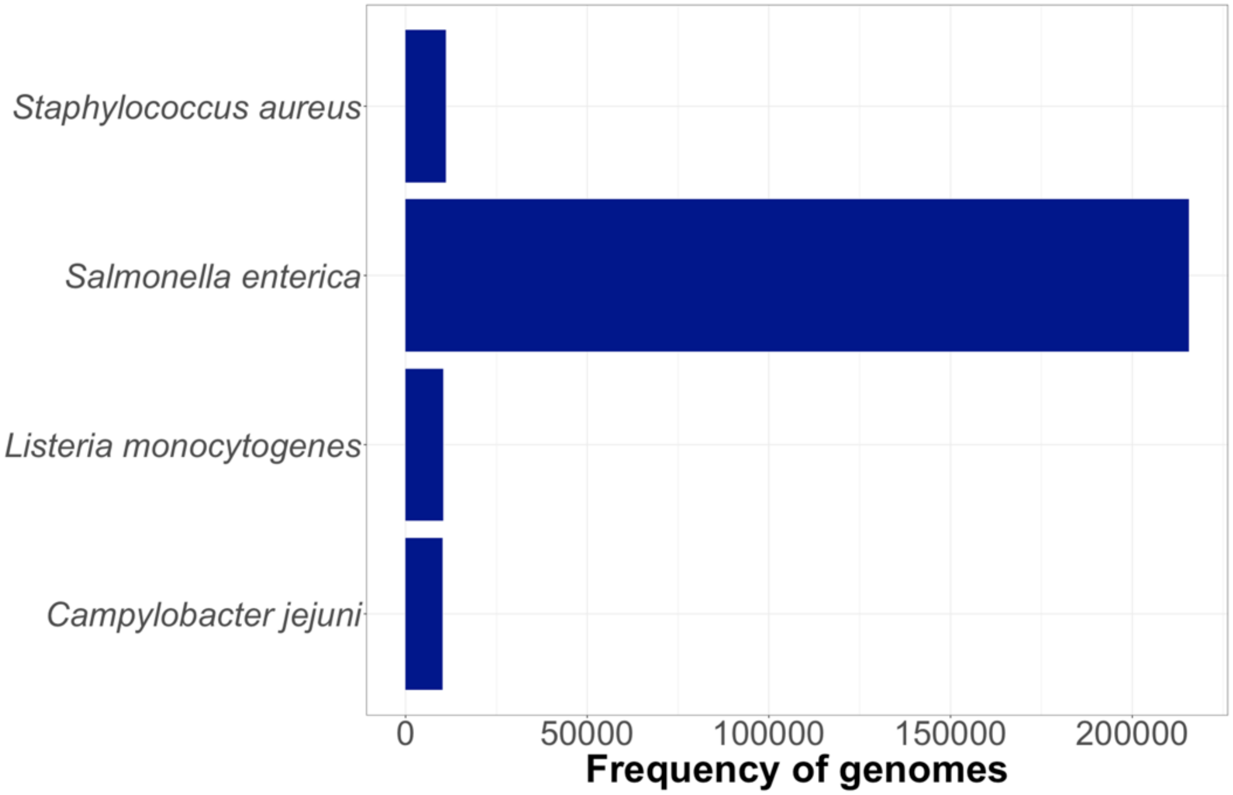

Staphylococcus aureus
Salmonella enterica
Listeria monocytogenes
Campylobacter jejuni
0
50000
100000
150000
200000
Frequency of genomes

B.

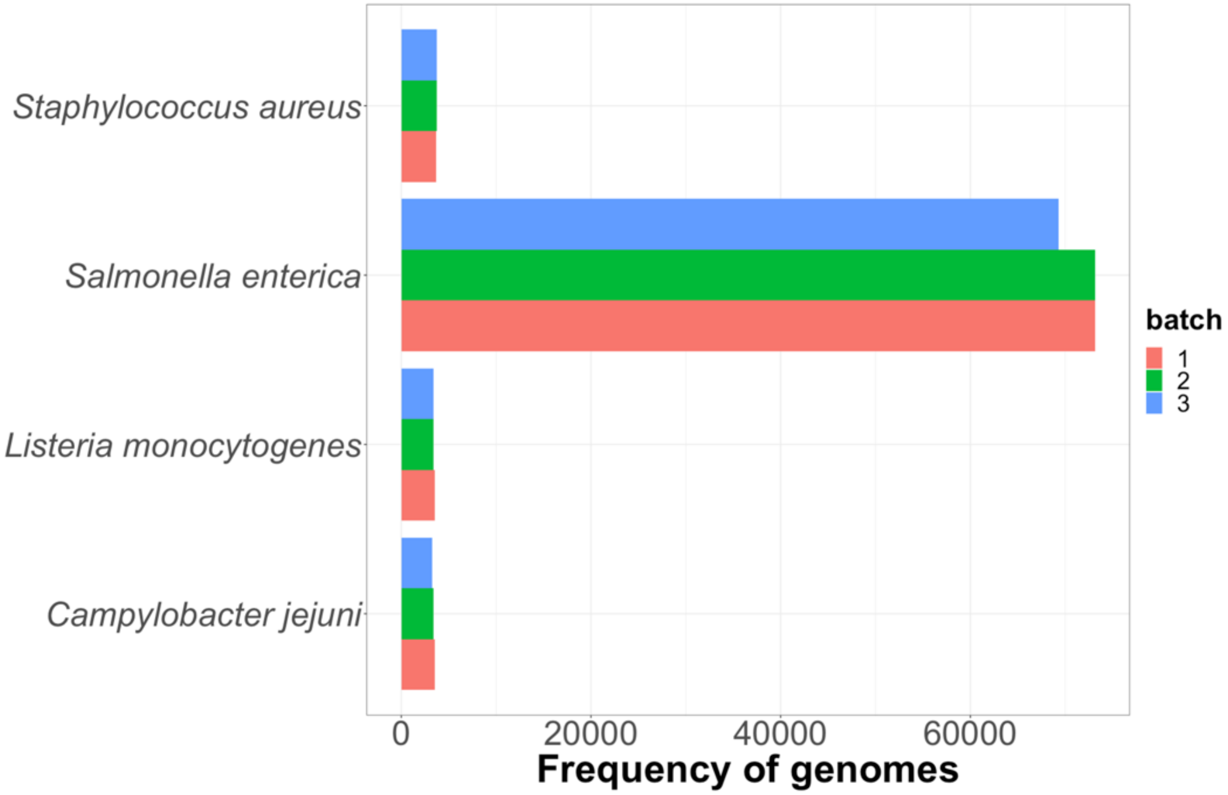

Staphylococcus aureus
Salmonella enterica
Listeria monocytogenes
Campylobacter jejuni
0
20000
40000
60000
Frequency of genomes
batch
1
2
3

C.

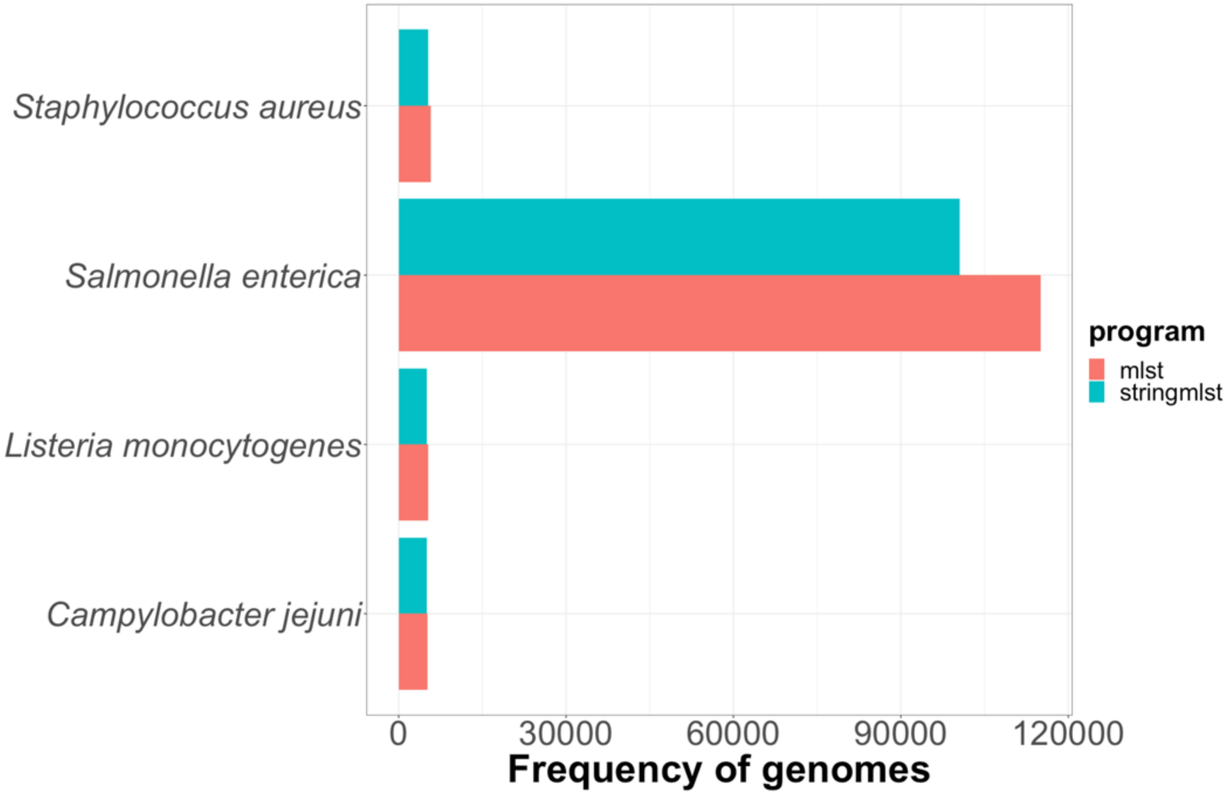
Staphylococcus aureus
Salmonella enterica
Listeria monocytogenes
Campylobacter jejuni
0
30000
60000
90000
120000
Frequency of genomes
program
mlst
stringmlst

D.

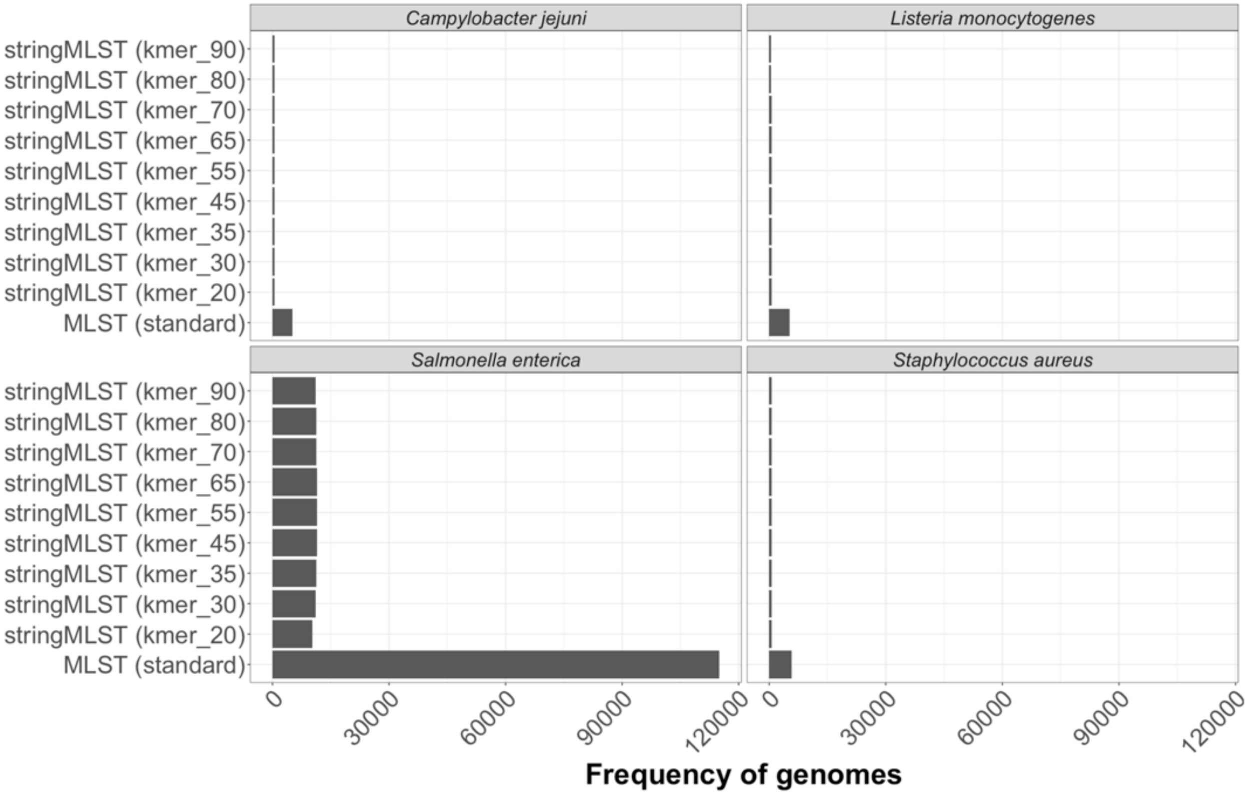
Campylobacter jejuni
Listeria monocytogenes
Salmonella enterica
Staphylococcus aureus
stringMLST (kmer_90)
stringMLST (kmer_80)
stringMLST (kmer_70)
stringMLST (kmer_65)
stringMLST (kmer_55)
stringMLST (kmer_45)
stringMLST (kmer_35)
stringMLST (kmer_30)
stringMLST (kmer_20)
MLST (standard)
0
30000
60000
90000
120000
Frequency of genomes

E.

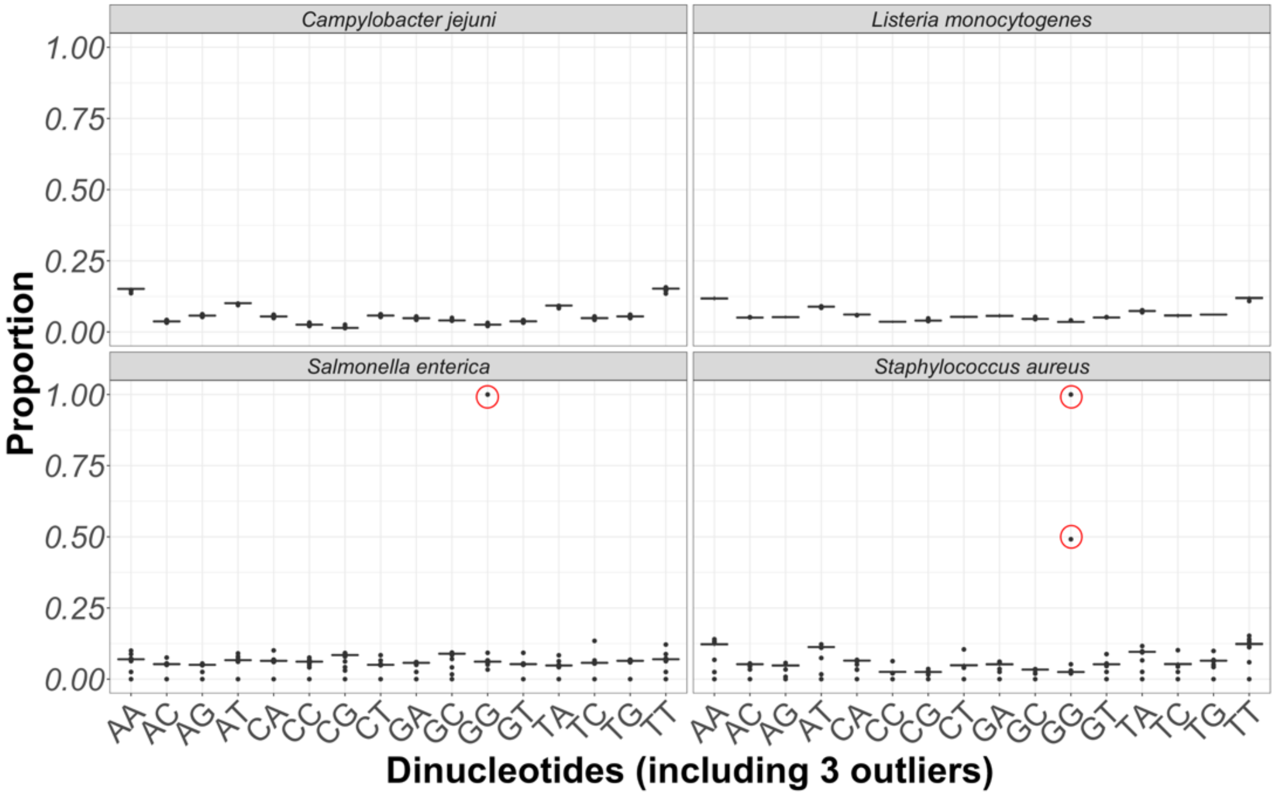

Campylobacter jejuni
Listeria monocytogenes
Salmonella enterica
Staphylococcus aureus
Proportion
1.00
0.75
0.50
0.25
0.00
AA AC AG AT CA CC CG CT GA GC GG GT TA TC TG TT
Dinucleotides (including 3 outliers)

F.

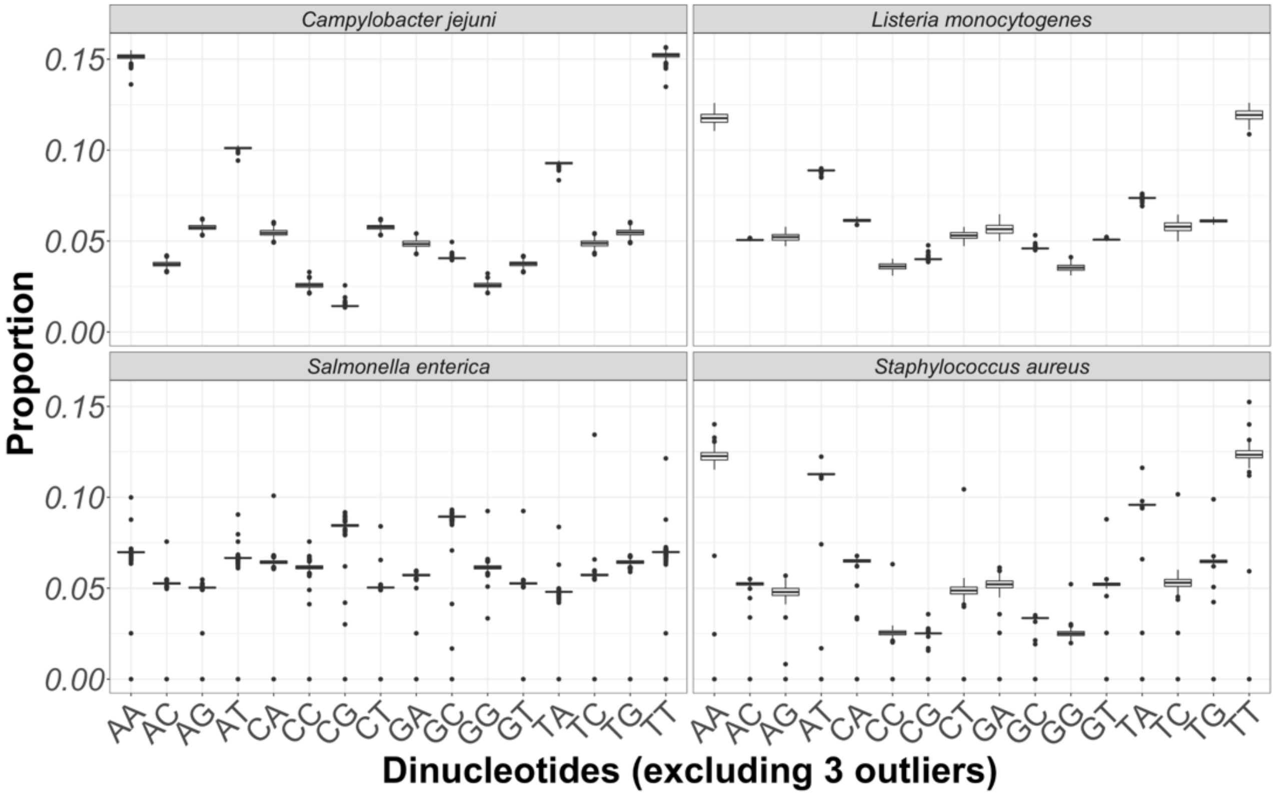

Campylobacter jejuni
Listeria monocytogenes
Salmonella enterica
Staphylococcus aureus
Proportion
0.15
0.10
0.05
0.00
AA AC AG AT CA CC CG CT GA GC GG GT TA TC TG TT
Dinucleotides (excluding 3 outliers)

G.

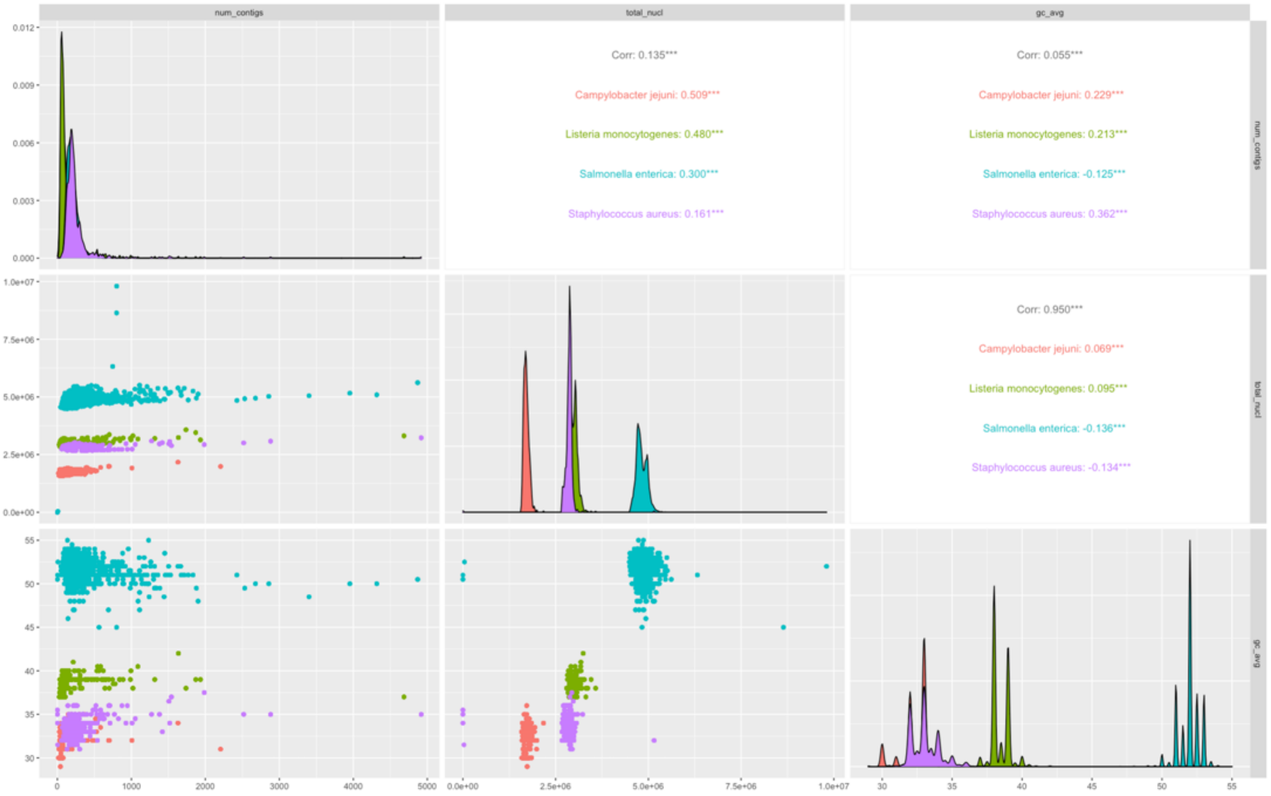

Figure S2. Summary statistics of the frequency of genomes, including the distribution of dinucleotides and bivariate associations between genome-intrinsic variables across all four bacterial species.

Frequency-based distribution of randomly selected genomes across bacterial species (A), including a stratification by batch (B), program (C), and further differentiation by k-mer length used by stringMLST (D). Proportion of all sixteen pairs of dinucleotides present in a bacterial genome, across species, with (E) or without outliers (red-circled data points) (F). (G) Bivariate association between genome-intrinsic variables across species with statistical significance measured by the Pearson correlation coefficient (Corr). Genome-intrinsic variables used were the total number of contigs (num_contigs), the total number of nucleotides per genome (assembly), and the GC% content per genome (gc_avg). (G) Asterisks refer to the degree of significance for the correlation coefficient, with *p*-value thresholds being: *$p < 0.05$, **$p \leq 0.01$, ***$p \leq 0.001$, ****$p \leq 0.0001$ and NS = not significant at $p \geq 0.05$. Across all figures A-G, data for *S. enterica* Subspecies *enterica* (*S. enterica*) contained an even proportion of genomes across twenty serovars.
