# Supplementary material for: Systems-Based Approach for Optimization of Assembly-Free Bacterial MLST Mapping
Source: Life (Basel). 2022 Apr 30;12(5):670. doi: 10.3390/life12050670 (PMC9147691; doi:10.3390/life12050670)
Supplement: Supplementary file 1 [file life-12-00670-s001.zip › Figure S20.pdf]

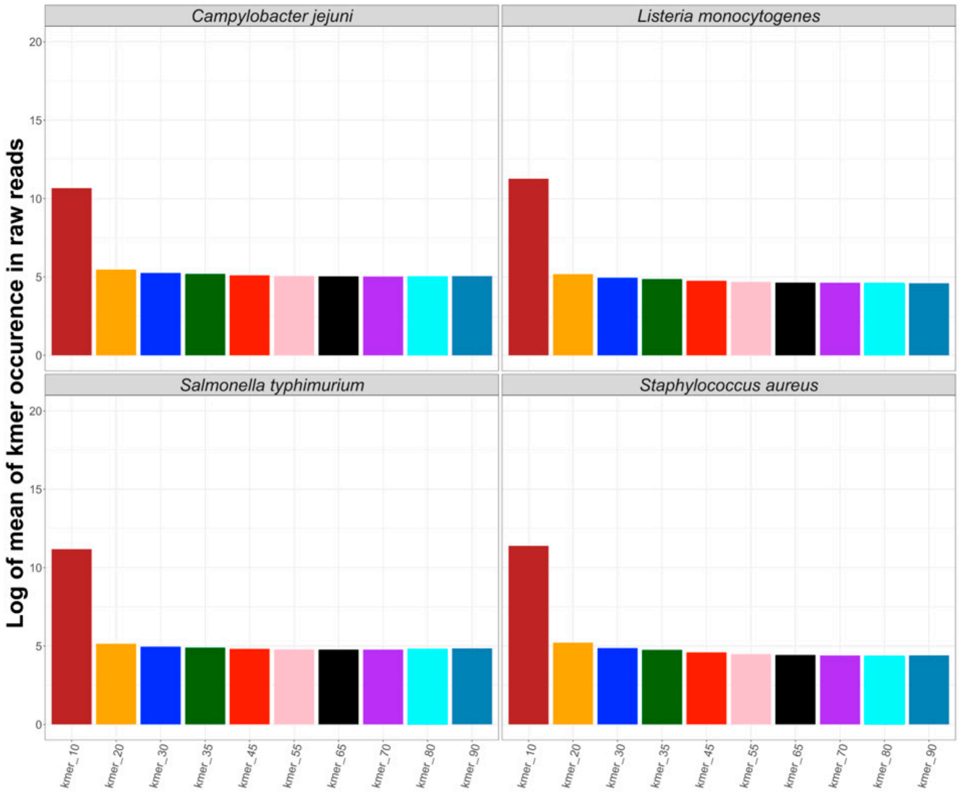

Figure S20. Distribution of k-mers across raw reads per bacterial species. Random 100 raw paired-end reads from the initial *C. jejuni*, *L. monocytogenes*, *S. aureus* and *S.* Typhimurium (major representative zoonotic serovar of *S. enterica*) datasets were selected, and DSK was used to count the occurrence of k-mers of lengths 10, 20, 30, 35, 45, 55, 65, 70, 80 and 90, respectively, in the raw reads. The mean for k-mer occurrence per organism and k-mer length was calculated, and all values were ultimately transformed to natural logarithm (base *e*) for the final visualization.
