# Supplementary material for: Systems-Based Approach for Optimization of Assembly-Free Bacterial MLST Mapping
Source: Life (Basel). 2022 Apr 30;12(5):670. doi: 10.3390/life12050670 (PMC9147691; doi:10.3390/life12050670)
Supplement: Supplementary file 1 [file life-12-00670-s001.zip › Figure S3.pdf]

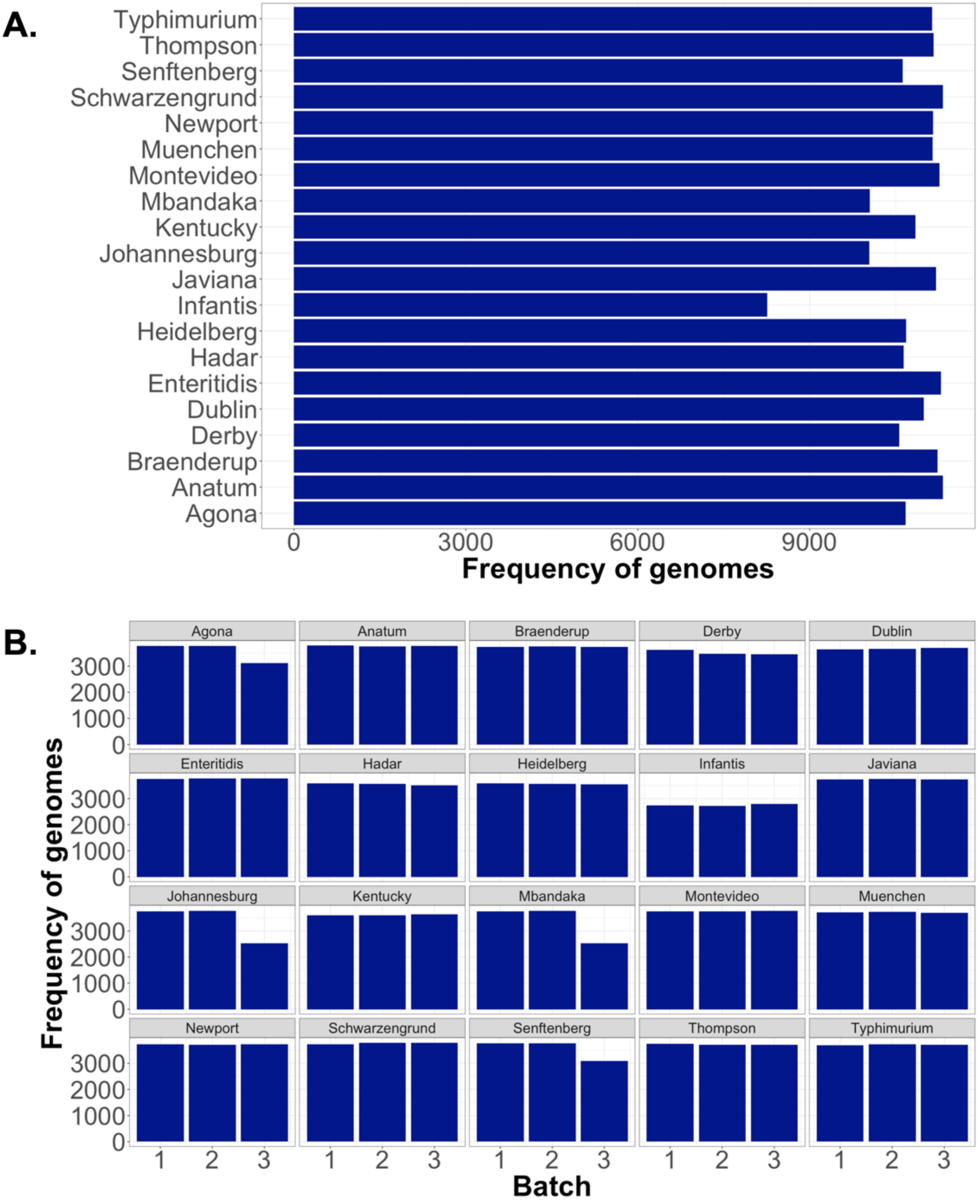
A.
Typhimurium
Thompson
Senftenberg
Schwarzengrund
Newport
Muenchen
Montevideo
Mbandaka
Kentucky
Johannesburg
Javiana
Infantis
Heidelberg
Hadar
Enteritidis
Dublin
Derby
Braenderup
Anatum
Agona
0
3000
6000
9000
Frequency of genomes
B.
Agona
Anatum
Braenderup
Derby
Dublin
Enteritidis
Hadar
Heidelberg
Infantis
Javiana
Johannesburg
Kentucky
Mbandaka
Montevideo
Muenchen
Newport
Schwarzengrund
Senftenberg
Thompson
Typhimurium
3000
2000
1000
0
Frequency of genomes
1
2
3
Batch

C.

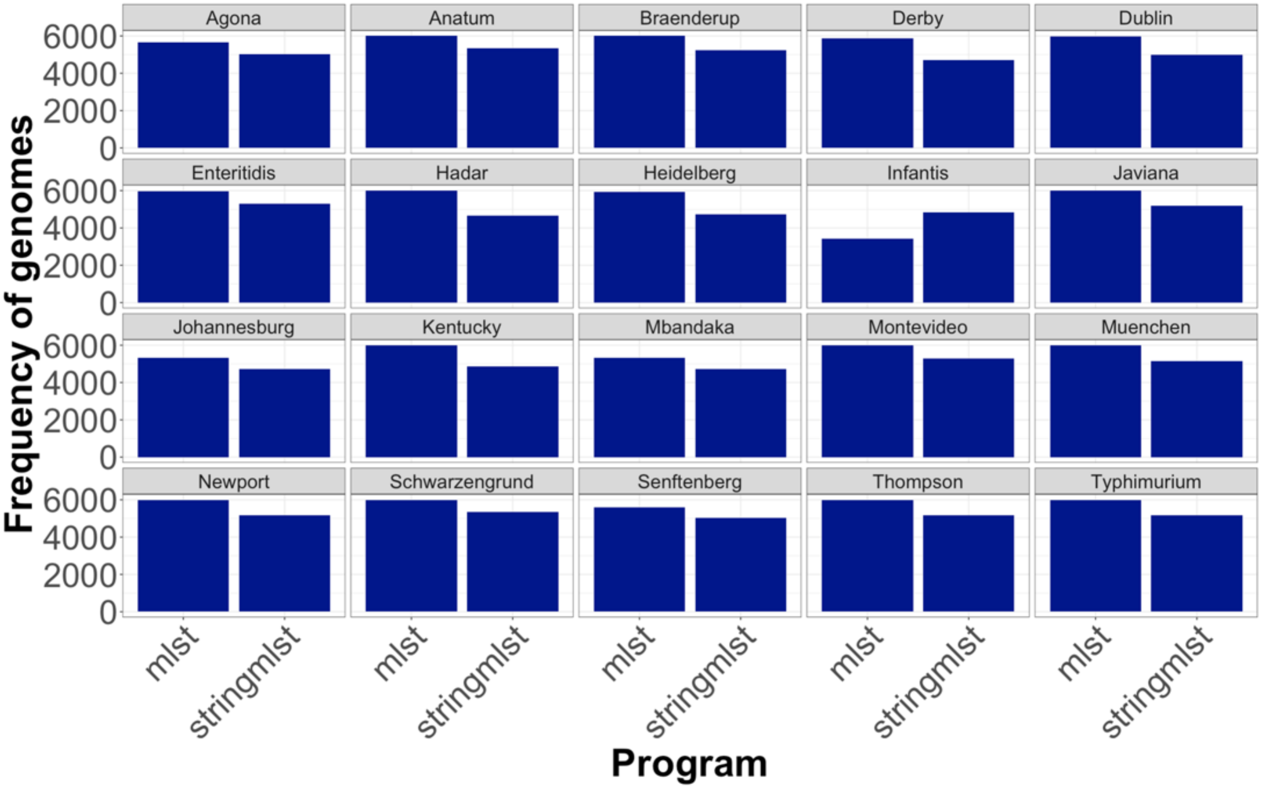

Agona
Anatum
Braenderup
Derby
Dublin
Enteritidis
Hadar
Heidelberg
Infantis
Javiana
Johannesburg
Kentucky
Mbandaka
Montevideo
Muenchen
Newport
Schwarzengrund
Senftenberg
Thompson
Typhimurium
6000
4000
2000
0
Frequency of genomes
mlst
stringmlst
Program

D.

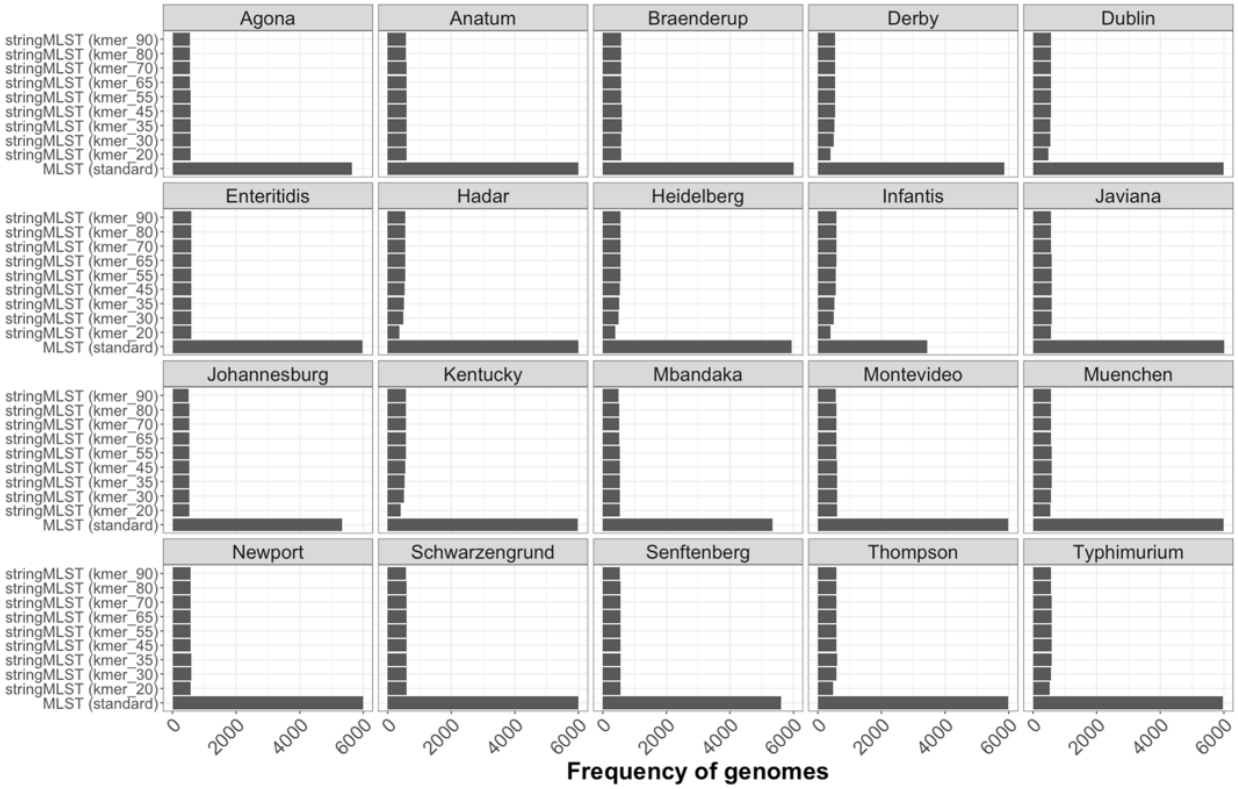

Agona
Anatum
Braenderup
Derby
Dublin
Enteritidis
Hadar
Heidelberg
Infantis
Javiana
Johannesburg
Kentucky
Mbandaka
Montevideo
Muenchen
Newport
Schwarzengrund
Senftenberg
Thompson
Typhimurium
stringMLST (kmer_90)
stringMLST (kmer_80)
stringMLST (kmer_70)
stringMLST (kmer_65)
stringMLST (kmer_55)
stringMLST (kmer_45)
stringMLST (kmer_35)
stringMLST (kmer_30)
stringMLST (kmer_20)
MLST (standard)
0
2000
4000
6000
Frequency of genomes

E.

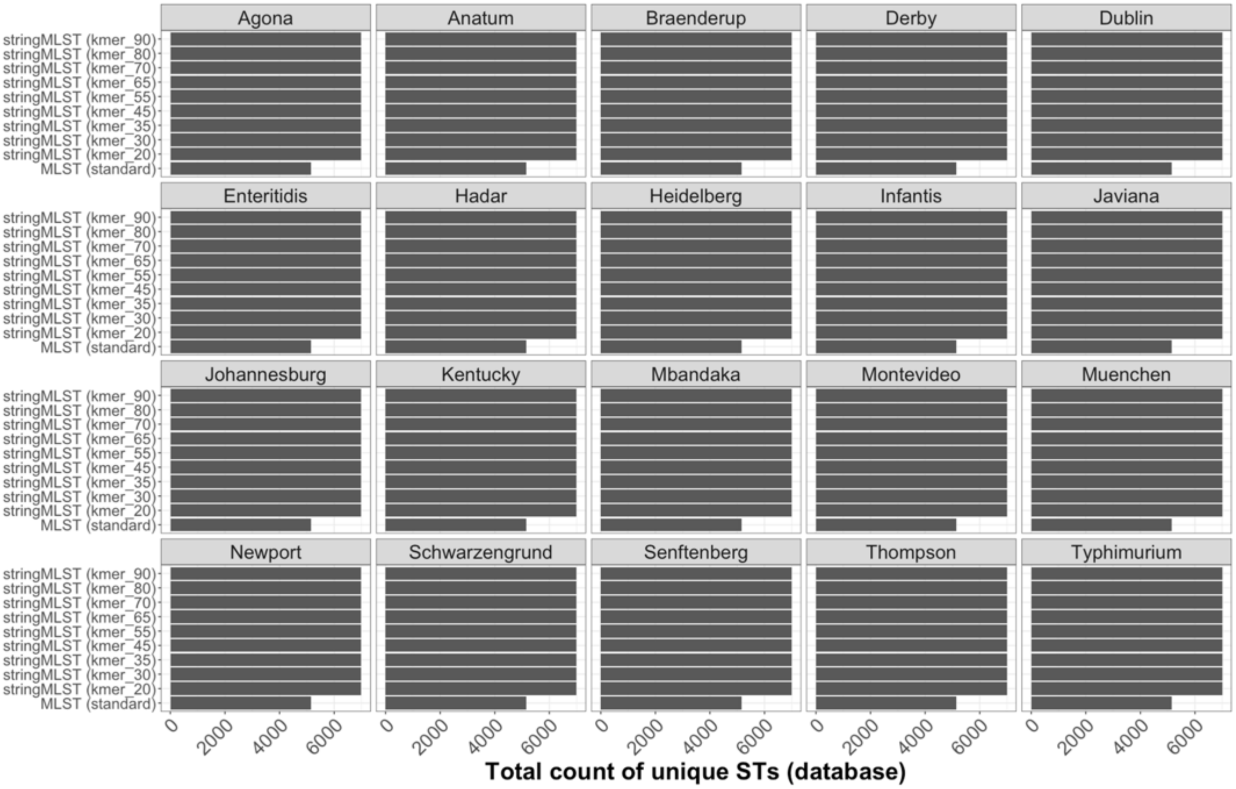
Agona
Anatum
Braenderup
Derby
Dublin
Enteritidis
Hadar
Heidelberg
Infantis
Javiana
Johannesburg
Kentucky
Mbandaka
Montevideo
Muenchen
Newport
Schwarzengrund
Senftenberg
Thompson
Typhimurium
stringMLST (kmer_90)
stringMLST (kmer_80)
stringMLST (kmer_70)
stringMLST (kmer_65)
stringMLST (kmer_55)
stringMLST (kmer_45)
stringMLST (kmer_35)
stringMLST (kmer_30)
stringMLST (kmer_20)
MLST (standard)
0
2000
4000
6000
Total count of unique STs (database)

F.

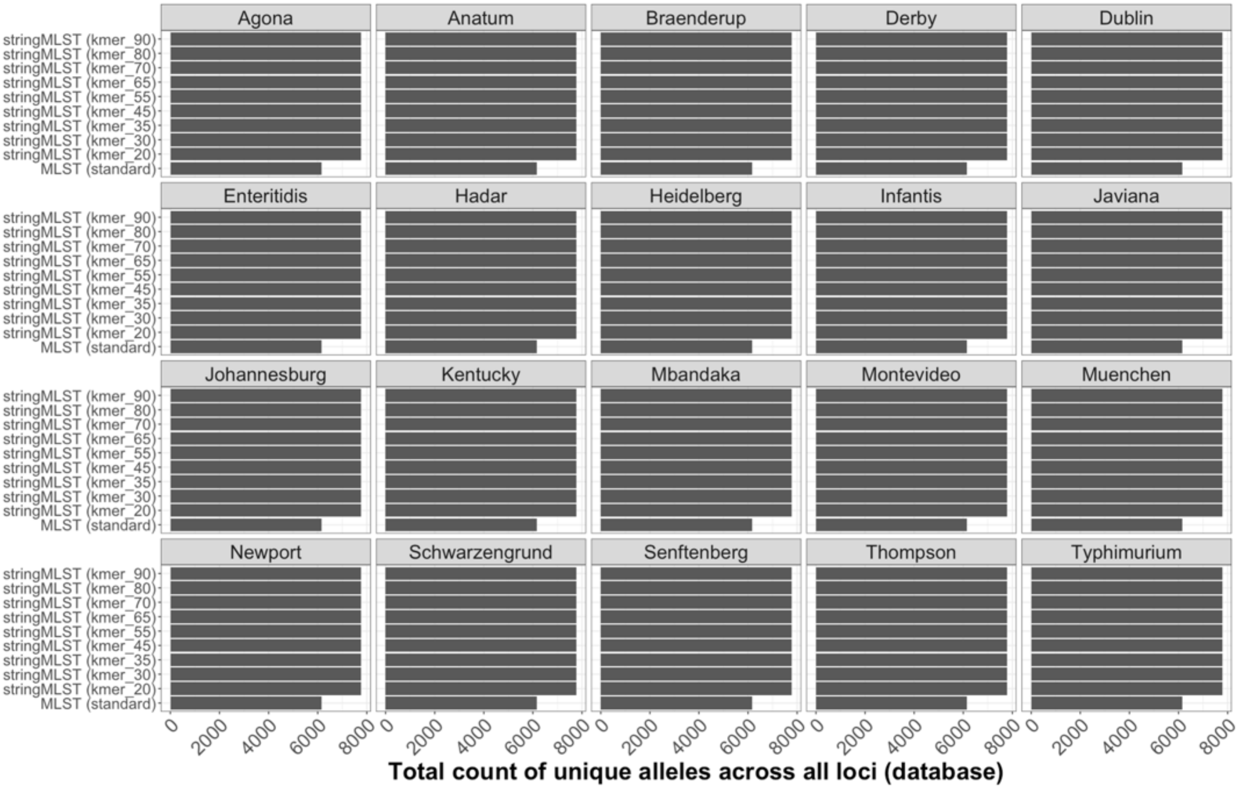
Agona
Anatum
Braenderup
Derby
Dublin
Enteritidis
Hadar
Heidelberg
Infantis
Javiana
Johannesburg
Kentucky
Mbandaka
Montevideo
Muenchen
Newport
Schwarzengrund
Senftenberg
Thompson
Typhimurium
stringMLST (kmer_90)
stringMLST (kmer_80)
stringMLST (kmer_70)
stringMLST (kmer_65)
stringMLST (kmer_55)
stringMLST (kmer_45)
stringMLST (kmer_35)
stringMLST (kmer_30)
stringMLST (kmer_20)
MLST (standard)
0
2000
4000
6000
8000
Total count of unique alleles across all loci (database)

G.

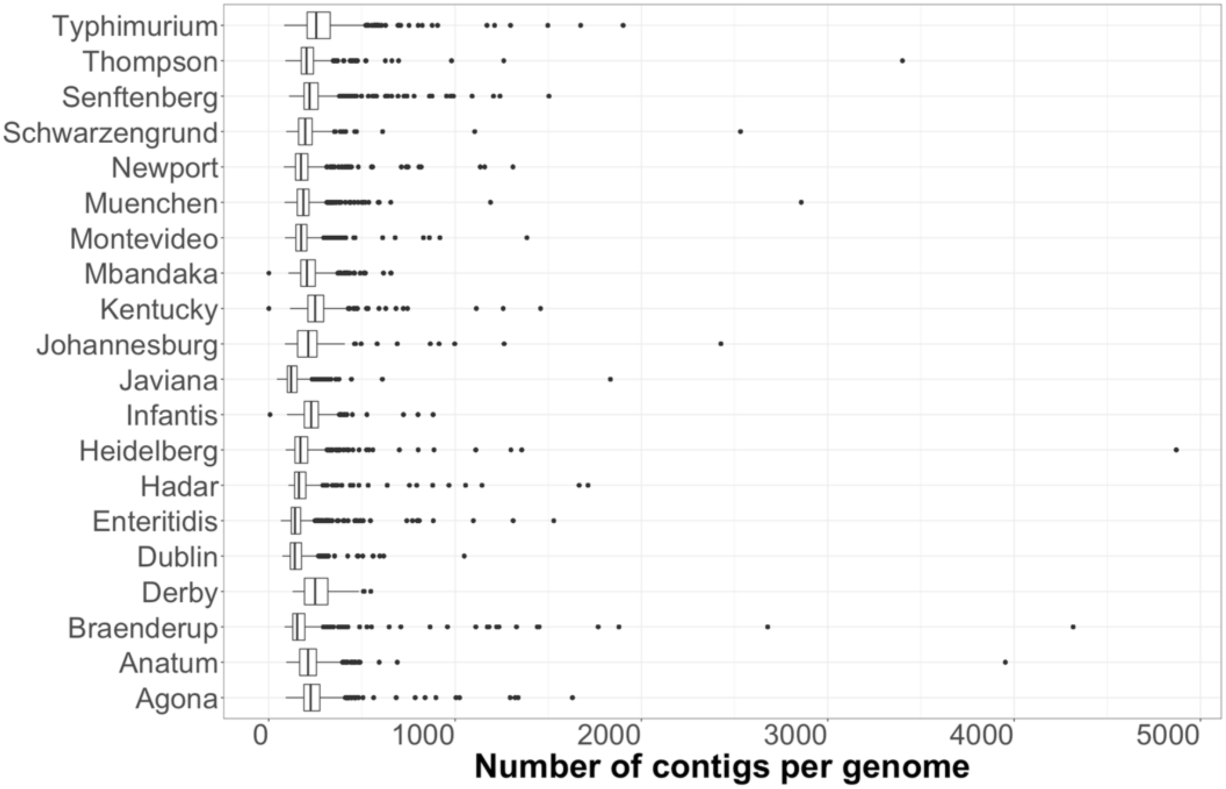
Typhimurium
Thompson
Senftenberg
Schwarzengrund
Newport
Muenchen
Montevideo
Mbandaka
Kentucky
Johannesburg
Javiana
Infantis
Heidelberg
Hadar
Enteritidis
Dublin
Derby
Braenderup
Anatum
Agona
0
1000
2000
3000
4000
5000
Number of contigs per genome

H.

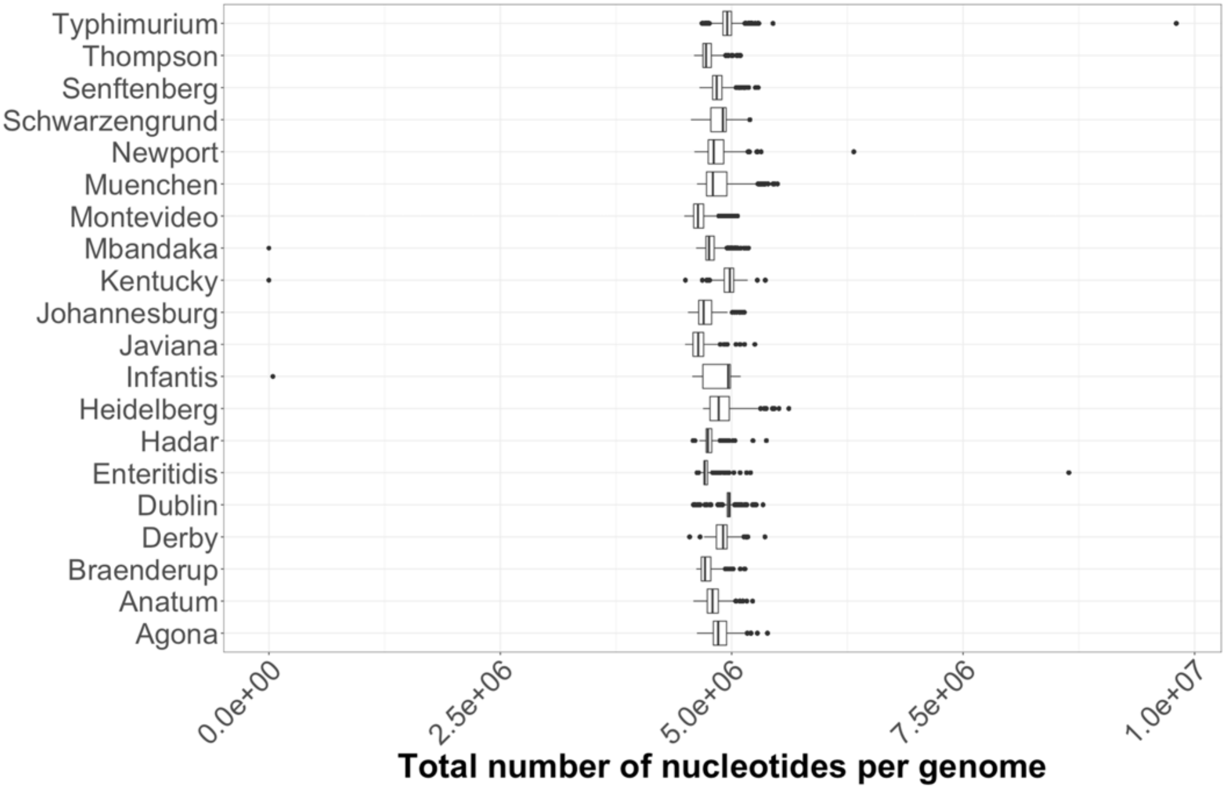
Typhimurium
Thompson
Senftenberg
Schwarzengrund
Newport
Muenchen
Montevideo
Mbandaka
Kentucky
Johannesburg
Javiana
Infantis
Heidelberg
Hadar
Enteritidis
Dublin
Derby
Braenderup
Anatum
Agona
0.0e+00
2.5e+06
5.0e+06
7.5e+06
1.0e+07
Total number of nucleotides per genome

I.

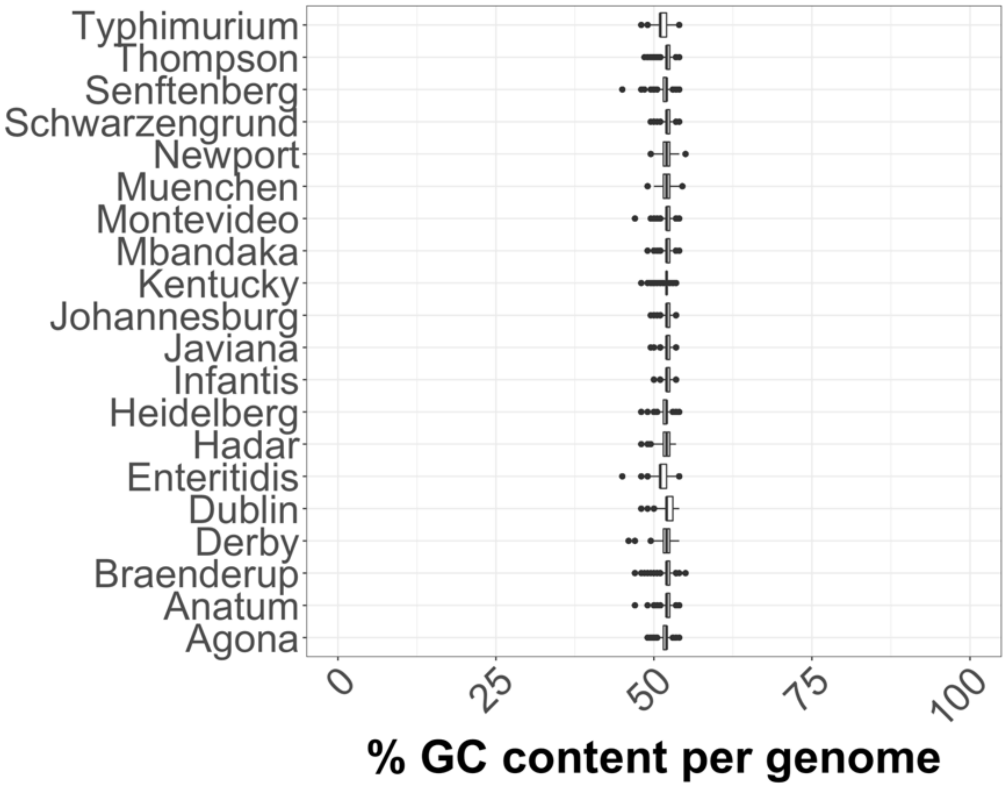

Typhimurium
Thompson
Senftenberg
Schwarzengrund
Newport
Muenchen
Montevideo
Mbandaka
Kentucky
Johannesburg
Javiana
Infantis
Heidelberg
Hadar
Enteritidis
Dublin
Derby
Braenderup
Anatum
Agona
0
25
50
75
100
% GC content per genome

J.

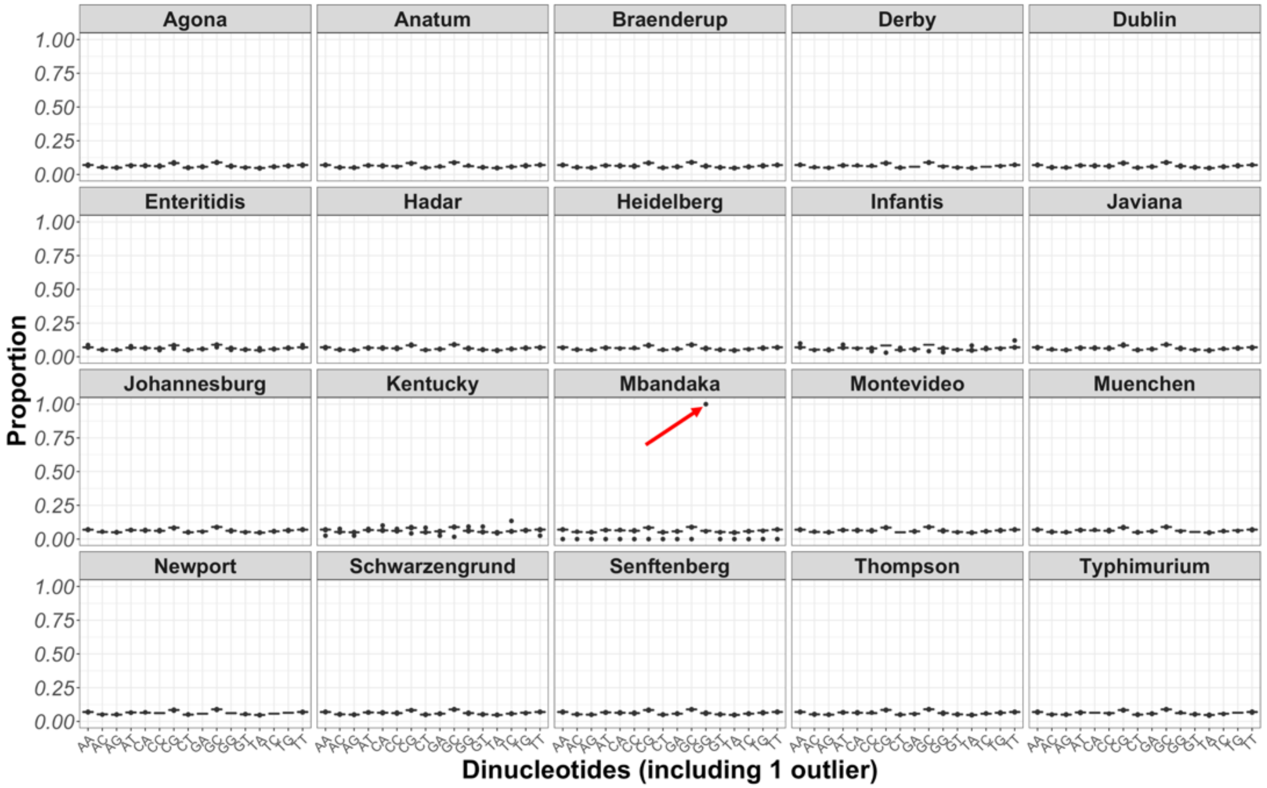

Agona
Anatum
Braenderup
Derby
Dublin
Enteritidis
Hadar
Heidelberg
Infantis
Javiana
Johannesburg
Kentucky
Mbandaka
Montevideo
Muenchen
Newport
Schwarzengrund
Senftenberg
Thompson
Typhimurium
1.00
0.75
0.50
0.25
0.00
Proportion
Dinucleotides (including 1 outlier)

K.

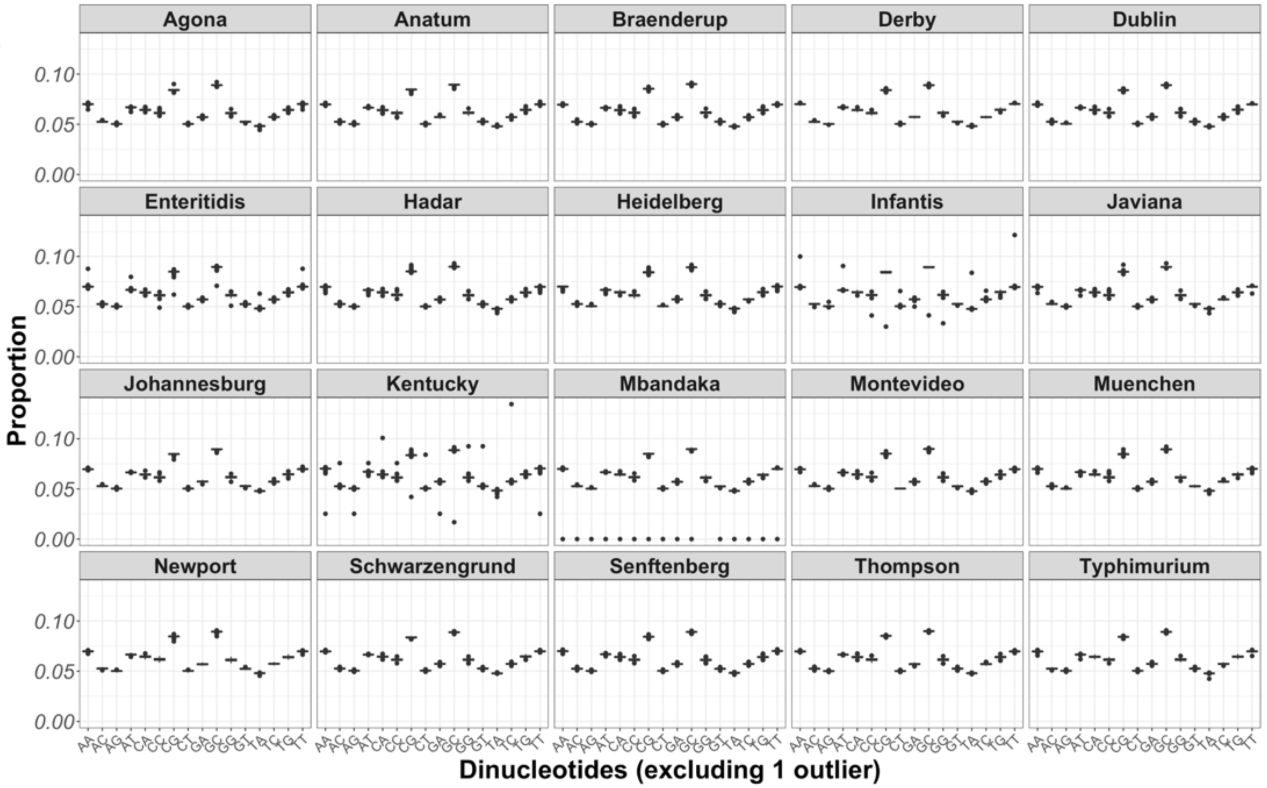
Agona
Anatum
Braenderup
Derby
Dublin
Enteritidis
Hadar
Heidelberg
Infantis
Javiana
Johannesburg
Kentucky
Mbandaka
Montevideo
Muenchen
Newport
Schwarzengrund
Senftenberg
Thompson
Typhimurium
Proportion
0.00
0.05
0.10
Dinucleotides (excluding 1 outlier)

L.

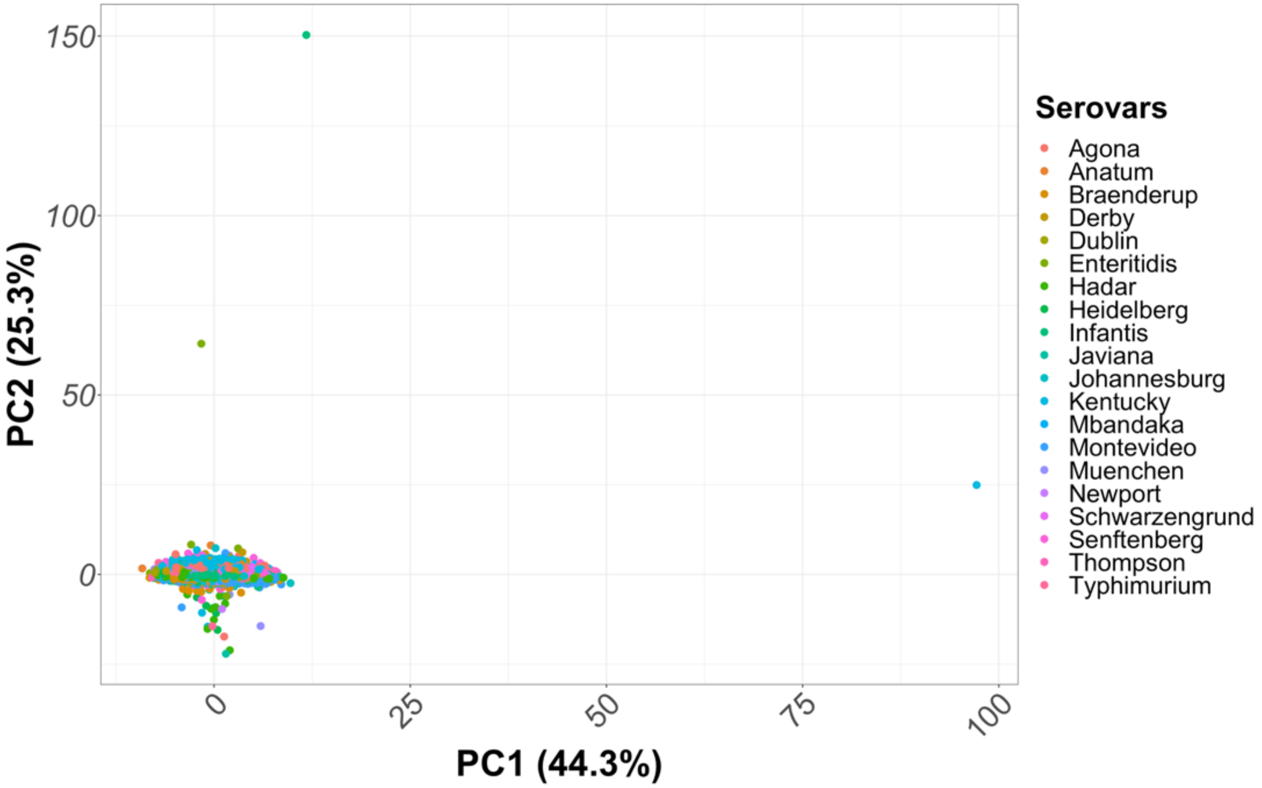
PC2 (25.3%)
150
100
50
0
0
25
50
75
100
PC1 (44.3%)
Serovars
Agona
Anatum
Braenderup
Derby
Dublin
Enteritidis
Hadar
Heidelberg
Infantis
Javiana
Johannesburg
Kentucky
Mbandaka
Montevideo
Muenchen
Newport
Schwarzengrund
Senftenberg
Thompson
Typhimurium

M.

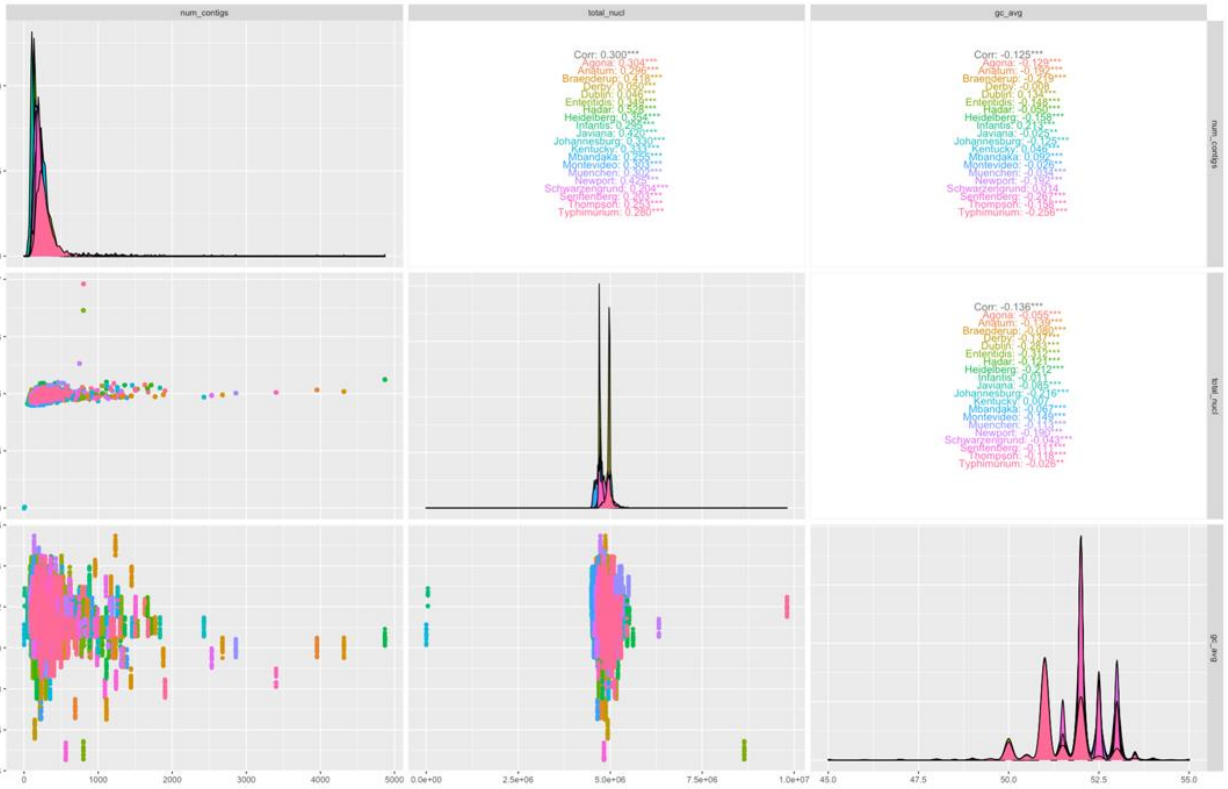
num_contigs
total_nucl
gc_avg
Corr: 0.300***
Corr: -0.125***
Corr: -0.136***

N.

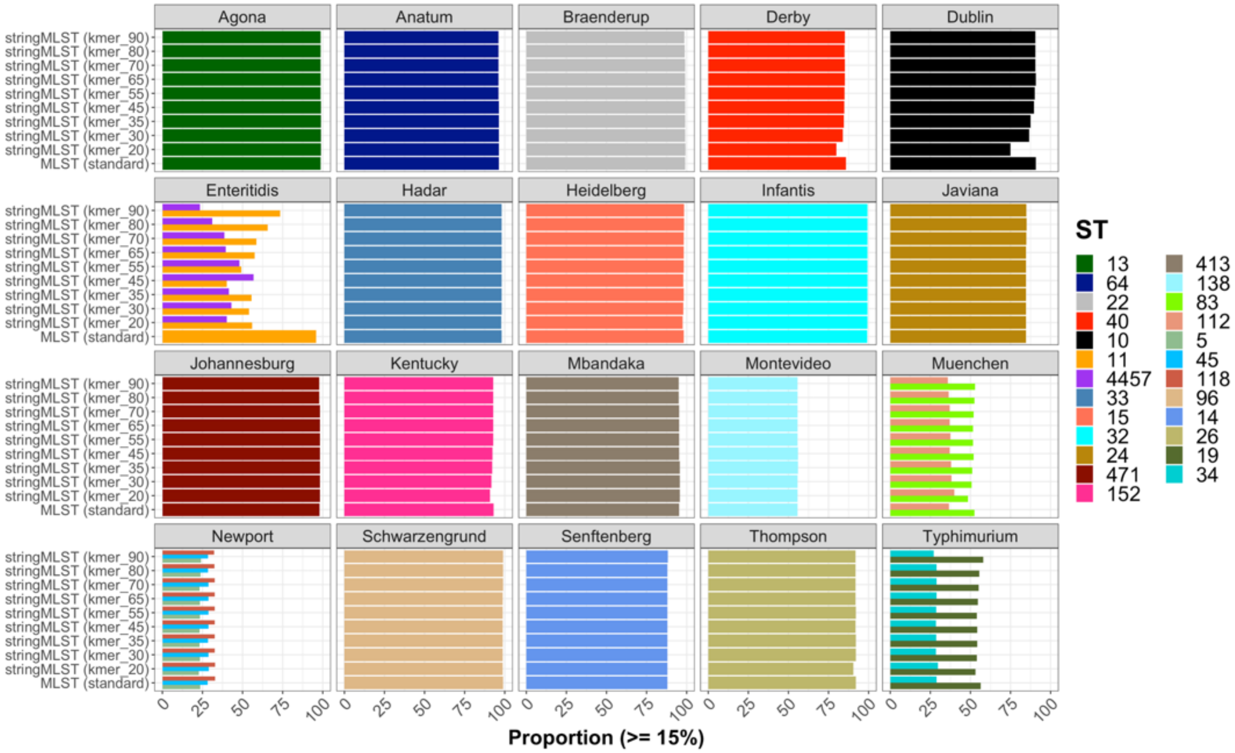
Agona
Anatum
Braenderup
Derby
Dublin
Enteritidis
Hadar
Heidelberg
Infantis
Javiana
Johannesburg
Kentucky
Mbandaka
Montevideo
Muenchen
Newport
Schwarzengrund
Senftenberg
Thompson
Typhimurium
stringMLST (kmer_90)
stringMLST (kmer_80)
stringMLST (kmer_70)
stringMLST (kmer_65)
stringMLST (kmer_55)
stringMLST (kmer_45)
stringMLST (kmer_35)
stringMLST (kmer_30)
stringMLST (kmer_20)
MLST (standard)
ST
13
64
22
40
10
11
4457
33
15
32
24
471
152
413
138
83
112
5
45
118
96
14
26
19
34
Proportion (>= 15%)

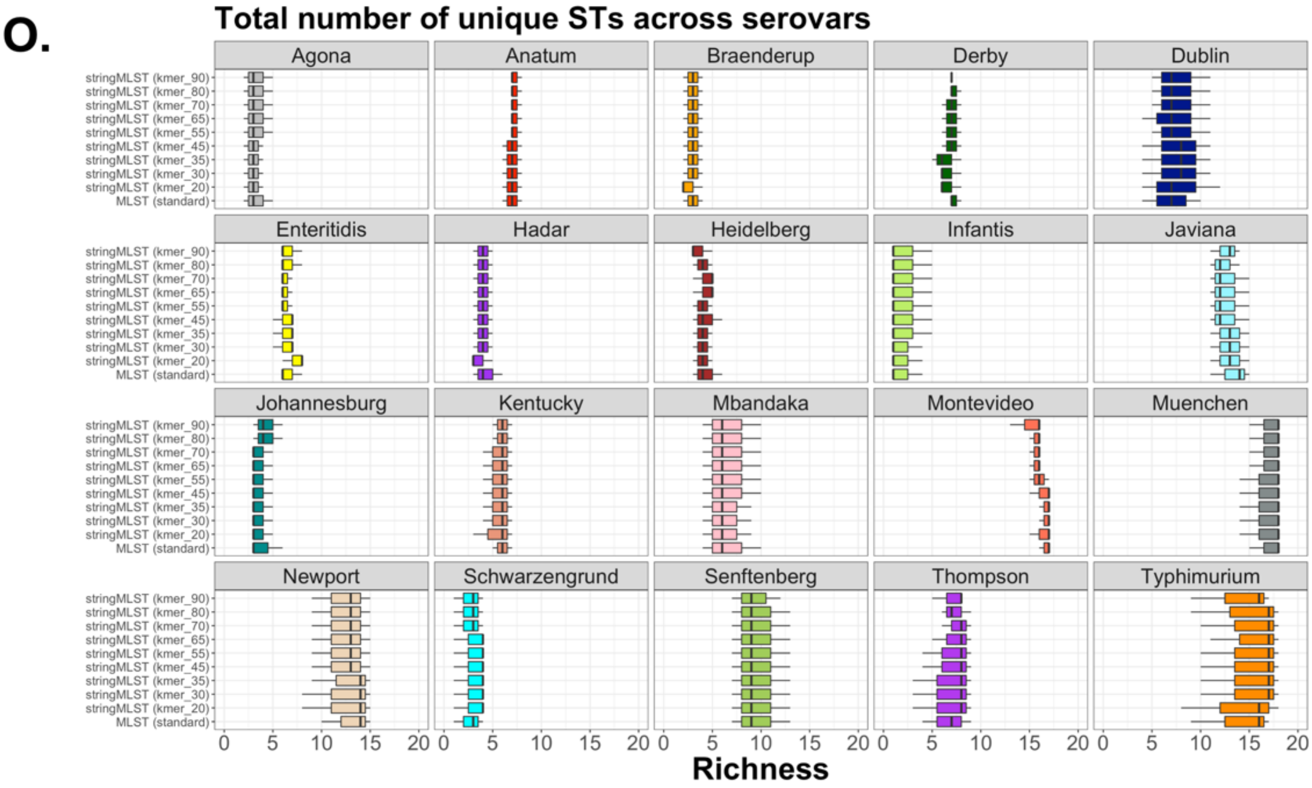
O.
Total number of unique STs across serovars
Agona
Anatum
Braenderup
Derby
Dublin
Enteritidis
Hadar
Heidelberg
Infantis
Javiana
Johannesburg
Kentucky
Mbandaka
Montevideo
Muenchen
Newport
Schwarzengrund
Senftenberg
Thompson
Typhimurium
stringMLST (kmer_90)
stringMLST (kmer_80)
stringMLST (kmer_70)
stringMLST (kmer_65)
stringMLST (kmer_55)
stringMLST (kmer_45)
stringMLST (kmer_35)
stringMLST (kmer_30)
stringMLST (kmer_20)
MLST (standard)
0 5 10 15 20
Richness

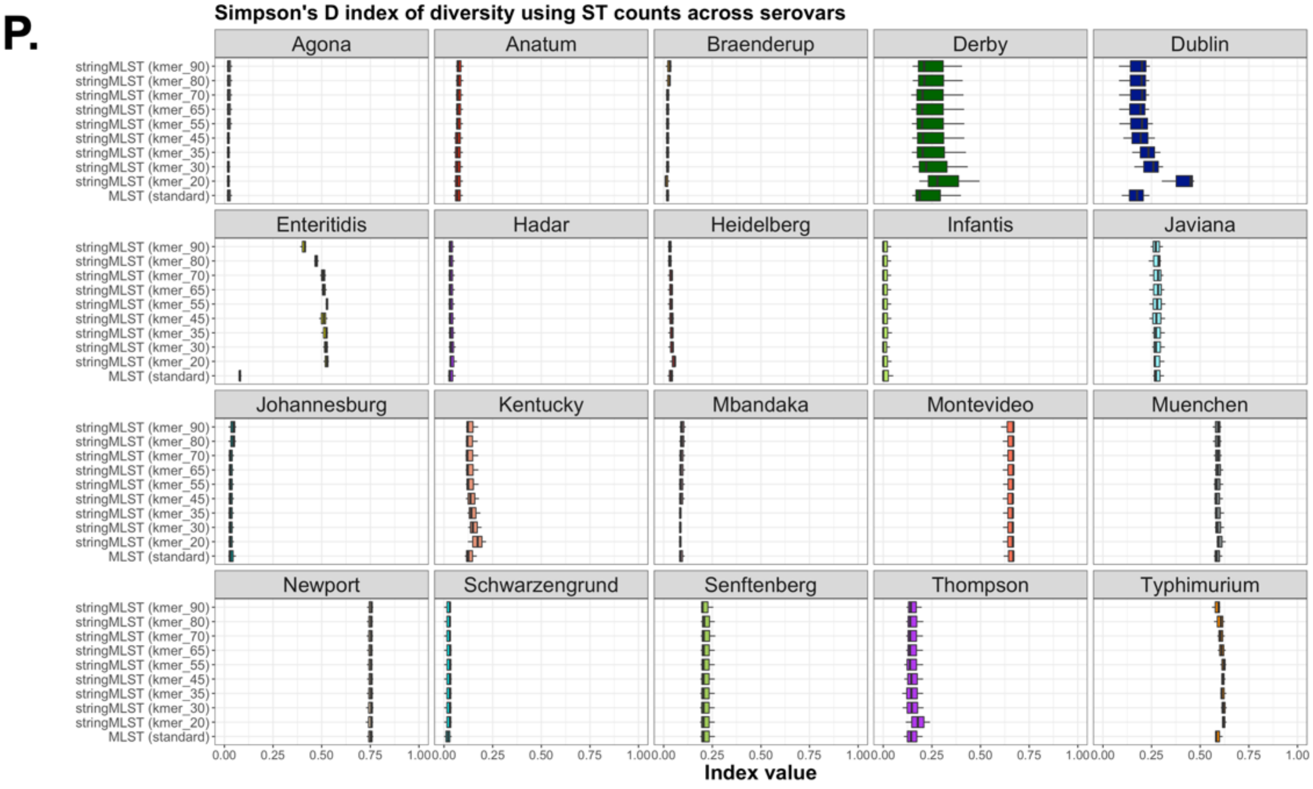
P.
Simpson's D index of diversity using ST counts across serovars
Agona
Anatum
Braenderup
Derby
Dublin
Enteritidis
Hadar
Heidelberg
Infantis
Javiana
Johannesburg
Kentucky
Mbandaka
Montevideo
Muenchen
Newport
Schwarzengrund
Senftenberg
Thompson
Typhimurium
stringMLST (kmer_90)
stringMLST (kmer_80)
stringMLST (kmer_70)
stringMLST (kmer_65)
stringMLST (kmer_55)
stringMLST (kmer_45)
stringMLST (kmer_35)
stringMLST (kmer_30)
stringMLST (kmer_20)
MLST (standard)
0.00 0.25 0.50 0.75 1.00
Index value

Q.

C. Proportion of non-classified STs across serovars

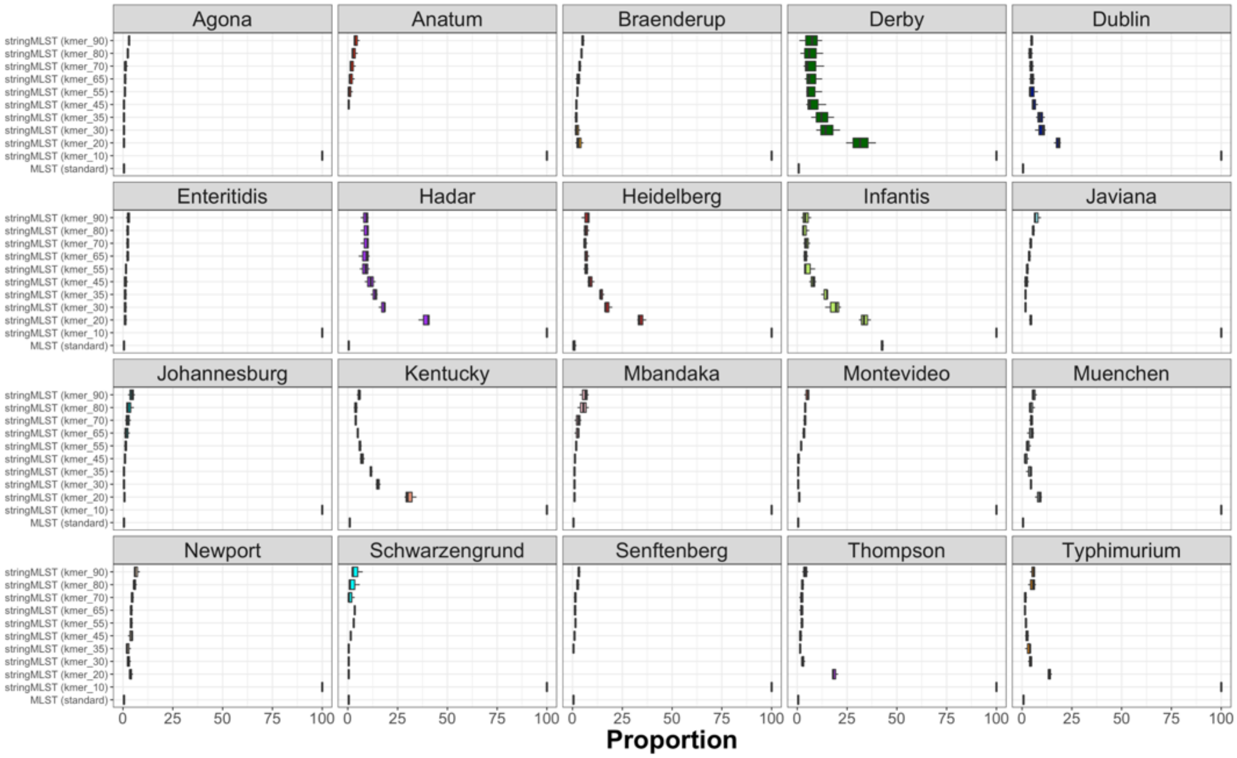

R.

D. Standard deviation for the proportion of non-classified STs across serovars

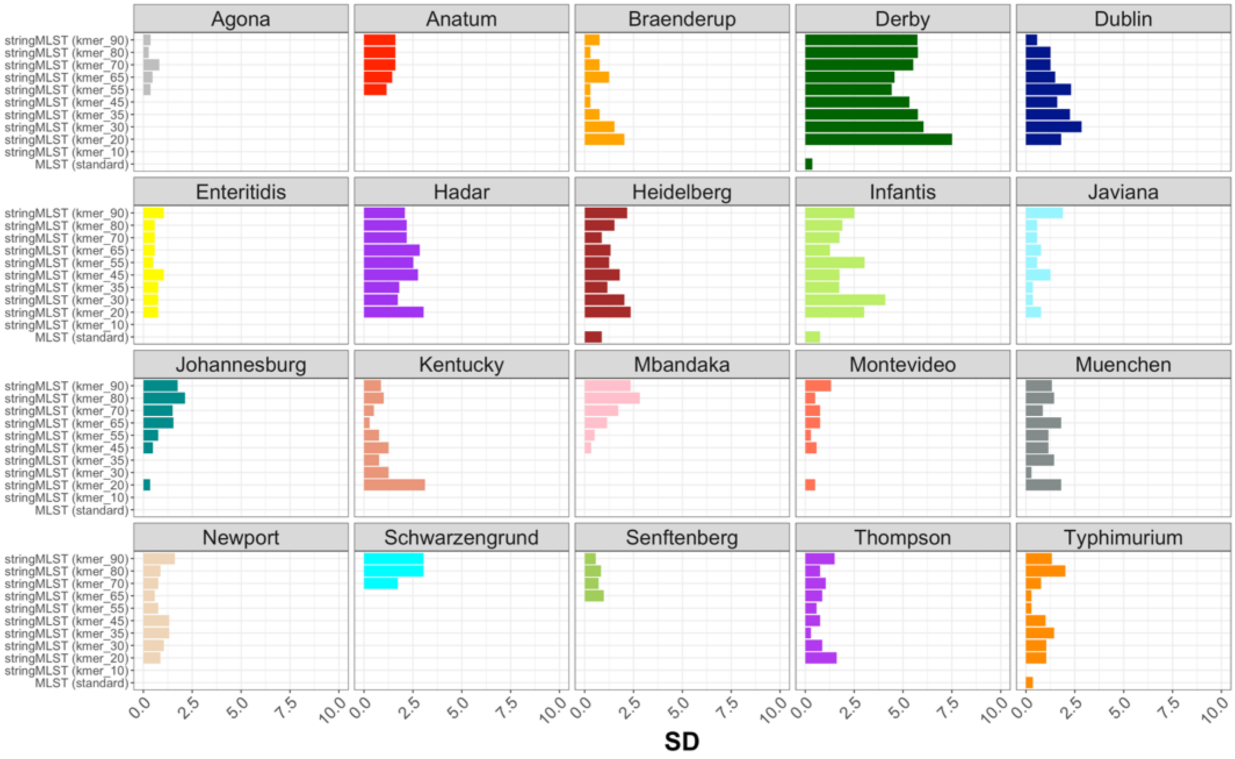

Figure S3. Summary statistics of the frequency of genomes, including the distribution of dinucleotides and bivariate associations between genome-intrinsic variables across all twenty *S. enterica* serovars (serovars).
Frequency-based distribution of randomly selected genomes across serovars (A), including a stratification by batch (B), program (C), and further discrimination by k-mer length used by stringMLST (D). Total count of unique STs (E), and alleles across all loci (F) by serovar and program. (G) Total number of contigs per genome across all serovars. (H) Total number of nucleotides per genome across all serovars. (I) Percent of GC% content per genome across all serovars. Proportion of all sixteen pairs of dinucleotides present, across serovars, with (J) or without outliers (red-circled data points) (K). (L) Principal component analysis plot depicting two PCs, along with variance explained, for the distribution of serovars using the dinucleotide data as input. (M) Bivariate association between genome-intrinsic variables across species with statistical significance measured by the Pearson correlation coefficient (Corr). Genome-intrinsic variables used were the total number of contigs (num_contigs), the total number of nucleotides per genome (assembly), and the GC% content per genome (gc_avg). (M) Asterisks refer to the degree of significance for the correlation coefficient, with *p*-value thresholds being: $*p < 0.05$, $**p \leq 0.01$, $***p \leq 0.001$, $****p \leq 0.0001$ and NS = not significant at $p \geq 0.05$. (N) Proportion of dominant STs (proportion ≥ 15%) across programs and serovars. Distribution of ST richness (O), Simpson's D index of diversity ($1 - D$) (P), proportion of non-classified STs (Q), and the standard deviation of the proportion of non-classified STs (R) across programs and serovars.
