# Supplementary material for: Systems-Based Approach for Optimization of Assembly-Free Bacterial MLST Mapping
Source: Life (Basel). 2022 Apr 30;12(5):670. doi: 10.3390/life12050670 (PMC9147691; doi:10.3390/life12050670)
Supplement: Supplementary file 1 [file life-12-00670-s001.zip › Figure S4.pdf]

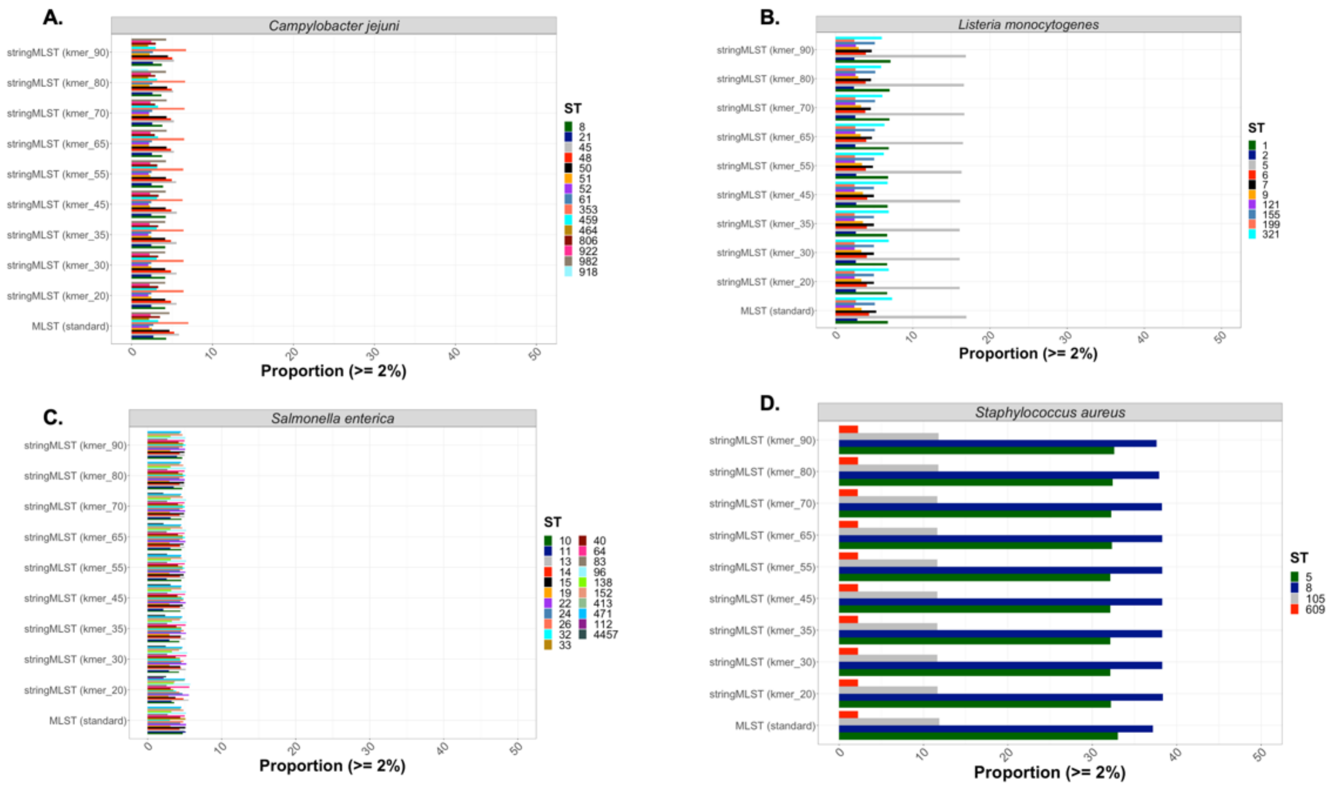

Figure S4. Relative frequency of dominant STs across programs and bacterial species.

Plots A-D depict the proportion of STs across all four bacterial species including *C. jejuni*, *L. monocytogenes*, *S. enterica* (all twenty serovars), and *S. aureus*, respectively. Only STs with a proportion equal or above 2% were shown across all plots.
