# Supplementary material for: Systems-Based Approach for Optimization of Assembly-Free Bacterial MLST Mapping
Source: Life (Basel). 2022 Apr 30;12(5):670. doi: 10.3390/life12050670 (PMC9147691; doi:10.3390/life12050670)
Supplement: Supplementary file 1 [file life-12-00670-s001.zip › Figure S5.pdf]

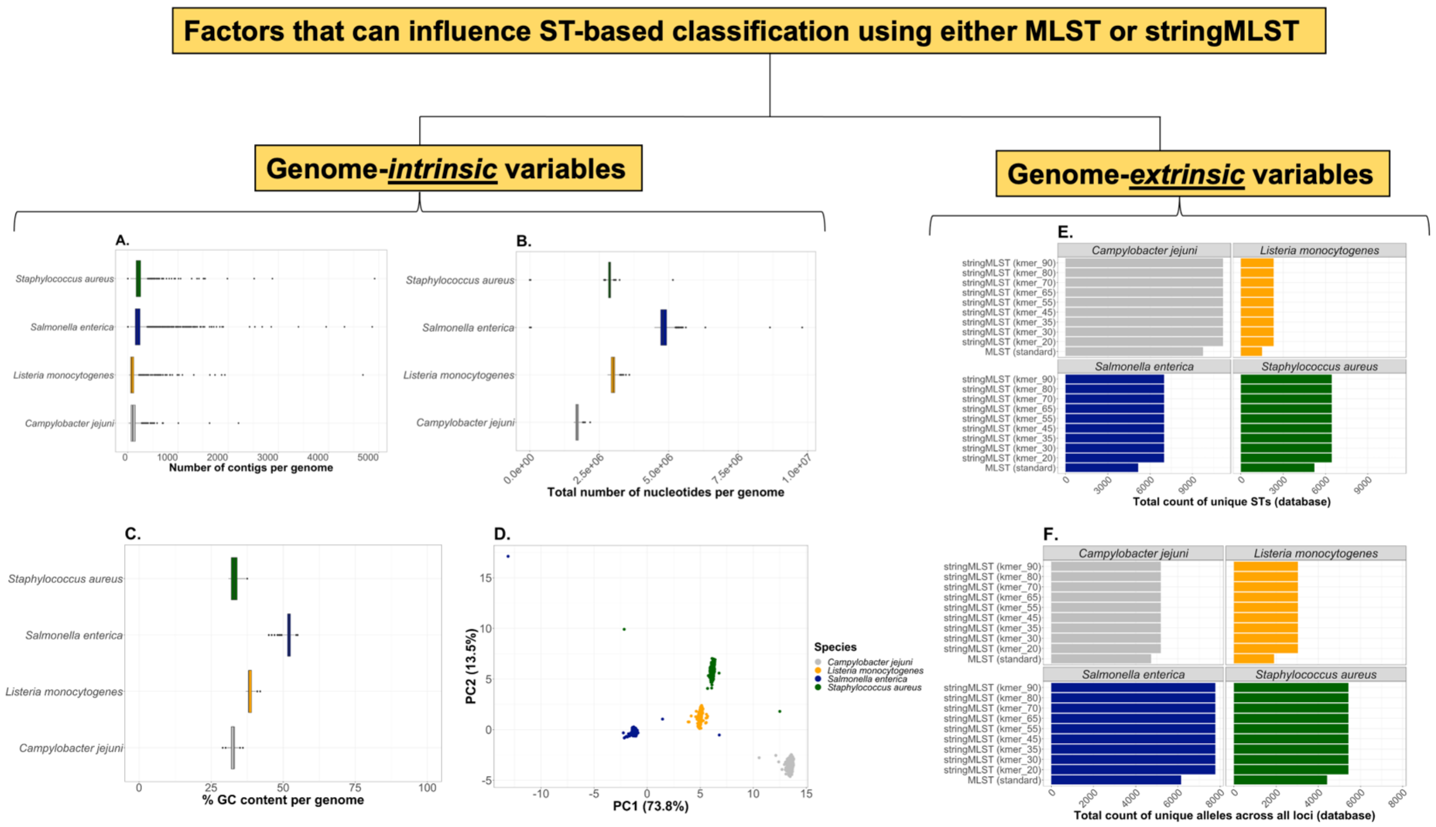

Figure S5. Genome-intrinsic and –extrinsic variables that can impact the accuracy of ST-based classification using either mlst (MLST-based genotyping) or stringMLST algorithmic approaches.

Box-and-whiskers plot showing genome-intrinsic variables, varying in distribution according to the bacterial species (A-C as y-axis), that may affect ST-based classification, include: (A) Number of contigs per genome (x-axis); (B) Total number of nucleotides per genome (x-axis); (C) GC% content per genome (x-axis); and (D) Dinucleotide composition of genomes. (D) Inter-species PCA using the relative frequency of all pairs of dinucleotides (16 pairs) present in the genome as input data. Only two PCs are shown, and the percentage of variance explained by either PC is depicted in parenthesis. Bar plots showing genome-extrinsic variables that may influence the performance of mlst vs. stringMLST across species include but are not limited to: (E) Total count of unique STs per database (ST richness in the database used for mapping of raw reads or assemblies) (x-axis); and (F) Total count of unique alleles across all seven loci used for ST classification (x-axis). Specifically, the differences in ST richness and allelic composition in the databases reflect difference between mlst vs. stringMLST, and were not impacted by the k-mer length (E-F, y-axis).
