# Supplementary material for: Systems-Based Approach for Optimization of Assembly-Free Bacterial MLST Mapping
Source: Life (Basel). 2022 Apr 30;12(5):670. doi: 10.3390/life12050670 (PMC9147691; doi:10.3390/life12050670)
Supplement: Supplementary file 1 [file life-12-00670-s001.zip › Figure S6.pdf]

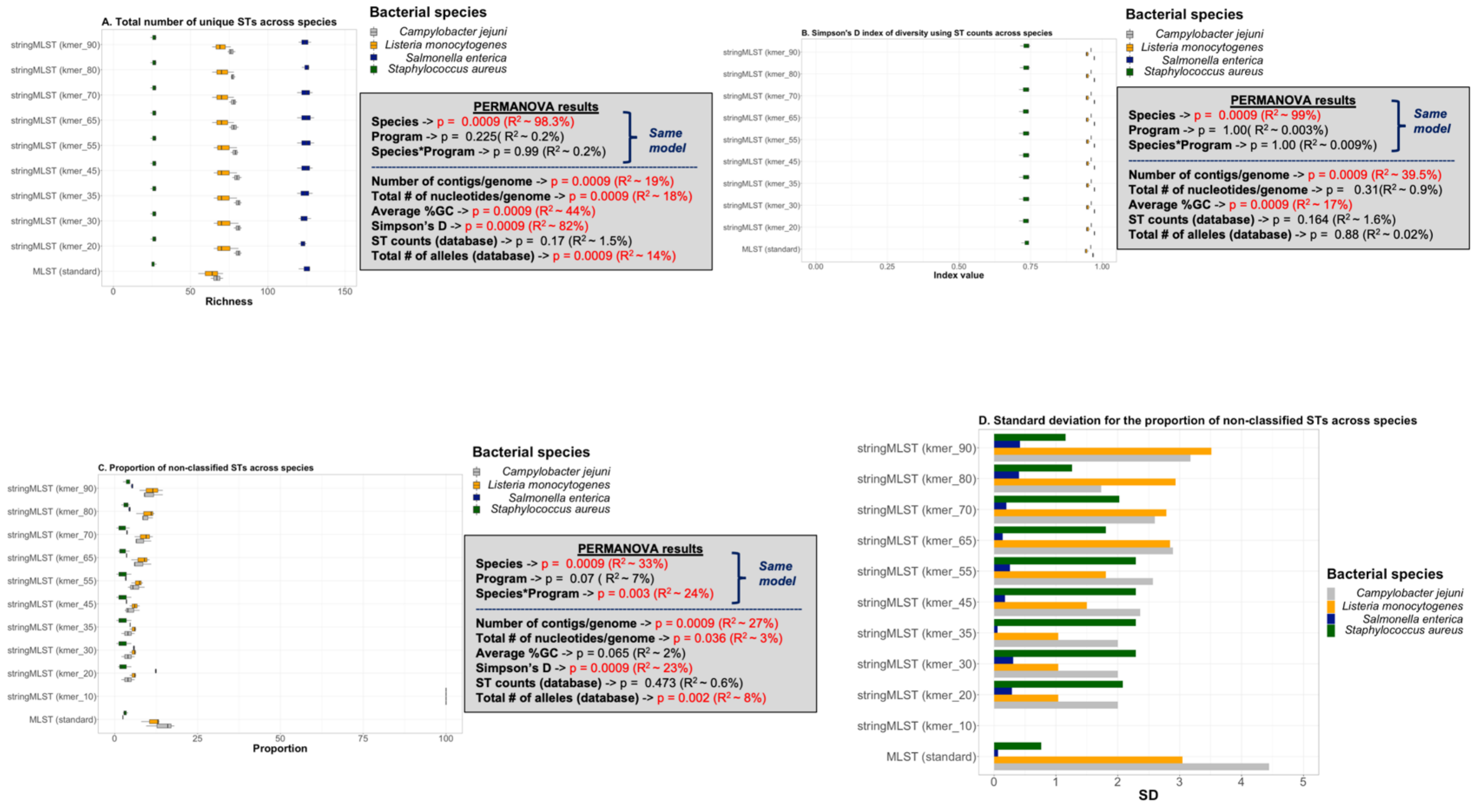

Figure S6. Statistical analysis of ST-based classification outcomes for comparison between mlst and stringMLST performance across bacterial species.
(A-C) Box-and-whiskers plots A-C demonstrate the relationship between ST richness (x-axis), Simpson's index of diversity (1 – *D*) based on ST composition (x-axis), or the proportion of non-classified STs (x-axis) across bacterial species (color-coded differently) and programs (y-axis), respectively. Along with plots A-C are depicted all PERMANOVA results including *p*-values ($p < 0.05$) and the univariate or synergistic contribution of factors measured by *R*-squared. PERMANOVA modeling was done in two specific ways: 1) A model including species, program, and their interaction, considering that those were the main variables of interest; and 2) All other results were calculated using univariate models and included modeling using genome-intrinsic (number of contigs per genome, total number of nucleotides per genome, and average GC% content) and –extrinsic (Simpson's D index of diversity, ST and allelic counts per database) variables. (D) Bar plot depicting the distribution of the standard deviation (SD, y-axis) for the proportion of non-classified STs based on species (color-coded differently) and programs (y-axis).
