# Supplementary material for: Systems-Based Approach for Optimization of Assembly-Free Bacterial MLST Mapping
Source: Life (Basel). 2022 Apr 30;12(5):670. doi: 10.3390/life12050670 (PMC9147691; doi:10.3390/life12050670)
Supplement: Supplementary file 1 [file life-12-00670-s001.zip › Figure S7.pdf]

A.

PERMANOVA model using ST richness as *dependent variable* and the following independent variables: bacterial species (species) and program as MLST or stringMLST (program)

Model = adonis(formula = n ~ species * program, data = d14, permutations = 1000)

	Df	SumsOfSqs	MeanSqs	F.Model	R^2	Pr(>F)
**species**	3	7.51793789980392	2.50597929993464	2011.1886976523	0.982921621030805	0.000999000999000999
**program**	9	0.0146871548379872	0.00163190609310969	1.30969600993022	0.00192025023804218	0.224775224775225
**species:program**	27	0.0162563830939995	0.000602088262740721	0.483209541692874	0.00212541665491391	0.99000999000999
**Residuals**	80	0.099681518809644	0.00124601898512055		0.0130327120762388	
**Total**	119	7.64856295654555			1	

B.

PERMANOVA model using ST richness as *dependent variable* and the following independent variable: bacterial species (species)

Model = adonis(formula = n ~ species, data = d14, permutations = 1000)

	Df	SumsOfSqs	MeanSqs	F.Model	R^2	Pr(>F)
**species**	3	7.51793789980392	2.50597929993464	2225.40457431069	0.982921621030805	0.000999000999000999
**Residuals**	116	0.130625056741631	0.00112607807535889		0.0170783789691949	
**Total**	119	7.64856295654555			1	

C.

PERMANOVA model using ST richness as *dependent variable* and the following independent variable:
program as MLST or stringMLST (program)

Model = adonis(formula = n ~ program, data = d14, permutations = 1000)

	Df	SumsOfSqs	MeanSqs	F.Model	R^2	Pr(>F)
**program**	9	0.0146871548379877	0.00163190609310974	0.0235148795847475	0.00192025023804224	1
**Residuals**	110	7.63387580170756	0.0693988709246142		0.998079749761958	
**Total**	119	7.64856295654555			1	

D.

PERMANOVA model using ST richness as *dependent variable* and the following independent variable:
median of the number of contigs (num_contigs_median)

Model = adonis(formula = n ~ num_contigs_median, data = d14, permutations = 1000)

	Df	SumsOfSqs	MeanSqs	F.Model	R^2	Pr(>F)
**num_contigs_median**	1	1.44641113449941	1.44641113449941	27.5189190410083	0.189108874793478	0.000999000999000999
**Residuals**	118	6.20215182204614	0.052560608661408		0.810891125206522	
**Total**	119	7.64856295654555			1	

E.

PERMANOVA model using ST richness as *dependent variable* and the following independent variable: mean of the total counts for nucleotides per genomes (total_nucl_mean)

Model = adonis(formula = n ~ total_nucl_mean, data = d14, permutations = 1000)

	Df	SumsOfSqs	MeanSqs	F.Model	R^2	Pr(>F)
**total_nucl_mean**	1	1.3737610065047	1.3737610065047	25.834090072994	0.179610341747798	0.000999000999000999
**Residuals**	118	6.27480195004085	0.0531762877122106		0.820389658252202	
**Total**	119	7.64856295654555			1	

F.

PERMANOVA model using ST richness as *dependent variable* and the following independent variable: mean of the average GC% per genome (gc_avg_mean)

Model = adonis(formula = n ~ gc_avg_mean, data = d14, permutations = 1000)

	Df	SumsOfSqs	MeanSqs	F.Model	R^2	Pr(>F)
**gc_avg_mean**	1	3.35589095505983	3.35589095505983	92.2491009236216	0.438760976947689	0.000999000999000999
**Residuals**	118	4.29267200148572	0.0363785762837772		0.56123902305231	
**Total**	119	7.64856295654555			1	

G.

PERMANOVA model using ST richness as *dependent variable* and the following independent variable: mean of the total counts of unique STs per program (st_count_mean)

Model = adonis(formula = n ~ st_count_mean, data = d14, permutations = 1000)

	Df	SumsOfSqs	MeanSqs	F.Model	R^2	Pr(>F)
**st_count_mean**	1	0.118289690970522	0.118289690970522	1.85360916426925	0.0154656098985615	0.166833166833167
**Residuals**	118	7.53027326557503	0.0638158751319917		0.984534390101439	
**Total**	119	7.64856295654555			1	

H.

PERMANOVA model using ST richness as *dependent variable* and the following independent variable: mean of the total counts of unique alleles across all genes per program (total_alleles_genes_mean)

Model = adonis(formula = n ~ total_alleles_genes_mean, data = d14, permutations = 1000)

	Df	SumsOfSqs	MeanSqs	F.Model	R^2	Pr(>F)
**total_alleles_genes_mean**	1	1.06185600851364	1.06185600851364	19.0230125604796	0.138830786194277	0.000999000999000999
**Residuals**	118	6.58670694803191	0.0558195504070501		0.861169213805723	
**Total**	119	7.64856295654555			1	

I.

PERMANOVA model using ST richness as *dependent variable* and the following independent variable:
Simpson's D index of diversity per species (simpson)

Model = adonis(formula = n ~ simpson, data = d14, permutations = 1000)

	Df	SumsOfSqs	MeanSqs	F.Model	R^2	Pr(>F)
**simpson**	1	6.2561804696614	6.2561804696614	530.191454125543	0.817955020466614	0.000999000999000999
**Residuals**	118	1.39238248688415	0.011799851583764		0.182044979533386	
**Total**	119	7.64856295654555			1	

J.

PERMANOVA model using ST richness as *dependent variable* and the following independent variable:
Standard deviation of the number of contigs (num_contigs_sd)

Model = adonis(formula = n ~ num_contigs_sd, data = d16, permutations = 1000)

	Df	SumsOfSqs	MeanSqs	F.Model	R^2	Pr(>F)
**num_contigs_sd**	1	3.8179928916089	3.8179928916089	117.612562509622	0.499177808079818	0.000999000999000999
**Residuals**	118	3.83057006493665	0.0324624581774292		0.500822191920182	
**Total**	119	7.64856295654555			1	

K.

PERMANOVA model using ST richness as *dependent variable* and the following independent variable: Standard deviation of the total counts for nucleotides per genomes (total_nucl_sd)

Model = adonis(formula = n ~ total_nucl_sd, data = d16, permutations = 1000)

	Df	SumsOfSqs	MeanSqs	F.Model	R^2	Pr(>F)
**total_nucl_sd**	1	2.05375408880878	2.05375408880878	43.3156857023196	0.268515027002713	0.000999000999000999
**Residuals**	118	5.59480886773677	0.0474136344723455		0.731484972997287	
**Total**	119	7.64856295654555			1	

L.

PERMANOVA model using ST richness as *dependent variable* and the following independent variable: Standard deviation of the average GC% per genome (gc_avg_sd)

Model = adonis(formula = n ~ gc_avg_sd, data = d16, permutations = 1000)

	Df	SumsOfSqs	MeanSqs	F.Model	R^2	Pr(>F)
**gc_avg_sd**	1	1.58113983642368	1.58113983642368	30.7502043296177	0.20672377875514	0.000999000999000999
**Residuals**	118	6.06742312012187	0.0514188400010328		0.79327622124486	
**Total**	119	7.64856295654555			1	

Figure S7. PERMANOVA results measuring the association between species, program, or other genome-intrinsic and –extrinsic variables and ST richness. PERMANOVA results demonstrating the association (*R*-squared and *p*-values) between ST richness (n) and: (A) bacterial species and program (mlst vs.

stringMLST with all k-mer lengths); (B) bacterial species; (C) program (mlst vs. stringMLST with all k-mer lengths); (D) the median number of contigs (num_contigs_median); (E) the mean total number of nucleotides (total_nucl_mean); (F) the mean GC% content originally calculated per genome (gc_avg_mean); (G) the mean total count of STs present in each generated database (st_count_mean); (H) the mean total count of unique alleles (across all 7 loci) present in each generated database (total_alleles_genes_mean); (I) the Simpson's D index of diversity (simpson); (J) the standard deviation (SD) of the number of contigs (num_contigs_sd); (K) the SD of the total number of nucleotides (total_nucl_sd); (L) the SD of the GC% content per genome (gc_avg_sd). The median number of contigs, mean total number of nucleotides, and mean GC% content were grouped by species and batch (experimental replicate). The SD of the number of contigs, SD of the total number of nucleotides, and SD of GC% content were calculated by species only. The mean total count of STs and mean total count of unique alleles (across all 7 loci) present in each generated database were calculated after grouping by species, batch (three experimental replicates), and program. The Simpson's D index of diversity was calculated after grouping by program, species, and batch (three experimental replicates). All PERMANOVA models were run with 1,000 permutations.
