# Supplementary material for: Systems-Based Approach for Optimization of Assembly-Free Bacterial MLST Mapping
Source: Life (Basel). 2022 Apr 30;12(5):670. doi: 10.3390/life12050670 (PMC9147691; doi:10.3390/life12050670)
Supplement: Supplementary file 1 [file life-12-00670-s001.zip › Figure S8.pdf]

A.

PERMANOVA model using the Simpson's D index of diversity as *dependent variable* and the following independent variables: bacterial species (species) and program as MLST or stringMLST (program)

Model = adonis(formula = simpson ~ species * program, data = d13, permutations = 1000)

	Df	SumsOfSqs	MeanSqs	F.Model	R^2	Pr(>F)
**species**	3	0.410477977217033	0.136825992405678	2872.17128004194	0.990689005884547	0.000999000999000999
**program**	9	1.11486912828251E-05	1.23874347586946E-06	0.0260029792013696	2.690737748899E-05	1
**species:program**	27	3.56483293753596E-05	1.32030849538369E-06	0.0277151444295248	8.60372783693751E-05	1
**Residuals**	80	0.00381108169575958	4.76385211969947E-05		0.00919804945959517	
**Total**	119	0.41433585593345			1	

B.

PERMANOVA model using the Simpson's D index of diversity as *dependent variable* and the following independent variable: bacterial species (species)

Model = adonis(formula = simpson ~ species, data = d13, permutations = 1000)

	Df	SumsOfSqs	MeanSqs	F.Model	R^2	Pr(>F)
**species**	3	0.410477977217033	0.136825992405678	4114.13014398661	0.990689005884547	0.000999000999000999
**Residuals**	116	0.00385787871641774	3.32575751415323E-05		0.0093109941154535	
**Total**	119	0.41433585593345			1	

C.

PERMANOVA model using the Simpson's D index of diversity as *dependent variable* and the following independent variable: program as MLST or stringMLST (program)

Model = adonis(formula = simpson ~ program, data = d13, permutations = 1000)

	Df	SumsOfSqs	MeanSqs	F.Model	R^2	Pr(>F)
**program**	9	1.11486912828301E-05	1.23874347587002E-06	0.000328876796299909	2.69073774890021E-05	1
**Residuals**	110	0.414324707242168	0.00376658824765607		0.999973092622511	
**Total**	119	0.41433585593345			1	

D.

PERMANOVA model using the Simpson's D index of diversity as *dependent variable* and the following independent variable: median of the number of contigs (num_contigs_median)

Model = adonis(formula = simpson ~ num_contigs_median, data = d13, permutations = 1000)

	Df	SumsOfSqs	MeanSqs	F.Model	R^2	Pr(>F)
**num_contigs_median**	1	0.163614770286817	0.163614770286817	77.004065470006	0.394884410662967	0.000999000999000999
**Residuals**	118	0.250721085646634	0.00212475496310707		0.605115589337033	
**Total**	119	0.41433585593345			1	

E.

PERMANOVA model using the Simpson's D index of diversity as *dependent variable* and the following independent variable: mean of the total counts for nucleotides per genomes (total_nucl_mean)

Model = adonis(formula = simpson ~ total_nucl_mean, data = d13, permutations = 1000)

	Df	SumsOfSqs	MeanSqs	F.Model	R^2	Pr(>F)
**total_nucl_mean**	1	0.00373280185890127	0.00373280185890127	1.07274072849536	0.00900912099555493	0.310689310689311
**Residuals**	118	0.410603054074549	0.00347968689893686		0.990990879004445	
**Total**	119	0.41433585593345			1	

F.

PERMANOVA model using the Simpson's D index of diversity as *dependent variable* and the following independent variable: mean of the average GC% per genome (gc_avg_mean)

Model = adonis(formula = simpson ~ gc_avg_mean, data = d13, permutations = 1000)

	Df	SumsOfSqs	MeanSqs	F.Model	R^2	Pr(>F)
**gc_avg_mean**	1	0.0717695925601677	0.0717695925601677	24.7216752715419	0.173215982957785	0.000999000999000999
**Residuals**	118	0.342566263373283	0.00290310392689223		0.826784017042215	
**Total**	119	0.41433585593345			1	

G.

PERMANOVA model using the Simpson's D index of diversity as *dependent variable* and the following independent variable: mean of the total counts of unique STs per program (st_count_mean)

Model = adonis(formula = simpson ~ st_count_mean, data = d13, permutations = 1000)

	Df	SumsOfSqs	MeanSqs	F.Model	R^2	Pr(>F)
**st_count_mean**	1	0.00642494016874258	0.00642494016874258	1.85859929364807	0.0155065994813988	0.163836163836164
**Residuals**	118	0.407910915764708	0.00345687216749752		0.984493400518601	
**Total**	119	0.41433585593345			1	

H.

PERMANOVA model using the Simpson's D index of diversity as *dependent variable* and the following independent variable: mean of the total counts of unique alleles across all genes per program (total_alleles_genes_mean)

Model = adonis(formula = simpson ~ total_alleles_genes_mean, data = d13, permutations = 1000)

	Df	SumsOfSqs	MeanSqs	F.Model	R^2	Pr(>F)
**total_alleles_genes_mean**	1	6.56768036007165E-05	6.56768036007165E-05	0.0187072669366709	0.000158511030750053	0.879120879120879
**Residuals**	118	0.41427017912985	0.00351076422991398		0.99984148896925	
**Total**	119	0.41433585593345			1	

I.

PERMANOVA model using the Simpson's D index of diversity as *dependent variable* and the following independent variable:
Standard deviation of the number of contigs (num_contigs_sd)

Model = adonis(formula = simpson ~ num_contigs_sd, data = d14, permutations = 1000)

	Df	SumsOfSqs	MeanSqs	F.Model	R^2	Pr(>F)
**num_contigs_sd**	1	0.184650012452613	0.184650012452613	94.8630579020699	0.445652988531776	0.000999000999000999
**Residuals**	118	0.229685843480837	0.00194649019899014		0.554347011468224	
**Total**	119	0.41433585593345			1	

J.

PERMANOVA model using the Simpson's D index of diversity as *dependent variable* and the following independent variable:
Standard deviation of the total counts for nucleotides per genomes (total_nucl_sd)

Model = adonis(formula = simpson ~ total_nucl_sd, data = d14, permutations = 1000)

	Df	SumsOfSqs	MeanSqs	F.Model	R^2	Pr(>F)
**total_nucl_sd**	1	0.220300024808541	0.220300024808541	133.972178111131	0.531694328776423	0.000999000999000999
**Residuals**	118	0.19403583112491	0.0016443714502111		0.468305671223577	
**Total**	119	0.41433585593345			1	

K.

PERMANOVA model using the Simpson's D index of diversity as *dependent variable* and the following independent variable:
Standard deviation of the average GC% per genome (gc_avg_sd)

Model = adonis(formula = simpson ~ gc_avg_sd, data = d14, permutations = 1000)

	Df	SumsOfSqs	MeanSqs	F.Model	R^2	Pr(>F)
**gc_avg_sd**	1	0.0712010372963118	0.0712010372963118	24.4851934127079	0.171843774263524	0.000999000999000999
**Residuals**	118	0.343134818637139	0.00290792219184016		0.828156225736476	
**Total**	119	0.41433585593345			1	

Figure S8. PERMANOVA results measuring the association between species, program, or other genome-intrinsic and –extrinsic variables and the Simpson's D index of diversity.

PERMANOVA results demonstrating the association (*R*-squared and *p*-values) between the Simpson's D index of diversity (simpson) and: (A) bacterial species and program (mlst vs. stringMLST with all k-mer lengths); (B) bacterial species; (C) program (mlst vs. stringMLST with all k-mer lengths); (D) the median number of contigs (num_contigs_median); (E) the mean total number of nucleotides (total_nucl_mean); (F) the mean GC% content originally calculated per genome (gc_avg_mean); (G) the mean total count of STs present in each generated database (st_count_mean); (H) the mean total count of unique alleles (across all 7 loci) present in each generated database (total_alleles_genes_mean); (I) the standard deviation (SD) of the number of contigs (num_contigs_sd); (J) the SD of the total number of nucleotides (total_nucl_sd); (K) the SD of the GC% content per genome (gc_avg_sd). The median number of contigs, mean total number of nucleotides, and mean GC% content were grouped by species and batch (experimental replicate). The SD of the number of contigs, SD of the total number of nucleotides, and SD of GC% content were calculated by species only. The mean total count of STs and mean total count of unique alleles (across all 7 loci) present in each generated database were calculated after grouping by species, batch (three experimental replicates), and program. The Simpson's D index of diversity was calculated after grouping by program, species, and batch (three experimental replicates). All PERMANOVA models were run with 1,000 permutations.
