# Supplementary material for: Systems-Based Approach for Optimization of Assembly-Free Bacterial MLST Mapping
Source: Life (Basel). 2022 Apr 30;12(5):670. doi: 10.3390/life12050670 (PMC9147691; doi:10.3390/life12050670)
Supplement: Supplementary file 1 [file life-12-00670-s001.zip › Figure S9.pdf]

A.

PERMANOVA model using the proportion of non-classified STs as *dependent variable* and the following independent variables: bacterial species (species) and program as MLST or stringMLST (program)

Model = adonis(formula = prop ~ species * program, data = d18, permutations = 1000)

	Df	SumsOfSqs	MeanSqs	F.Model	R^2	Pr(>F)
**species**	3	3.34480401068386	1.11493467022795	23.3264912660022	0.324922836900245	0.000999000999000999
**program**	9	0.681352798076	0.0757058664528889	1.58390646533342	0.0661883576357917	0.0719280719280719
**species:program**	27	2.44423752682071	0.0905273158081743	1.89399854352207	0.237439499813996	0.002997002997003
**Residuals**	80	3.82375440014142	0.0477969300017678		0.371449305649967	
**Total**	119	10.294148735722			1	

B.

PERMANOVA model using the proportion of non-classified STs as *dependent variable* and the following independent variable: bacterial species (species)

Model = adonis(formula = prop ~ species, data = d18, permutations = 1000)

	Df	SumsOfSqs	MeanSqs	F.Model	R^2	Pr(>F)
**species**	3	3.34480401068386	1.11493467022795	18.610736244018	0.324922836900245	0.000999000999000999
**Residuals**	116	6.94934472503813	0.0599081441813632		0.675077163099755	
**Total**	119	10.294148735722			1	

C.

PERMANOVA model using the proportion of non-classified STs as *dependent variable* and the following independent variable:
program as MLST or stringMLST (program)

Model = adonis(formula = prop ~ program, data = d18, permutations = 1000)

	Df	SumsOfSqs	MeanSqs	F.Model	R^2	Pr(>F)
**program**	9	0.681352798076	0.0757058664528889	0.866308341905474	0.0661883576357917	0.631368631368631
**Residuals**	110	9.61279593764599	0.0873890539785999		0.933811642364208	
**Total**	119	10.294148735722			1	

D.

PERMANOVA model using the proportion of non-classified STs as *dependent variable* and the following independent variable:
median of the number of contigs (num_contigs_median)

Model = adonis(formula = prop ~ num_contigs_median, data = d18, permutations = 1000)

	Df	SumsOfSqs	MeanSqs	F.Model	R^2	Pr(>F)
**num_contigs_median**	1	2.78200760329276	2.78200760329276	43.6995114177773	0.270251351006698	0.000999000999000999
**Residuals**	118	7.51214113242923	0.0636622129866884		0.729748648993302	
**Total**	119	10.294148735722			1	

E.

PERMANOVA model using the proportion of non-classified STs as *dependent variable* and the following independent variable: mean of the total counts for nucleotides per genomes (total_nucl_mean)

Model = adonis(formula = prop ~ total_nucl_mean, data = d18, permutations = 1000)

	Df	SumsOfSqs	MeanSqs	F.Model	R^2	Pr(>F)
**total_nucl_mean**	1	0.312873903121628	0.312873903121628	3.69883819327053	0.0303933730854224	0.035964035964036
**Residuals**	118	9.98127483260036	0.0845870748525455		0.969606626914578	
**Total**	119	10.294148735722			1	

F.

PERMANOVA model using the proportion of non-classified STs as *dependent variable* and the following independent variable: mean of the average GC% per genome (gc_avg_mean)

Model = adonis(formula = prop ~ gc_avg_mean, data = d18, permutations = 1000)

	Df	SumsOfSqs	MeanSqs	F.Model	R^2	Pr(>F)
**gc_avg_mean**	1	0.235317166826526	0.235317166826526	2.7605021015954	0.022859312884216	0.0659340659340659
**Residuals**	118	10.0588315688955	0.0852443353296226		0.977140687115784	
**Total**	119	10.294148735722			1	

G.

PERMANOVA model using the proportion of non-classified STs as *dependent variable* and the following independent variable: mean of the total counts of unique STs per program (st_count_mean)

Model = adonis(formula = prop ~ st_count_mean, data = d18, permutations = 1000)

	Df	SumsOfSqs	MeanSqs	F.Model	R^2	Pr(>F)
**st_count_mean**	1	0.0631382909852366	0.0631382909852366	0.728209435079862	0.00613341545825336	0.472527472527473
**Residuals**	118	10.2310104447368	0.0867034783452267		0.993866584541747	
**Total**	119	10.294148735722			1	

H.

PERMANOVA model using the proportion of non-classified STs as *dependent variable* and the following independent variable: mean of the total counts of unique alleles across all genes per program (total_alleles_genes_mean)

Model = adonis(formula = prop ~ total_alleles_genes_mean, data = d18, permutations = 1000)

	Df	SumsOfSqs	MeanSqs	F.Model	R^2	Pr(>F)
**total_alleles_genes_mean**	1	0.835211515433743	0.835211515433743	10.4192423023798	0.0811345878980215	0.001998001998002
**Residuals**	118	9.45893722028825	0.080160484917697		0.918865412101978	
**Total**	119	10.294148735722			1	

I.

PERMANOVA model using the proportion of non-classified STs as *dependent variable* and the following independent variable:
Simpson's D index of diversity per species (simpson)

Model = adonis(formula = prop ~ simpson, data = d18, permutations = 1000)

	Df	SumsOfSqs	MeanSqs	F.Model	R^2	Pr(>F)
**simpson**	1	2.37335228285581	2.37335228285581	35.3569961106178	0.230553525481906	0.000999000999000999
**Residuals**	118	7.92079645286619	0.0671253936683575		0.769446474518094	
**Total**	119	10.294148735722			1	

J.

PPERMANOVA model using the proportion of non-classified STs as *dependent variable* and the following independent variable:
Standard deviation of the number of contigs (num_contigs_sd)

Model = adonis(formula = prop ~ num_contigs_sd, data = d18b, permutations = 1000)

	Df	SumsOfSqs	MeanSqs	F.Model	R^2	Pr(>F)
**num_contigs_sd**	1	0.343617214899233	0.343617214899233	4.07484075330651	0.0333798572102265	0.022977022977023
**Residuals**	118	9.95053152082276	0.0843265383120573		0.966620142789774	
**Total**	119	10.294148735722			1	

K.

PERMANOVA model using the proportion of non-classified STs as *dependent variable* and the following independent variable:
Standard deviation of the total counts for nucleotides per genomes (total_nucl_sd)

Model = adonis(formula = prop ~ total_nucl_sd, data = d18b, permutations = 1000)

	Df	SumsOfSqs	MeanSqs	F.Model	R^2	Pr(>F)
**total_nucl_sd**	1	2.51052137919844	2.51052137919844	38.0595716079769	0.243878483170407	0.000999000999000999
**Residuals**	118	7.78362735652355	0.0659629436993521		0.756121516829593	
**Total**	119	10.294148735722			1	

L.

PERMANOVA model using the proportion of non-classified STs as *dependent variable* and the following independent variable:
Standard deviation of the average GC% per genome (gc_avg_sd)

Model = adonis(formula = prop ~ gc_avg_sd, data = d18b, permutations = 1000)

	Df	SumsOfSqs	MeanSqs	F.Model	R^2	Pr(>F)
**gc_avg_sd**	1	0.67622148287334	0.67622148287334	8.29639618613466	0.0656898885215022	0.002997002997003
**Residuals**	118	9.61792725284865	0.0815078580749886		0.934310111478498	
**Total**	119	10.294148735722			1	

Figure S9. PERMANOVA results measuring the association between species, program, or other genome-intrinsic and –extrinsic variables and the proportion of non-classified STs.
PERMANOVA results demonstrating the association (*R*-squared and *p*-values) between non-classified STs (prop) and: (A) bacterial species and program (mlst vs.

stringMLST with all k-mer lengths); (B) bacterial species; (C) program (mlst vs. stringMLST with all k-mer lengths); (D) the median number of contigs (num_contigs_median); (E) the mean total number of nucleotides (total_nucl_mean); (F) the mean GC% content originally calculated per genome (gc_avg_mean); (G) the mean total count of STs present in each generated database (st_count_mean); (H) the mean total count of unique alleles (across all 7 loci) present in each generated database (total_alleles_genes_mean); (I) the Simpson's D index of diversity (simpson); (J) the standard deviation (SD) of the number of contigs (num_contigs_sd); (K) the SD of the total number of nucleotides (total_nucl_sd); (L) the SD of the GC% content per genome (gc_avg_sd). The median number of contigs, mean total number of nucleotides, and mean GC% content were grouped by species and batch (experimental replicate). The SD of the number of contigs, SD of the total number of nucleotides, and SD of GC% content were calculated by species only. The mean total count of STs and mean total count of unique alleles (across all 7 loci) present in each generated database were calculated after grouping by species, batch (three experimental replicates), and program. The Simpson's D index of diversity was calculated after grouping by program, species, and batch (three experimental replicates). All PERMANOVA models were run with 1,000 permutations.
