# Supplementary material for: Systems-Based Approach for Optimization of Assembly-Free Bacterial MLST Mapping
Source: Life (Basel). 2022 Apr 30;12(5):670. doi: 10.3390/life12050670 (PMC9147691; doi:10.3390/life12050670)
Supplement: Supplementary file 1 [file life-12-00670-s001.zip › Table S1.pdf]

**Serovar**	**Number of genomes downloaded from NCBI-SRA**
Salmonella Agona	565
Salmonella Anatum	600
Salmonella Braenderup	600
Salmonella Derby	590
Salmonella Dublin	600
Salmonella Enteritidis	600
Salmonella Hadar	600
Salmonella Heidelberg	600
Salmonella Infantis	600
Salmonella Javiana	600
Salmonella Johannesburg	534
Salmonella Kentucky	600
Salmonella Mbandaka	535
Salmonella Montevideo	600
Salmonella Muenchen	600
Salmonella Newport	600
Salmonella Schwarzengrund	600
Salmonella Senftenberg	563
Salmonella Thompson	600
Salmonella Typhimurium	600

Table S1. Total counts of downloaded genomes per serovar of *S. enterica* Subspecies *enterica* Lineage I. All downloaded genomes were freely available at NCBI-SRA.
