# Supplementary material for: Systems-Based Approach for Optimization of Assembly-Free Bacterial MLST Mapping
Source: Life (Basel). 2022 Apr 30;12(5):670. doi: 10.3390/life12050670 (PMC9147691; doi:10.3390/life12050670)
Supplement: Supplementary file 1 [file life-12-00670-s001.zip › Table S2.pdf]

Program	Version	Description	Databases	Link	Reference
**parallel-fastq-dump**	0.6	Parallel wrapper for SRA Toolkit	No	https://github.com/rvalieris/parallel-fastq-dump	[58]
**Trimmo-matic**	0.38	Trimming tool for Illumina NGS reads	No	https://github.com/timflutre/trimmomatic	[50]
**FastQC**	0.11	Tool to quality control for sequencing data	No	https://github.com/s-andrews/FastQC	[51]
**SPAdes**	3.13	Genome assembler	No	https://github.com/ablab/spades	[52]
**QUAST**	5.0	Evaluation tool for genome assembly	No	https://github.com/ablab/quast	[53]
**MLST**	2.16.2	Tool for multilocus-sequence typing	Integrated set of PubMLST databases for multiple organisms that can also be customized.	https://github.com/tseemann/mlst	[22]
**stringMLST**	0.6.3	Tool from multilocus-sequence typing of raw genome sequencing reads	Integrated set of PubMLST databases for multiple organisms that can also be customized.	https://github.com/jordanlab/stringMLST	[17]
**AMOS**	3.1	Collection of tools for genome assembly and related statistics	No	http://amos.sourceforge.net/wiki/index.php/AMOS	[54]
**EMBOSS**	6.6	European Molecular Biology Open Software Suite that contains multiple	No	http://emboss.open-bio.org/	[55]

		bioinformatics functionalities			
**DSK**	2.2.0	Tool for counting k-mers from reads or genomes	No	https://github.com/GATB/dsk/	[56]
**GNU Bash**	4.4.20	Unix shell and command tool	No	https://www.gnu.org/software/bash/	

Table S2. Description of bioinformatics programs used including their versions, available databases, links and references.
