# Supplementary material for: Systems-Based Approach for Optimization of Assembly-Free Bacterial MLST Mapping
Source: Life (Basel). 2022 Apr 30;12(5):670. doi: 10.3390/life12050670 (PMC9147691; doi:10.3390/life12050670)
Supplement: Supplementary file 1 [file life-12-00670-s001.zip › Table S3.pdf]

L. monocytogenes			*C. jejuni*			*S. enterica*			*S. aureus*		
**gene/locus name**	**number of genes/loci**		**gene/locus name**	**number of genes/loci**		**gene/locus name**	**number of genes/loci**		**gene/locus name**	**number of genes/loci**	
	**string MLST**	**mlst**		**string MLST**	**mlst**		**string MLST**	**mlst**		**string MLST**	**mlst**
**abcZ**	423	255	**aspA**	527	489	**aroC**	1024	813	**arcC**	761	621
**bglA**	373	220	**glnA**	712	655	**dnaN**	1015	804	**aroE**	944	779
**cat**	402	241	**gltA**	608	563	**hemD**	949	765	**glpF**	845	705
**dapE**	535	308	**glyA**	806	733	**hisD**	1428	1132	**gmk**	506	399
**dat**	315	200	**pgm**	1082	969	**purE**	1115	848	**pta**	782	641
**ldh**	623	461	**tkt**	829	761	**sucA**	1037	839	**tpi**	721	579
**lhkA**	360	218	**uncA**	628	565	**thrA**	1206	952	**yqiL**	872	694
	**Total number of STs**	**Total number of STs**		**Total number of STs**	**Total number of STs**		**Total number of STs**	**Total number of STs**		**Total number of STs**	**Total number of STs**
**ST database**	2323	1504		11167	9745		6992	5151		6447	5219

Table S3. Distribution of counts for each allele across all seven loci per bacterial species and program (mlst vs. stringMLST), including the total count of unique STs present in the ST scheme available in the program.
