# Supplementary material for: Systems-Based Approach for Optimization of Assembly-Free Bacterial MLST Mapping
Source: Life (Basel). 2022 Apr 30;12(5):670. doi: 10.3390/life12050670 (PMC9147691; doi:10.3390/life12050670)
Supplement: Supplementary file 1 [file life-12-00670-s001.zip › Table S4.pdf]

**Organism**	**Number of raw Illumina reads**	**Number of Illumina reads after filtering**	**Number of reads after MLST**
Salmonella Agona	100	100	100
Salmonella Anatum	100	100	100
Salmonella Braenderup	100	100	100
Salmonella Derby	100	100	100
Salmonella Dublin	100	100	100
Salmonella Enteritidis	100	99	99
Salmonella Hadar	100	100	100
Salmonella Heidelberg	100	100	100
Salmonella Infantis	100	100	100
Salmonella Javiana	100	100	100
Salmonella Johannesburg	100	100	100
Salmonella Kentucky	100	100	100
Salmonella Mbandaka	100	100	100
Salmonella Montevideo	100	100	100
Salmonella Muenchen	100	96	96
Salmonella Newport	100	98	98
Salmonella Schwarzengrund	100	100	100
Salmonella Senftenberg	100	100	100
Salmonella Thompson	100	100	100
Salmonella Typhimurium	100	100	100
Acineto baumannii	1000	901	343
Campylobacter jejuni	1000	996	995
Clostridium difficile	1000	839	833
Enterococcus faecium	1000	915	913
Escherichia coli	1000	895	895
Haemophilus influenzae	1000	951	949
Helicobacter pylori	1000	874	874
Klebsiella pneumoniae	1000	920	918
Listeria monocytogenes	1000	998	996
Mycobacterium tuberculosis	1000	809	790
Neisseria gonorrhoeae	1000	915	915
Pseudomonas aeruginosa	1000	956	954
Staphylococcus aureus	1000	998	998
Streptococcus pneumoniae	1000	940	940
Enterococcus faecalis	100	92	92
Enterococcus faecium	100	92	92
Helicobacter pylori	100	81	81

Table S4. Number of reads used for calculating percentage of agreement between mlst and stringMLST for 23 *Salmonella enterica* serovars and 14 phylogenetic divergent bacterial pathogens.

The datasets used to calculate percentage of agreement contained 100 or 1,000 paired-end raw Illumina reads downloaded from NCBI-SRA (first column in the Table). Before running mlst, a few steps were required, such as quality trimming and adapter clipping, *de novo* assembly and assembly filtering. After the filtering steps were completed, a fraction of all genomes were discarded from the mlst analyses. This number is shown in the second column in the Table. Additionally, mlst infrequently adds empty columns in its results, and these records were removed before conducting the final statistical analyses to calculate the percentage of agreement between programs (third column in the Table). All downloaded genomes were freely available at NCBI-SRA.
