# Supplementary material for: Systems-Based Approach for Optimization of Assembly-Free Bacterial MLST Mapping
Source: Life (Basel). 2022 Apr 30;12(5):670. doi: 10.3390/life12050670 (PMC9147691; doi:10.3390/life12050670)
Supplement: Supplementary file 1 [file life-12-00670-s001.zip › Text S1.pdf]

Factors that can influence ST-based classification

First, we describe the characteristics and composition of the data utilized for comparison between programs regarding ST-based classification in the narrow-scope approach (utilization of fewer phylogenetic diverse pathogen datasets). The frequency of genomes utilized per species and across programs is shown on Figure S2A-D. The frequency of *S. enterica* genomes was higher than other species because an equal sample of ~600 genomes was taken from 20 representative zoonotic serovars (Figure S3A-D). An assessment of the proportion of the most dominant STs across species (proportion ≥ 2% - Figure S4A-D) or serovars of *S. enterica* (proportion ≥ 15% - Figure S3N) initially revealed a similar ST-based distribution across programs. Furthermore, genome-intrinsic and -extrinsic factors that could potentially impact the mlst vs. stringMLST algorithmic comparison and performance were *a priori* determined in the analysis. Among the genome-intrinsic factors considered across species were the number of contigs per genome (Figure S5A), the total number of nucleotides per genome (Figure S5B), GC% content per genome (Figure S5C), and the distribution and composition of dinucleotides per species (Figure S5D and Figure S2E-F). Similarly, the distribution of the genome-intrinsic factors was analyzed across all twenty serovars of *S. enterica* (Figure S3G-L). A correlogram (pairwise correlation analysis) was also used to assess the bivariate correlation (Pearson's correlation coefficient) across genome-intrinsic variables, for either all four bacterial species (Figure S2G) or serovars across *S. enterica* (Figure S3M). At large, the differences observed in the distribution of genomic-intrinsic variables were species driven, with a strong uniformity found across serovars of *S. enterica*.

As for the genome-extrinsic variables, the total count of unique STs (for species - Figure S5E) and unique number of alleles across all seven loci (for species - Figure S5F), and across all batches were selected as factors that could influence the comparative analysis between mlst and stringMLST. Similarly, the genome-extrinsic variables were analyzed across all twenty serovars of *S. enterica* (Figure S3E-F). Of note, ST database/scheme size differences (number of STs and alleles) may directly influence the number of miscalls since it is expected that the larger the database is, the more likely STs are to be classified, or to find a match, and not be miscalled **Error! Reference source not found.**. Considering the differences in genome-intrinsic and -extrinsic variable distribution across species, such factors were further utilized for assessing their statistical contribution in the accuracy of ST-based classification between mlst and stringMLST.

Assessing the contribution of genome-intrinsic and –extrinsic variables

In order to assess the statistical association and contribution of each genomic-intrinsic and -extrinsic variable onto the accuracy of mlst vs. stringMLST on ST calls (narrow-scope analysis since it only included four bacterial species, *C. jejuni*, *S. aureus*, *L. monocytogenes*, and *S. enterica*), the following dependent variables (outcomes) were used in the PERMANOVA models: 1) ST richness (Figure S6A); 2) Simpson's D index of ST diversity (Figure S6B); and 3) Proportion of non-classified STs (Figure S6C). Additionally, the standard deviation of the proportion of non-classified STs was measured as an auxiliary metric for accuracy (Figure S6D). At the species level, a multivariate model approach was used to examine the interaction of species and program (mlst vs. stringMLST), whereas all remaining analyses were done using univariate models containing each genome-intrinsic and -extrinsic variable for all three outcomes (Figures S7A-L, S8A-K, S9A-L).

For each variable, the significance and strength of association were assessed by jointly examining the p-value ($p < 0.05$) and R-squared, respectively. For both ST richness (Figure S6A) and the Simpson's D index of diversity (Figure S6B), the difference between species explained most of the variation with ~98.3% and ~99%, respectively. As expected, based on the phylogenetic divergence of the four chosen pathogens, differences across species could largely be explained by

genome-intrinsic variables associated with genome composition, such as: GC% content (*p* ~0.0009, *R*-squared ~44%) for ST richness, and the number of contigs per genome (*p* ~0.0009, *R*-squared ~39.5%) for the Simpson's D index of diversity (Figure S6A-B). Notably, for both ST richness and the Simpson's D index of diversity most of the differences between species could be explained by variation in genome composition (Figure S6A-B). Not surprisingly, co-linearity was observed between ST richness and the Simpson's D index of diversity across species (Figure S6A). In the case of the proportion of non-classified STs (ST miscalls) (Figure S6C), most of the variation was explained by inter-species differences (*p* ~0.0009, *R*- squared ~33%), with the number of contigs per genome being the most important genome-intrinsic contributing factor (*p* ~0.0009, *R*- squared ~27%). As for the k-mer length parameter used by stringMLST, results for ST richness and the Simpson's D index of diversity were uniform across all lengths (Figure S6A-B). However, when examining the proportion of miscalls (Figure S6C) and the standard deviation of that proportion (Figure S6D), the data pointed toward the optimal k-mer length being between 35 and 65 across all four species due to the intrinsic variance within the *S. enterica* data (narrow scope analysis). Specifically, this k-mer length range was defined based on two criteria: i) minimization of the proportion of miscalls; and ii) less variation (standard deviation) around the average of ST-based miscalls. Of note, mlst demonstrated the highest proportion of miscalls and standard deviation of that proportion for both *L. monocytogenes* and *C. jejuni* (Figure S6C-D), and the k-mer length 10 for stringMLST yielded very low accuracy and null results for ST richness and Simpson's D index of diversity (Figure S6A-D). Differences between species across ST richness, Simpson's D index of diversity, and proportion of ST miscalls along with all genome-intrinsic and -extrinsic variables across programs (mlst vs. stringMLST) were further examined here (Figures S10A-D, S11A-O). In general, differences in ST-based calls across programs were largely influenced by the bacterial species dataset.

Given the complexity and diversity of the *S. enterica* population structure **Error! Reference source not found.**, the stringMLST performance was analyzed across twenty zoonotic serovars (Figure S3O-R), and resulted in a significant and predominant contribution of the "serovar groupings" across all outcomes and PERMANOVA models (Figures S12A-L, S13A-K, S14A-L): ST richness (*p* ~0.0009, *R*-squared ~75.4%), Simpson's D index of diversity (*p* ~0.0009, *R*-squared ~88%), and proportion of ST miscalls (*p* ~0.0009, *R*- squared ~35.4%). By assessing the distribution of the model outcomes, along with PERMANOVA model results and bivariate association between dependent and explanatory variables (Figure S15A-R), the results recapitulated the species-level results with the optimal k-mer length for stringMLST being around 35 and 65, but also revealed the need to consider difference across *S. enterica* serovars prior to implementation. Combined, these accuracy-based results suggest that: i) stringMLST minimizes the ST miscalls compared to mlst in a species-specific fashion, and by consequence the optimal k-mer length for stringMLST ranged from 35 to 65 overall; ii) the performance and accuracy of stringMLST can vary across species and serovars of *S. enterica* allowing for data-driven fine-tunning of the k-mer length; and iii) the use of sequence platform with longer reads, which would maximize the number of contigs per genome, could directly alter both mlst and stringMLST accuracy in ST calls across species.
